# Supplementary material for: High-resolution spatial prediction of anemia risk among children aged 6 to 59 months in low- and middle-income countries
Source: Commun Med (Lond). 2025 Mar 4;5:57. doi: 10.1038/s43856-025-00765-2 (PMC11880423; doi:10.1038/s43856-025-00765-2)
Supplement: Supplementary file 2 — Supplementary Information [file 43856_2025_765_MOESM2_ESM.pdf]

Supplementary Information: High-resolution spatial prediction of anemia risk among children aged 6 to 59 months in low- and middle-income countries

Johannes Seiler [1,2,3*], Mattias Wetscher [1], Kenneth Harttgen [4,5], Jürg Utzinger [6,7], and Nikolaus Umlauf [1]

[1] Department of Statistics, University of Innsbruck, Innsbruck, Austria
[2] School of Medicine and Health, Technical University of Munich, Munich, Germany
[3] Munich Center of Health Economics and Policy, Munich, Germany
[4] Development Economics Group, ETH Zurich, Zurich, Switzerland
[5] NADEL Center for Development and Cooperation, ETH Zurich, Zurich, Switzerland
[6] Swiss Tropical and Public Health Institute, Allschwil, Switzerland
[7] University of Basel, Basel, Switzerland

February 9, 2025

Supplementary Note 1 Overview of the Supplementary Information

This is the supplementary material for the manuscript *High-resolution spatial prediction of anemia risk among children aged 6 to 59 months in low- and middle-income countries* and is organized as follows. Supplementary Note 2 briefly discusses aetiology of anemia, its causative agents and correlates, and which covariates are considered appropriate proxies. Supplementary Method 1 describes the analytical framework in detail, providing additional insights into the five modeling steps. Supplementary Note 3, provides detailed information on the model validation and diagnostic model checks. Supplementary Note 4, provides computational details and information on the used software. Additional results, such as selection frequencies or the estimated prevalence of anemia stratified by sex are provided from Figure 16 onward.

*Corresponding author. E-Mail: johannes.seiler@uibk.ac.at

Supplementary Table 1: Source additional covariates. Source of additional covariates, including description, periodicity, and source.

Covariate	Description	Periodicity	Source (URL) and Reference
Altitude	Elevation in m	Static	NOAA ETOPO [1,2]
Conflict	1 if more than five conflict incidents have been reported within buffer, 0 otherwise	Annual	UCDP [3,4]
GDP	*log*(real per capita GDP)	Annual	WDI [5]
Land cover	Land cover classification	Annual	MODIS Land Cover [6,7]
Malaria risk	*Plasmodium falciparum* incidence	Annual	Malaria Atlas Project [8]
Malaria risk	*Plasmodium vivax* incidence	Annual	Malaria Atlas Project [9]
Malaria risk	*Plasmodium falciparum* temperature suitability	Static	Malaria Atlas Project [10]
Malaria risk	*Plasmodium vivax* temperature suitability	Static	Malaria Atlas Project [10]
Malaria risk	*Plasmodium falciparum* endemicity	Static	Malaria Atlas Project [11]
Malaria risk	*Plasmodium vivax* endemicity	Static	Malaria Atlas Project [12]
Night-time light	Night-time light	Annual	Night-time light [13]
NDVI	Normalized difference vegetation index	Monthly	GIMMS, MODIS [14–16]
Precipitation	Precipitation anomaly	Monthly	ERA5 [17]
Soil	Soil type	Annual	Soilgrids [18]
Temperature	2 m temperature anomaly	Monthly	ERA5 [17]
Travel Time	Motorised travel time to closest healthcare facility	Static	Malaria Atlas Project [19]
Travel Time	Walking only travel time to closest healthcare facility	Static	Malaria Atlas Project [19]
Travel Time	Global travel time to closest city	Static	Malaria Atlas Project [20]
Waterbody	Distance to body of water	Static	GRG Washington [21]

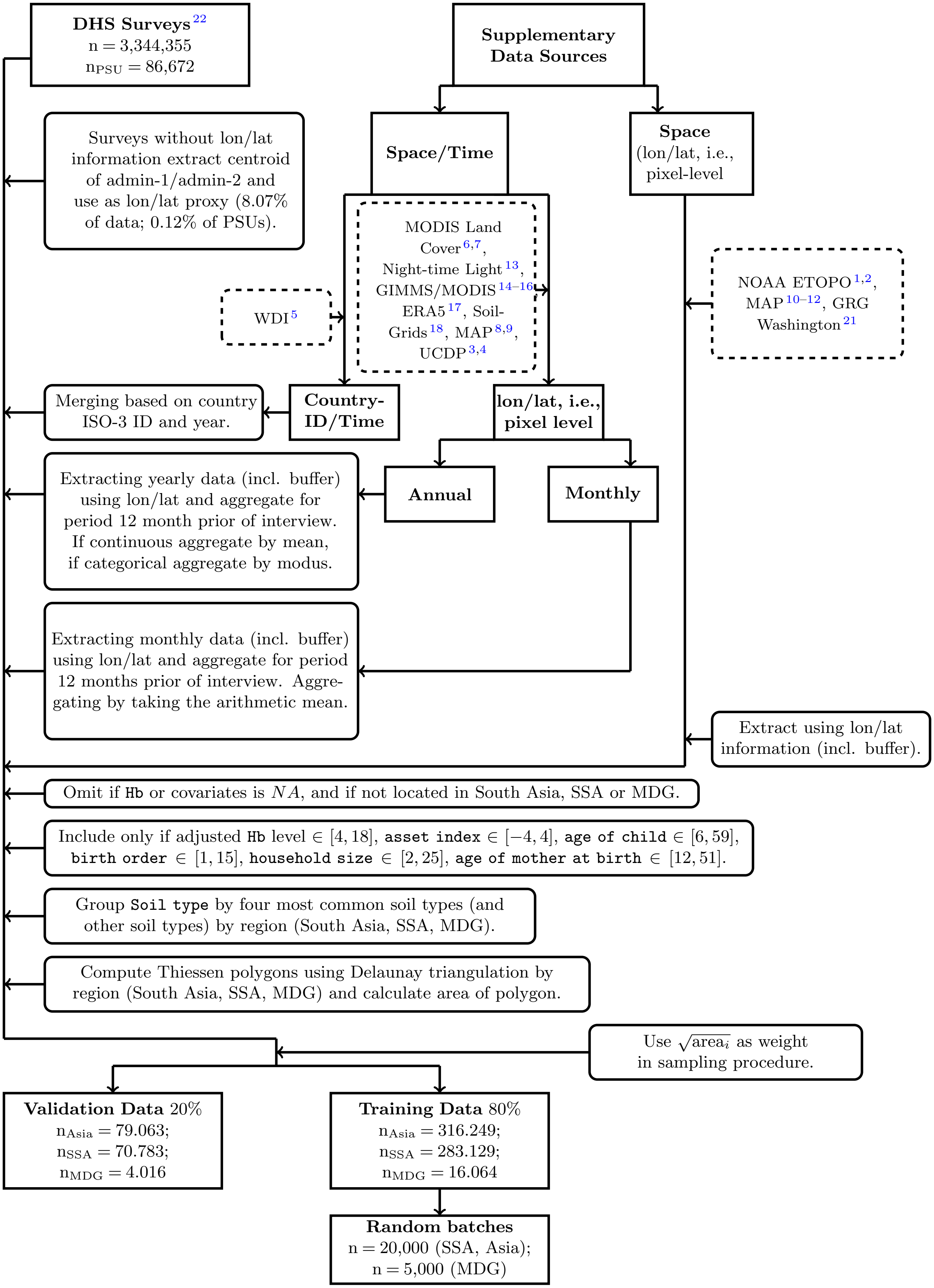

Supplementary Figure 1: Flowchart of data pre-processing. Note that malaria incidence was set to 0, if the country is classified as malaria free (e.g., Lesotho[23]).

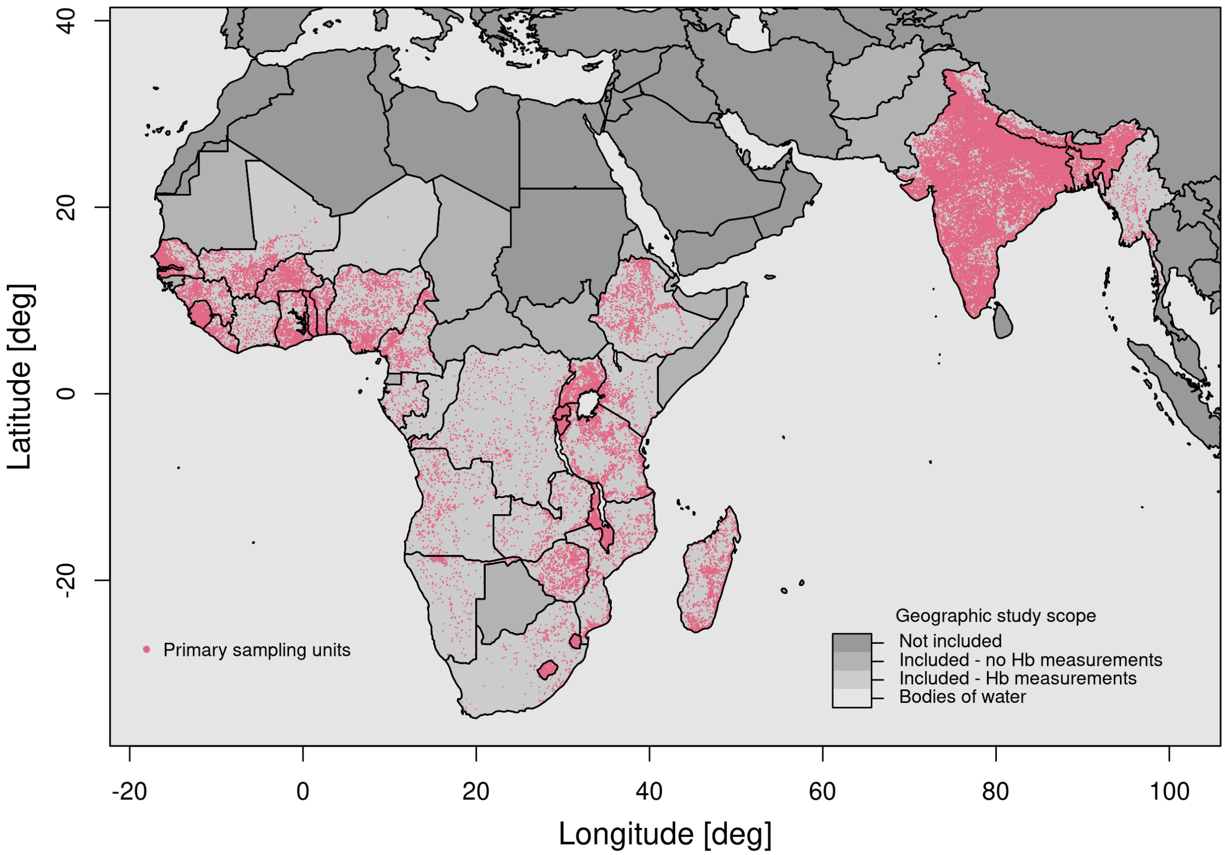

Supplementary Figure 2: Map of included countries and locations of the PSUs. Due to regional disconnectedness South Asia, sub-Saharan Africa, and Madagascar have been modeled separately. The gray-scaled colors indicate countries for which Hb measurements are available, countries without Hb measurements, however, we predict the Hb level and anemia prevalence and countries outside the study area that have been excluded.

Supplementary Note 2 Influencing factors

Elevation Remotely sensed information includes, for example, altitude, due to the pronounced relationship between the Hb level and altitude[24,25]. Routinely, for people living above an altitude of $1,000$ m, the Hb level is adjusted for altitude. This is due to the increased production of red blood cells at higher altitudes, which requires to add an adjustment factor to the observed measured Hb level for people living above $1,000$ m. We use satellite data from the National Oceanic and Atmospheric Administration (NOAA)[1,2] to add the altitude at the PSU to the study data.

Malaria Malaria is a major causative agent adding to the global health problem of anemia, with anemia being among the most common comorbidities of malaria[26]. One pathway how malaria causes anemia is when *Plasmodium* parasites that transmit malaria nest in the blood and reproduce in the erythrocytes, which destroys infected red blood cells and reduces the number of uninfected red blood cells[27–29]. For 2020, it is estimated that there were 241 million malaria cases globally and 630,000 malaria-related deaths with the majority being located in sub-Saharan Africa[30].[1] The MAP offers information on incidence, endemicity, and spatial limits of *Plasmodium falciparum*[8,11], and incidence, endemicity, and spatial limits of *P. vivax*[9,12]. The joint malaria incidence is calculated as the sum of *P. falciparum* and *P. vivax* incidence at locations with stable malaria transmission, extended by information on malaria endemicity and the spatial limits of malaria transmission.

Parasites Parasites such as hookworm or schistosomes are considered to be causative agents of anemia[31–33]. There are several distinct remotely sensed covariates that have been found to be associated with these parasites. For example, Brooker and colleagues[34] found that hookworm prevalence is correlated with surface temperature, altitude, and the normalized difference vegetation index (NDVI). Karigiannis-Voules et al.[35] highlighted a positive association between the prevalence of soil-transmitted helminths (e.g. hookworm, Ascaris lumbricoides, and Trichuris trichiura) and surface temperature and precipitation. In addition, hookworm larvae require specific temperature, soil, and aridity conditions to be able to hatch[36].
For example, Kokaliaris et al.[37] find the risk of schistosomiasis prevalence among children in sub-Saharan Africa to be linked to precipitation, NDVI, and the distance to the nearest body of fresh water. Similarly, Lai et al.[38], for example, highlight the importance of land cover classification, distance to the nearest body of water, or surface temperature on the risk of different Schistosoma species.

Thus elevation, the distance to the nearest body of water, land cover, precipitation, NDVI, and 2 m surface temperature are used as proxies for the prevalence of hookworm and schistosomes, see Supplementary Table 1 for the source of these covariates and Supplementary Table 3 for details on how these covariates were included.

Socio-demographic factors Several studies highlight the importance of socio-economic factors such as the age of the child, the household's wealth, the educational attainment of the mother or the household size as important associative factors of anemia[32,39,40]. The data on the socio-economic and demographic factors as additional covariates are taken from the DHS (Supplementary Table 1 and Supplementary Table 3).

[1] Six different species of *Plasmodium* exist that infect humans and are found to be among the causative agents of malaria and which are transmitted by female *Anopheles* mosquitos[29]. Dominant species of *Plasmodium* differ around the globe. For example, *P. falciparum* is the dominant species within sub-Saharan Africa and causes most fatalities[8,9,29]. However, in Madagascar, parts of Ethiopia, Eritrea, and South Sudan *P. vivax* is present, too[9]. In contrast, in Asia *P. vivax* is the dominant species, however, *P. falciparum* is also present irregularly[8,9]. Other subspecies are less common and *P. falciparum* and *P. vivax* add the largest share of fatal cases and humans falling sick with malaria[9,10].

Supplementary Table 2: **Summary statistics input data (i.e, training data and test data).** Median Hb level (g L^{-1}) and number of observations (n), by country, and survey year.

Country	Year	Median Hb	SD Hb	%Hb < 110 g L^{-1}	n	Country	Year	Median Hb	SD Hb	%Hb < 110 g L^{-1}	n
AGO	2007	106	14.4	58.6%	1,955	MLI	2018	97	15.6	81.7%	3,412
AGO	2011	109	14.3	51.5%	2,485	MMR	2015	108	13.7	55.4%	3,297
AGO	2016	105	14.4	64.5%	5,134	MOZ	2011	103	15.9	66.2%	4,139
BDI	2010	112	13.6	43.2%	2,910	MOZ	2015	105.5	16.6	60.6%	3,644
BDI	2012	110	15.7	49.9%	3,339	MOZ	2018	98	16.4	78.6%	3,544
BDI	2016	106	15.9	59.4%	5,149	MWI	2004	100	15.9	72.5%	1,997
BEN	2006	97	17	78.9%	3,804	MWI	2010	104	16.4	64.2%	4,068
BEN	2012	106	15.6	59.2%	3,014	MWI	2012	103	16.1	67.1%	1,850
BEN	2018	101	15.1	72.7%	5,040	MWI	2014	108	15.2	53.1%	1,708
BFA	2003	90	16.4	91.8%	2,473	MWI	2015	105	14.7	62.8%	4,427
BFA	2010	91	16.6	88.4%	5,552	MWI	2017	106	14.8	59.3%	1,916
BFA	2014	90	17.3	87.1%	5,383	NAM	2013	109	14.8	51.1%	1,474
BFA	2018	99.5	15.2	74.8%	4,670	NER	2006	95	17.4	82.6%	3,111
BGD	2011	109	12.3	52%	2,109	NER	2012	100	14.9	74.8%	4,349
CIV	2012	100	15.5	75.1%	2,658	NGA	2010	101	16.4	71.4%	3,978
CMR	2004	101	17.3	69.2%	2,839	NGA	2015	103	16.1	67.7%	5,038
CMR	2011	105	15.1	61.9%	4,426	NGA	2018	103	15.6	68.7%	9,684
CMR	2018	107	15.8	57.7%	3,864	NPL	2006	110	13.8	47.4%	4,688
COD	2007	102	16.7	69.7%	2,912	NPL	2011	111	13.6	46%	2,087
COD	2014	104	16.9	62.9%	6,449	NPL	2016	109	13.2	51.7%	2,067
COG	2005	105	14.9	64.3%	1,410	RWA	2005	109	16.2	50.8%	3,042
COG	2012	105	13.4	65.3%	3,578	RWA	2008	113	13.7	40.2%	4,058
ETH	2005	108	18.4	53.8%	3,240	RWA	2010	113	13.3	38.2%	3,541
ETH	2011	109	17.6	50%	8,090	RWA	2015	114	13.8	35.6%	3,054
ETH	2016	105	17	59.6%	7,378	RWA	2020	114	13.5	36.1%	3,275
GAB	2012	105	13.8	63.2%	2,610	SEN	2005	95	16.1	84%	1,947
GHA	2003	97	16.8	78.9%	2,621	SEN	2009	96	17.1	81%	2,884
GHA	2008	96	17.2	80.6%	1,949	SEN	2011	98	16.4	78.7%	2,644
GHA	2014	102	15.4	69.6%	2,200	SEN	2013	100	16.2	73.2%	4,064
GHA	2016	104	15.1	65.7%	2,399	SEN	2014	105	15.8	61.6%	4,366
GHA	2019	106	14.5	58.9%	2,066	SEN	2015	102	14.9	72%	4,300
GIN	2005	98	16.6	76.4%	2,134	SEN	2016	102.5	14.9	70%	4,430
GIN	2012	98	17.4	76.6%	2,596	SEN	2017	100	14.7	75.7%	7,508
GIN	2018	101	14.1	74.2%	2,815	SLE	2008	100	15.5	76.7%	1,642
GMB	2013	99	15.5	76.8%	2,350	SLE	2013	98	16.2	80.8%	3,839
GMB	2019	108	14.5	54.9%	2,435	SLE	2016	98	15.4	80.7%	4,541
IND	2006	104	15.6	64.8%	34,919	SLE	2019	102	14.9	70.3%	3,058
IND	2015	107	15	57.6%	202,730	SWZ	2007	112	15.2	42.1%	1,748
IND	2021	103	15.1	67.7%	143,415	TGO	2014	101	15.2	71.3%	2,740
KEN	2015	113	15.1	39.4%	2,759	TGO	2017	100	15.2	76.1%	2,766
KEN	2020	108	16.1	54%	2,842	TZA	2005	101	15.7	71.3%	6,303
LBR	2009	105	14.2	63.8%	2,606	TZA	2008	103	14.8	67.9%	4,750
LBR	2011	100	15.1	75.1%	2,131	TZA	2010	106	14.3	59.7%	5,157
LBR	2016	98	14.8	80.2%	1,882	TZA	2012	107	14.9	56.6%	5,982
LBR	2019	102	14.7	71.1%	1,909	TZA	2015	107	14.8	57.8%	7,016
LSO	2004	110	16.7	47.2%	1,065	TZA	2017	105	14.2	61.9%	5,738
LSO	2009	110	14.9	49.7%	1,419	UGA	2006	99	17.4	73.2%	1,778
LSO	2014	108	16.3	53.5%	1,150	UGA	2009	103	17.8	64.4%	2,622
MDG	2004	105	14.3	67.1%	1,394	UGA	2011	110	16.2	49.7%	1,719
MDG	2009	109	13.6	50.4%	4,349	UGA	2015	108	15.9	54%	3,466
MDG	2011	109	13.7	50.8%	4,724	UGA	2016	108	15.8	54.6%	3,550
MDG	2013	110	14.8	48.8%	4,106	UGA	2019	109	15.1	52%	4,756
MDG	2016	111	14	43.9%	5,507	ZAF	2016	105	16.1	61.5%	802
MLI	2006	95	17.6	80.7%	3,145	ZMB	2018	107	14.1	58.6%	7,377
MLI	2013	95	17.4	81.2%	4,021	ZWE	2006	107	13.6	57.9%	3,347
MLI	2015	94	16.6	85.9%	5,570	ZWE	2011	107	14.6	58.2%	3,245
MLI	2018	97	15.6	81.7%	3,412	ZWE	2015	113	13.5	38.2%	4,092

Summary statistic of the Hb level (in g L^{-1}) and the prevalence of anemia by country and year. Country abbreviations correspond to the country-specific ISO3 country codes. *S*ource: DHS data sets.

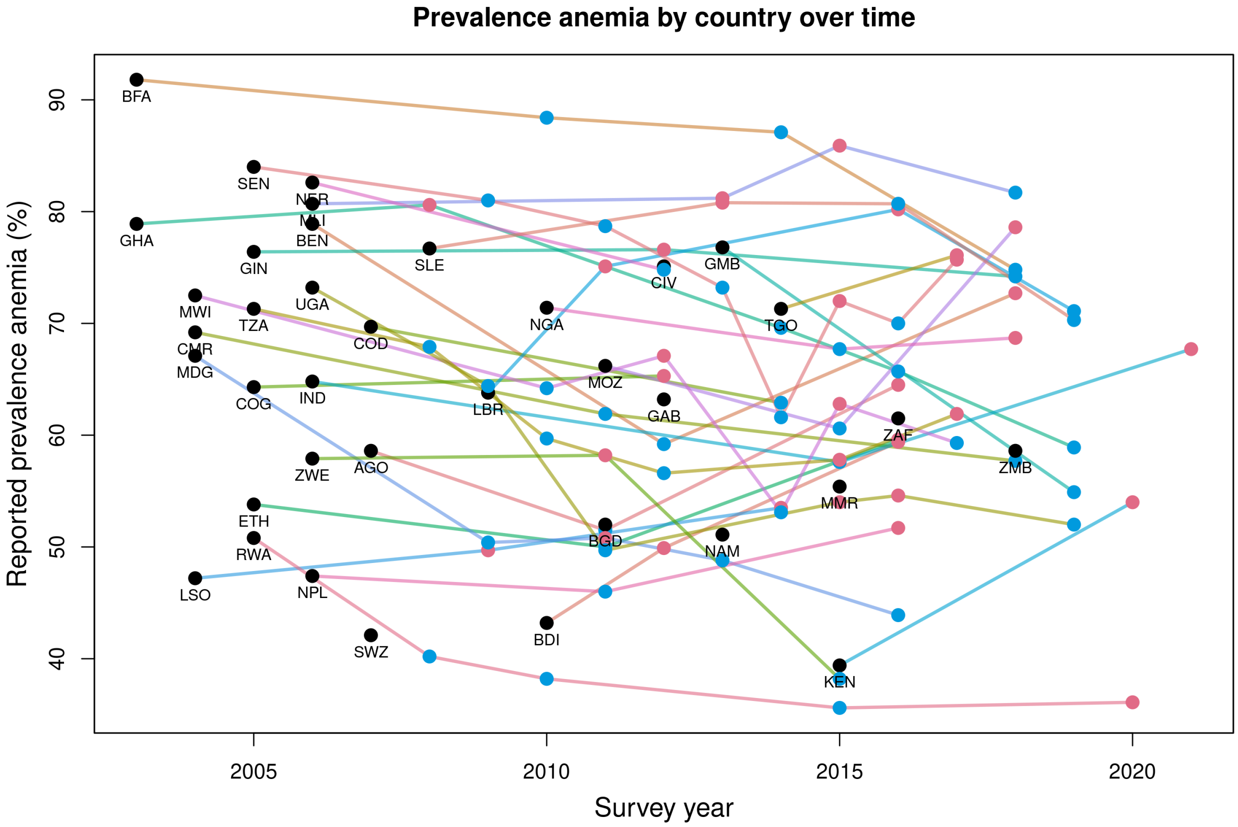

Supplementary Figure 3: Country-level time trend of the prevalence of any anemia (Hb < 110 g L^{-1})) for countries included in the analysis. Blue (red) dots indicate that compared to the previous survey the prevalence of any anemia decreased (increased).

Supplementary Method 1: Modelling Framework

Regression models explaining the prevalence of anemia

Generalized additive models (GAM) in both frequentist and Bayesian settings have been utilized in diverse applications across various fields of applied research[41,42], as well as to global health topics such as, the modeling and mapping of childhood mortality[43–45], or childhood malnutrition[46–48]. The model class is particularly useful in accounting for non-linear covariate effects, capturing group effects beyond a simple parallel shift, considering complex spatio-temporal interactions, and exhibiting good predictive performance. However, merely regressing the mean Hb level, as in[39], or[40] in the context of GAMs would not provide a comprehensive characterization of the prevalence of mild, moderate, and severe anemia, as well as other levels, based on the estimated model. This approach fails to specify the complete distribution, which is crucial for a thorough understanding of anemia.

In numerous applications[39,49], risk levels are therefore estimated using binomial models, which categorize the response by employing predetermined thresholds. Although the probability estimates derived from this approach are theoretically precise, one substantial drawback emerges when separate models are used for each risk level. Specifically, the probabilities do not add up to one, resulting in inaccurate predictions that may compromise the validity of the results.

To overcome this issue, ordered logistic regression models have been suggested as a potential remedy[50,51]. Two single-country studies were identified applying a variant of this approach[52,53]. However, the accuracy of this method may still be compromised due to the arbitrary reduction in response information that results from threshold-based categorization. Thus, alternative methods that avoid categorization and instead consider the response as a continuous variable should be explored to achieve more accurate risk level predictions. These methods should incorporate the full range of the response data and account for any non-linear relationships between predictors and the response variable to maximize the accuracy of risk level estimation.

Several distinct approaches exist to characterize the response distribution, such as quantile regression models[54], or distributional regression models, also known as generalized additive models for location, scale, and shape (GAMLSS)[55]. The first approach has the disadvantage of quantile crossing and the need to estimate individual models for each quantile, which is due to the large number of observations computationally challenging or may not be feasible, even with state-of-the-art methods and software[56,57]. The latter approach, in contrast, offers the advantage of characterizing the complete distribution based on a set of covariates. Furthermore, there are efficient methods for this class of models, which allows to deal with very large data sets like in this application[58,59].

The modeling approach employed in this study, as described by[60], can be briefly outlined as follows:

1. **Creating the training and test data sets**. Before estimating the models, the complete data set is per region split into a training data set, containing 80% of the data and a test data set containing approximately 20% of the data. Due to the spatial clustering, the data is not split randomly. Instead, the approach described by Gething and colleagues[11], which is based on Delaunay triangulation, is used to create the training and test data set. See Figure 1 for details of the individual pre-processing steps.

2. **Reducing the number of suitable candidate distributions**. Based on the descriptive analysis, the range of Hb levels, and by the fact that Hb levels in the DHS surveys are slightly skewed[25], suitable response distributions need to be continuous, suitable for continuous non-negative responses, and suitable for skewed data. Hence, to reduce the number of distributions under consideration, the first step is to estimate distribution models without covariate information (i.e., the "naive model") for each geographical region separately. Then, based on information criteria (e.g., the Akaike information criterion (AIC) or Bayesian information criterion (BIC)[61], the mean squared error (MSE), and the Continuous Rank Probability Score (CRPS)[62] evaluated on out-of-sample data, the three best distributions are selected for further analysis. In addition, the normal distribution is always included in the analysis as a benchmark.

3. **Defining candidate predictors η_k for each distributional parameter k**. In the next step, the relevant covariates for all considered distribution models are selected via a boosting algorithm for distribution regression[63–65].

4. **Re-estimating the final models omitting uninformative covariates**. Once the number of covariates is reduced to include only those that are informative for describing the full distribution of Hb levels, the final models are estimated for the distributions that are deemed most appropriate for modeling the Hb levels. Markov chain Monte Carlo (MCMC) simulation techniques are used to estimate these final models.

5. **Model evaluation and calibration checks**. To evaluate the predictive performance of the final models and to analyze model calibration on out-of-sample data, graphical tools, the MSE, and the CRPS are used. The candidate models are then compared to the "naive model" (without covariates) of the first step using skill score metrics[66]. Based on the best model for each continent, selected by the skill score, the results are interpreted and the spatio-temporal risk mappings for anemia are analyzed.

Distributional regression framework

Within the framework of GAMLSS or distributional regression[55,67] it is assumed that the response follows a parametric distribution (e.g., normal distribution, beta distribution, gamma distribution) and that each parameter can be modeled by covariates $\mathbf{x}, \mathbf{z}$ such that regression effects are incorporated for all parameters of the specified response distribution[60]. In more detail, within this framework all K parameters of the response distribution can be related to separate predictors, i.e.,

$$y|\mathbf{x},\mathbf{z} \sim \mathcal{D}_y\big(\theta_1(\mathbf{x},\mathbf{z}) = h_1^{-1}(\eta_1(\mathbf{x},\mathbf{z})), \theta_2(\mathbf{x},\mathbf{z}) = h_2^{-1}(\eta_2(\mathbf{x},\mathbf{z})), ..., \\ \theta_K(\mathbf{x},\mathbf{z}) = h_K^{-1}(\eta_K(\mathbf{x},\mathbf{z}))\big). \tag{1}$$

Here $\mathcal{D}_y$ specifies the parametric distributional family of the response y, $\theta_1(\mathbf{x},\mathbf{z}), ..., \theta_K(\mathbf{x},\mathbf{z})$ the distributional parameters, and $\mathbf{x}$ and $\mathbf{z}$ the covariate vectors that enter the model as linear and possibly non-linear smooth effects, respectively. The K $(k = 1, ..., K)$ parameters θ_K of distribution $\mathcal{D}_y$ are related to an additive predictor $\eta_k \equiv \eta_k(\mathbf{x},\mathbf{z})$ with separate suitable link functions h_k^{-1} to guarantee the potential restrictions of the parameter space. Accordingly, the predictor η_k of distributional parameter θ_k takes the following form,

$$\eta_k = \eta_k(\mathbf{x}, \boldsymbol{\gamma}; \mathbf{z}, \boldsymbol{\beta}_k) = \mathbf{x}^\top \boldsymbol{\gamma} + f_1(\mathbf{z}, \boldsymbol{\beta}_{1k}) + \ldots + f_{25}(\mathbf{z}, \boldsymbol{\beta}_{25k}), \tag{2}$$

where the predictor η_k can include different model terms, for example linear terms, non-linear smooth terms, or complex covariate interactions. In more detail, the structured additive predictors η_k (STAR)[68] as specified in this application include the following effects:

- Linear effects $\mathbf{x}^\top \boldsymbol{\gamma}$, which subsumes terms, entering the models linearly: the sex of observation i, indicators of whether conflicts have been reported within a specific buffer around the location of the PSU, the soil type found at the location of the PSU, and the land cover classification found at the location of the PSU.

- Univariate non-linear smooth functions $f_j(\cdot)$ modeled using thin-plate splines[69] of metrical socio-economic, demographic, and remotely sensed environmental covariates.

- Multivariate space-time and space-age interaction effects $f_j(\cdot)$ using the projected longitude and latitude coordinates of the PSU which are modeled by tensor product splines[70].

- In addition, to stratify the results by sex the spatio-temporal effects vary smoothly with the sex of the child, i.e., the complex space-time effects include varying coefficient terms[71], where the sex of the child is used as an effect modifier of the smooth space-time and space-age interaction.

However, the framework of distributional regression models allows to incorporate model terms beyond the specified terms. See Supplementary Table 3 for a complete overview of included covariates and the chosen functional form. Moreover, this table also summarizes the included complex space-time and space-age interactions.

Modeling approach The five steps of the modeling approach outlined in Supplementary Method 1 will be described in the following in more detail.

Creating the training and test data sets

Randomly splitting the data set into the training and validation data sets in the presence of a dependency structure (e.g., spatially or temporally clustered data) typically leads to estimation errors and unreliable error estimates, causing the results of the estimated models to be overly optimistic[72]. To help remedy this consequence of spatial and/or temporal clustering, one solution is to divide the data set into irregularly shaped blocks and draw the training and test data set from these blocks. An effective yet powerful method for constructing a validation data set (i.e., test data set) that is a representative sample of the prediction space is to construct a set of Thiessen polygons around each sampled location i and assign the resulting area of the polygon surrounding the location i (or a monotonic transformation such as the $\sqrt{\text{area}_i}$) as the sampling probability[11]. Thus the complete data set of each region is divided into a training data set – containing approximately 80% of the data – and a test data set – containing about 20% of the data – by assigning the sampling probability of each location to the data set and drawing (without replacement) the corresponding sample size. In addition, see Figure 1 for details of the individual pre-processing steps.

Reducing the number of suitable candidate distributions

Specifying the distribution correctly is important when considering complex distributional regression models. The distribution model selection problem is demonstrated in Supplementary Figure 4, which shows the histogram of Hb levels and distributions in sub-Saharan Africa. The data's asymmetry causes the normal distribution to overestimate the risk of moderate anemia and underestimate the risk of severe anemia. In contrast, characterizing the Hb levels using the more flexible skew exponential power type 3 (`SEP3`) distribution, this skewness is captured much more accurately and a more reliable model for predicting the anemia risk is provided. For example, applying this more flexible distribution results in differences of about 5% for moderate anemia.
Before considering the full model specification of the selection step – to narrow down the number of potential response distributions – a model omitting covariates, and incorporating only the intercept in the predictors η_k for each distributional parameter is estimated. In this step possible continuous distributions from the `R` package **gamlss.dist**[73] are used and imported into the `R` package **bamlss**[74] to estimate a full probabilistic distributional regression model. The estimated models are then compared based on the out-of-sample AIC[61], the MSE, and the CRPS[62]. Based on this procedure, the following distributions – besides the normal distribution, i.e., `NO`, which is used as benchmark distribution – are taken into consideration.

- Box-Cox-Cole-Green distribution, i.e., family `BCCG` (used to model Hb concentrations in Madagascar).
- Box-Cox t distribution, i.e., family `BCT` (used to model Hb concentrations in Madagascar).
- Exponential generalized Beta type 2 distribution, i.e., family `EGB2` (used to model Hb concentrations in South Asia, Madagascar and sub-Saharan Africa).
- Johnson's Su distribution, i.e., family `JSU` (used to model Hb concentrations in South Asia).
- Skew Power exponential type 3 distribution, i.e., family `SEP3` (used to model Hb concentrations in South Asia, Madagascar, and sub-Saharan Africa).
- Sinh-Arcsinh distribution, i.e., family `SHASHo` and family `SHASHo2` (used to model Hb concentrations in sub-Saharan Africa).
- Skew t type 3 distribution, i.e., family `ST3` (used to model Hb concentrations in South Asia)
- Skew t type 5 distribution, i.e., family `ST5` (used to model Hb concentrations in used to model Hb in South Asia, Madagascar, and sub-Saharan Africa).

See also Supplementary Figure 4, which illustrates besides the histogram of the Hb levels exemplary for sub-Saharan Africa, the fitted densities of the `NO` family and the `SEP3` family of the distribution of the Hb levels. Note the importance of the choice of the correct candidate distribution and how a misspecification of the distribution would result in an estimation error. The correct specification is also crucial to determine the

prevalence of anemia based on the chosen parametric distribution as precise as possible. Hence, as indicated in Supplementary Figure 4 the `NO` family can only be considered as a starting point and is included as a reference for benchmarking purposes. It is expected that more complex distributions – that explicitly allow to account for heavy tails and skewness – can be considered as more suitable distributions.

Defining candidate predictors η_k for each distributional parameter k

Non-informative covariates are detected by the application of a state-of-the-art backfitting algorithm described in[65]. Using this algorithm, only those model terms in each predictor η_k that make the largest contribution to the log-likelihood are selected. Here the full model of each predictor η_k is given by:

$$\begin{aligned}\eta_k = {} & \texttt{gf} \cdot \gamma_1 + \texttt{conf25} \cdot \gamma_2 + \texttt{conf50} \cdot \gamma_3 + \texttt{conf100} \cdot \gamma_4 + \texttt{lc12} \cdot \gamma_5 + \texttt{soil2} \cdot \gamma_6 + \\ & f_1(ai) + f_2(\texttt{bord}) + f_3(\texttt{cage}) + f_4(\texttt{fyvacn}) + f_5(\texttt{hhs}) + f_6(\texttt{higheduyear}) + \\ & f_7(\texttt{lgpd}) + f_8(\texttt{magebirth}) + f_9(\texttt{altitude}) + f_{10}(\texttt{ldistance}) + f_{11}(\texttt{lttcity}) + \\ & f_{12}(\texttt{lttmotor}) + f_{13}(\texttt{lttwalk}) + f_{14}(\texttt{minc12}) + f_{15}(\texttt{ndvi12}) + f_{16}(\texttt{nl20\_12}) + \\ & f_{17}(\texttt{pre12}) + f_{18}(\texttt{survey\_year}) + f_{19}(\texttt{t2m12}) + f_{20}(\texttt{x, y}) + f_{21}(\texttt{x, y}) \cdot (\texttt{gf}) + \\ & f_{22}(\texttt{x, y, cage}) + f_{23}(\texttt{x, y, cage}) \cdot (\texttt{gf}) + f_{24}(\texttt{x, y, survey\_year}) + \\ & f_{25}(\texttt{x, y, survey\_year}) \cdot (\texttt{gf}).\end{aligned} \tag{3}$$

See also Table 3 for a complete overview and explanation of included covariates and the chosen functional form. Moreover, this table also summarizes the included complex space-time interactions.

In more detail, the algorithm is based on the backfitting algorithm of Umlauf and colleagues[59], where each updating step is based on iteratively weighted least squares (IWLS)[75]. Then the updating scheme is repeated until a pre-specified termination criterion is met, i.e., the relative change of the estimated coefficients is below the defined threshold.

In contrast to the classical backfitting algorithm, in this variant[65], instead of using in each iteration the complete training data set, the evaluation of the model terms in each updating step is based on a random subset of the training data, which is different in each step. Accordingly, the training data set is partitioned into $T = 500$ randomly chosen batches $\mathbf{b}_1, \ldots, \mathbf{b}_T$, where samples may overlap (i.e., sampling with replacement), and in each step $t = 1, \ldots, T$ the backfitting algorithm computes potential updates on the batch $\mathbf{i} = \mathbf{b}_t$ and selects the best update corresponding to a single model term according to the best log-likelihood improvement on the next batch $\tilde{\mathbf{i}} = \mathbf{b}_{t+1}$ (if $t = T$, then the next batch is the first). Using two batches per iteration provides additional stability because the second batch $\tilde{\mathbf{i}}$ emulates out-of-sample data. Furthermore, computing the updates on a subset reduces the computational requirements drastically and introduces a stochastic updating equation for the $jk-$th model term of the following form, where $\nu = 0.1$ is a step length parameter,

$$\beta_{jk}^{[t+1]} = (1 - \nu) \cdot \beta_{jk}^{[t]} + \nu \cdot \beta_{[i],jk}. \tag{4}$$

Here in each step using the randomly chosen batches $\mathbf{i}$ and $\tilde{\mathbf{i}}$ from the training data, the algorithm loops through all distributional parameters (indexed over k) and within each distributional parameter through all model terms (indexed over j). Thus after the specified number of iterations uninformative model terms that have not been updated can be eliminated when re-estimating the model using MCMC sampling.

Re-estimating the final models omitting uninformative covariates

The final model – i.e., the model containing only those covariates in each predictor η_k that were selected in the previous step – is estimated using classical MCMC sampling techniques. For this purpose the predictors in Equation 3 include only those covariates that have been selected in the previous step. See Supplementary Figure 16 which covariates have been included in which predictor η_k. Analogously to the previous step, the predictors η_k of the distributional parameter $\theta_k(\mathbf{x}, \mathbf{z})$ are linked to a structured additive predictor using their common link function, which is monotonic and twice differentiable[59], i.e.,

$$\theta_k(\mathbf{x}, \mathbf{z}) = h_k(\eta_k^{\theta_k}). \tag{5}$$

The final model is then fitted using MCMC sampling schemes to estimate the regression coefficients $\boldsymbol{\gamma}_{jk}$ and $\boldsymbol{\beta}_{jk}$.

In this context, a common choice of priors for linear effects are uninformative normal priors for $\boldsymbol{\gamma}$, i.e., $p_{jk}(\gamma_{jk}|y) \sim \mathcal{N}(\mathbf{m}, \mathbf{M})$, where $\mathbf{m}$ is the prior mean and $\mathbf{M}$ is the prior covariance matrix. To ensure the prior is uninformative, a typical approach is to increase the variances in $\mathbf{M}$, or equivalently, to allow the precision matrix $\mathbf{M}^{-1}$ to approach zero. As the variance components in $\mathbf{M}$ become very large (or equivalently, as $\mathbf{M}^{-1} \to 0$), the prior becomes increasingly flat and resembles a uniform distribution in the limit. However, it's important to note that a normal prior with very large variance does not strictly become a uniform distribution, but rather a Gaussian distribution with a very broad spread. A key consideration is whether the uninformative prior is parameterized by the standard deviation σ or the variance σ^2. The choice of variance σ^2, as used here, ensures that the prior is weakly informative and very flat. This results in a proper, but uninformative, prior for the linear effects, which does not overly constrain the model. For further discussion of the choice of prior for linear effects, see, e.g.,[59,68].

For non-linear effects, which are typically based on a basis function approach, a multivariate normal prior for the basis function $\boldsymbol{\beta}_{jk}$, with a model term-specific precision matrix is used (see, e.g.,[59]). Thus the prior $p_{jk}(\cdot)$ of $\boldsymbol{\beta}_{jk}$ assumes a multivariate normal kernel of the following form: $d_{\boldsymbol{\beta}_{jk}}(\boldsymbol{\beta}_{jk}|\boldsymbol{\tau}_{jk}, \boldsymbol{\alpha}_{\boldsymbol{\beta}_{jk}}) \propto |\boldsymbol{P}_{jk}(\boldsymbol{\tau}_{jk})|^{\frac{1}{2}} \exp\left(-\frac{1}{2}\boldsymbol{\beta}_{jk}^{\top}\boldsymbol{P}_{jk}(\boldsymbol{\tau}_{jk})\boldsymbol{\beta}_{jk}\right)$. By specifying the penalty matrices $\boldsymbol{\alpha}_{\boldsymbol{\beta}_{jk}} = \{\boldsymbol{K}_{1jk}, ..., \boldsymbol{K}_{Ljk}\}$ for the basis function coefficients $\boldsymbol{\beta}_{jk}$ the precision matrix, e.g., for univariate splines $\boldsymbol{P}_{jk}(\boldsymbol{\tau}_{jk}) = \tau_{1jk}^{-2}\mathbf{K}_{1jk}$, can be obtained, where the penalty matrices are specific to the chosen smooth term specification. For the smoothing variances $\boldsymbol{\tau}_{jk}$ a inverse gamma distribution is used for each $\boldsymbol{\tau}_{jk} = (\tau_{1_{jk}}, ..., \tau_{L_{jk}})^{\top}$. In the latter case, smoothing variances, also sampled from the posterior distribution, control the amount of smoothness/overfitting, similar to the frequentist's smoothing parameter. The primary reason we use a Bayesian model is not to incorporate prior knowledge to improve the model but to achieve valid inference, which is almost impossible to obtain for distributional regression models using the frequentist approach. For details see e.g.,[59] for a more detailed description of the prior choice for non-linear effects. Generic multivariate normal priors for smooth functions with a precision matrix $\boldsymbol{P}_{jk}$ specific to the basis function $\boldsymbol{\beta}_{jk}$ have demonstrated high efficiency and robustness in numerous applications, (see, e.g.,[58,60,67,76,77]).

Model evaluation and calibration checks

Before discussing and interpreting the results of the final model, graphical diagnostic checks have been used to assess how well the final model has been calibrated and whether the model has been specified correctly. For this the following graphical tools have been used: Randomized quantile residuals[78], i.e., $\hat{r}_i = \Phi^{-1}\left(\mathcal{F}(y|\hat{\boldsymbol{\theta}})\right)$. Here Φ^{-1} is the inverse of the cumulative distribution of a standard normal distribution and the model is considered to be correctly specified when the randomized quantile residuals follow at least approximately a standard normal distribution. Similarly, the resulting residuals can be evaluated using the probability integral transform (PIT) histogram[79] with $u_i = \mathcal{F}(y|\hat{\boldsymbol{\theta}})$. A model is considered to be well calibrated if u_i is approximately close to a uniform distribution in the interval $[0, 1]$. As an additional diagnostic tool, the worm plot is used to check whether the distribution of the final model is well calibrated[80]. All diagnostic checks have been done on the out-of-sample test data set. This allows us to assess how well the model is calibrated on data that have not been used in both steps (variable selection and refitting) of the development of the final full probabilistic distributional model, which is used to predict the prevalence for all forms of anemia. These diagnostic plots are shown in Supplementary Figure 5 to 7. The comparison of the predictive ability of the competing models is assessed using the MSE and proper scoring rules[62] and calculating skill score metrics[66] based on the MSE and the CRPS to compare the models to the intercept-only model.

Robustness check for potential data sparseness issues – leave one administrative region out cross-validation

As a robustness check and to identify potential data sparseness issues, the final regional submodels were re-estimated using a cross-validation procedure where, depending on the region (i.e., South Asia, sub-Saharan Africa, and Madagascar), each admin-0 level region (sub-Saharan Africa), each admin-1 level region (Madagas-

car) and a combination of both (South Asia) were left out in a cross-validation-like procedure.[2] Accordingly, in the estimation process for each fold of the cross-validation the observations belonging to the specified administrative level regions were held out from the training data and the final model was estimated omitting these data. The model validation of each fold was carried out on, both the training and the testing data of the corresponding regions that has not been used in the estimation.

Together with the non-random splitting of the data set into the training and validation data sets, which is done due to the spatio-temporal dependency structure this cross-validation procedure helps to remedy potential data sparseness problems and to obtain valid estimates even in data-sparse areas.

[2]Please note that since only four countries (i.e., Bangladesh, India, Myanmar, and Nepal) were included in the regional model for South Asia, instead of leaving out India entirely, the cross-validation was done by leaving out admin-1 regions within India and the three consecutive countries. Note also that for Madagascar, the cross-validation was done by omitting admin-1 regions individually.

Supplementary Table 3: Covariates in each predictor η_k included in the full model of the selection stage used for the estimation of the distribution of Hb.

Model term	Unit of measurement	Type	Description
Response			
Hb level	g/dL	Continuous	Hb level at the day of the interview
Demographic covariates			
$\texttt{gf} \cdot \gamma_1$	-1 if "male"; 1 if "female"	Binary	Sex of the child
$f_1(\texttt{ai})$	Index	Continuous	Asset index of the household
$f_2(\texttt{bord})$	Count	Metric	Birth order within household
$f_3(\texttt{cage})$	Months	Metric	Age of child
$f_4(\texttt{fyvacn})$	Count	Metric	Number of vaccinations completed
$f_5(\texttt{hhs})$	Count	Metric	Household size
$f_6(\texttt{higheduyear})$	Years	Metric	Highest completed year of schooling
$f_7(\texttt{lgdp})$	US$	Continuous	*log*(GDP) of the country
$f_8(\texttt{magebirth})$	Years	Metric	Age of mother at birth
Spatial covariates			
$\texttt{conf25} \cdot \gamma_2$	"yes" $\geq$ 5; "no" $<$ 5	Binary	Indicator whether more than five conflicts have reported in the past within a buffer of 25 *km*
$\texttt{conf50} \cdot \gamma_3$	"yes" $\geq$ 5; "no" $<$ 5	Binary	Indicator whether more than five conflicts have reported in the past within a buffer of 50 *km*,
$\texttt{conf100} \cdot \gamma_4$	"yes" $\geq$ 5; "no" $<$ 5	Binary	Indicator whether more than five conflicts have reported in the past within a buffer of 100 *km*
$\texttt{lc12} \cdot \gamma_5$	Land-cover classification	Categorical	Land-cover classification
$\texttt{soil2} \cdot \gamma_6$	Soil type classification	Categorical	Soil type classification
$f_9(\texttt{altitude})$	Meters	Continuous	Altitude in m above sea level
$f_{10}(\texttt{ldistance})$	Kilometers	Continuous	*log*(1 + distance to closest body of water)
$f_{11}(\texttt{lttcity})$	Hours	Continuous	*log*(1 + travel time to city / 60)
$f_{12}(\texttt{lttmotor})$	Hours	Continuous	*log*(1 + travel time to healthcare facility by motorised vehicle / 60)
$f_{13}(\texttt{lttwalk})$	Hours	Continuous	*log*(1 + travel time to healthcare facility by foot / 60)
$f_{14}(\texttt{minc12})$	Prevalence	Continuous	Malaria incidence
$f_{15}(\texttt{ndvi12})$	Index	Continuous	Normalised difference vegetation index
$f_{16}(\texttt{nl20\_12})$	Index	Metric	Night-time light
$f_{17}(\texttt{pre12})$	Meters	Continuous	Precipitation
$f_{18}(\texttt{survey\_year})$	Years	Metric	Year of the survey
$f_{19}(\texttt{t2m12})$	Kelvin	Continuous	2 m surface temperature
$f_{20}(\texttt{x, y})$	Degree	Continuous	Spatial effect of longitude and latitude coordinates
$f_{21}(\texttt{x, y, gf})$		Continuous	Previous effect varying by gender
$f_{22}(\texttt{x, y, cage})$		Continuous	Spatial-age effect of longitude and latitude coordinates and age of child
$f_{23}(\texttt{x, y, cage, gf})$		Continuous	Previous effect varying by gender
$f_{24}(\texttt{x, y, survey\_year})$		Continuous	Spatio-temporal effect of longitude and latitude coordinates and survey year
$f_{25}(\texttt{x, y, survey\_year, gf})$		Continuous	Previous effect varying by gender

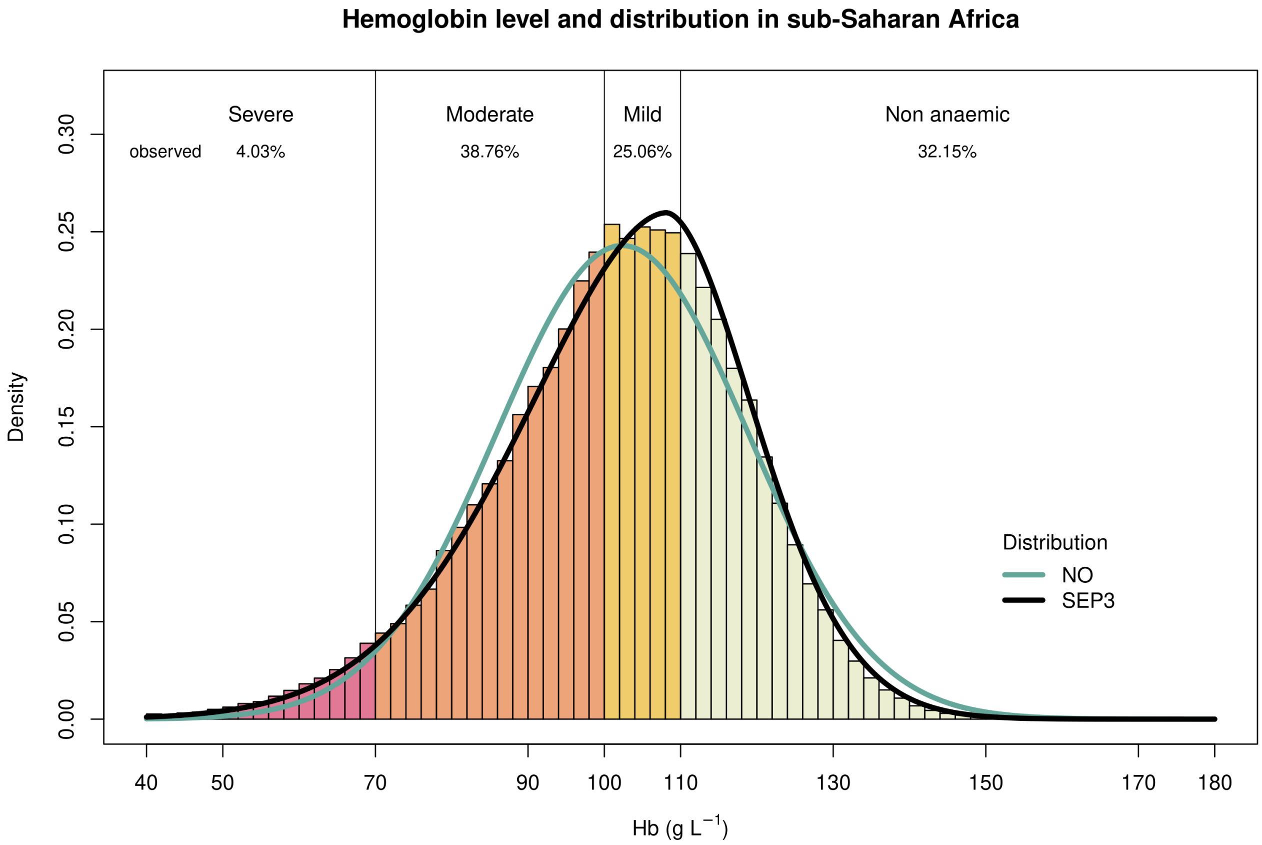

Supplementary Figure 4: Estimated distributions and histogram of the Hb level data among children between 6 and 59 months for sub-Saharan Africa. The shading of the histogram represents the different levels of anemia severity, determined by the specific cutoff values. The estimated densities of a normal distribution (`NO`) and a more flexible exponential generalized Beta type 2 distribution (`SEP3`) are shown. The vertical lines show the cutoffs for being mildly anemic (100 g $L^{-1} \leq$ Hb $<$ 110 g L^{-1}), being moderately anemic (70 g $L^{-1} \leq$ Hb $<$ 100 g L^{-1}), and being severely anemic (Hb $<$ 70 g L^{-1}), also indicated by the different color shadings in the histogram. *Source:* DHS data[81]; calculations by authors.

Supplementary Note 3 Regression diagnostic

Calibration and convergence checks
As pointed out in several applications[41,42] GAMLSS models exhibit good predictive performance – also for locations without observational data – while at the same time covariate effects remain interpretable. To asses the predictive performance, the data set is split into a training data set – containing approximately 80% of the data and is used to derive the final model – and a test data set of approximately 20% (see Supplementary Figure 1 and Supplementary Method 1 for details), which is used for model validation applying the methods described in Supplementary Method 1.

Overall, as shown in Supplementary Figures 5 to 7 by the corresponding PIT histograms the final models are well calibrated. The PIT histograms, show only minor deviations from being uniformly distributed across all bins and thus indicate well calibrated models. Similarly, from the worm plots shown in the same panel, it becomes clear that only minor deviations from the origin exist, and accordingly, conditional on the covariates, the chosen distribution of the final models can be considered to fit the data reasonably well.

Comparing the MSE of each final subregional model, calculated on the test data – i.e., data that were not used for modeling – with the subregional "naive model" – i.e., modeling the Hb level for each subregion based on the parameters of the normal distribution omitting all covariates – using the skill score[66], the improvement ranges from 16.3% to 19.7%, highlighting a substantial improvement in the predictive ability of the final models compared to these "naive models". Similarly, using the same metric for the CRPS[62] the improvement ranges from 9.6% to 11.9%, highlighting this improvement.

In addition, Figure 1 of the manuscript and Supplementary Figures 12 to 15 highlight the high overlap of model-based estimates at different resolutions for the continuous Hb level and the resulting anemia prevalence estimates, underscoring the reliability of these estimates.

Coefficient sampling Further, standard diagnostic checks for MCMC sampling have been used to check whether the generated MCMC samples converged properly. These diagnostic plots are shown in Supplementary Figures 8 to 10, which also highlight the overall good sampling properties of the final model.

Results potential data sparseness issues – leave one administrative region out cross-validation
Even though GAMLSS models exhibit good overall predictive performance – also for locations without observational data – to analyze and remedy potential data sparseness issues we conducted an extensive cross-validation procedure previously described in Section 1. While conducting this cross-validation procedure we did not encounter any big issues caused by data sparseness. This is also depicted in Supplementary Figure 11 where the CRPS skill score, is shown for each fold (i.e., corresponding to the administrative entity that has been omitted in this fold).

The skill score is a metric used to assess the degradation in a model's predictive performance when specific regions are excluded from the training data. It quantifies the relative change (%) in CRPS between two scenarios:

1. **Reference Model (i.e., the final model of step 4)**: This model is trained using the available training data, including the data from the administrative region under evaluation. The CRPS is computed using both training and testing data from that administrative region.

2. **Evaluation Model**: This model is trained excluding the data from the administrative region under evaluation. The CRPS is computed similarly using both training and testing data from that region.

The skill score for a particular region indicates how much the model's predictive performance suffers (negative score) when excluding the data from that region. This gives us a estimate how the data sparsity might effect the forecast. As the reference model also sees part of the evaluation data in the training process it is especially conservative.

Overall the relative change in 71 (or 93.4%) of a total of 76 folds is greater or equal to -5.15%, which implies only a minimal deterioration compared to the corresponding final model. Only in five of a total of 76 folds the relative change was smaller than 5.15%. Thus overall data sparseness issues are of minor importance and lead

only to a slight deterioration of the estimated models. This gives a hint that in areas where the relative change appears big (i.e., Eswatini, Ethiopia, Myanmar, Nepal, and Lesotho) new and more detailed survey data is urgently needed.

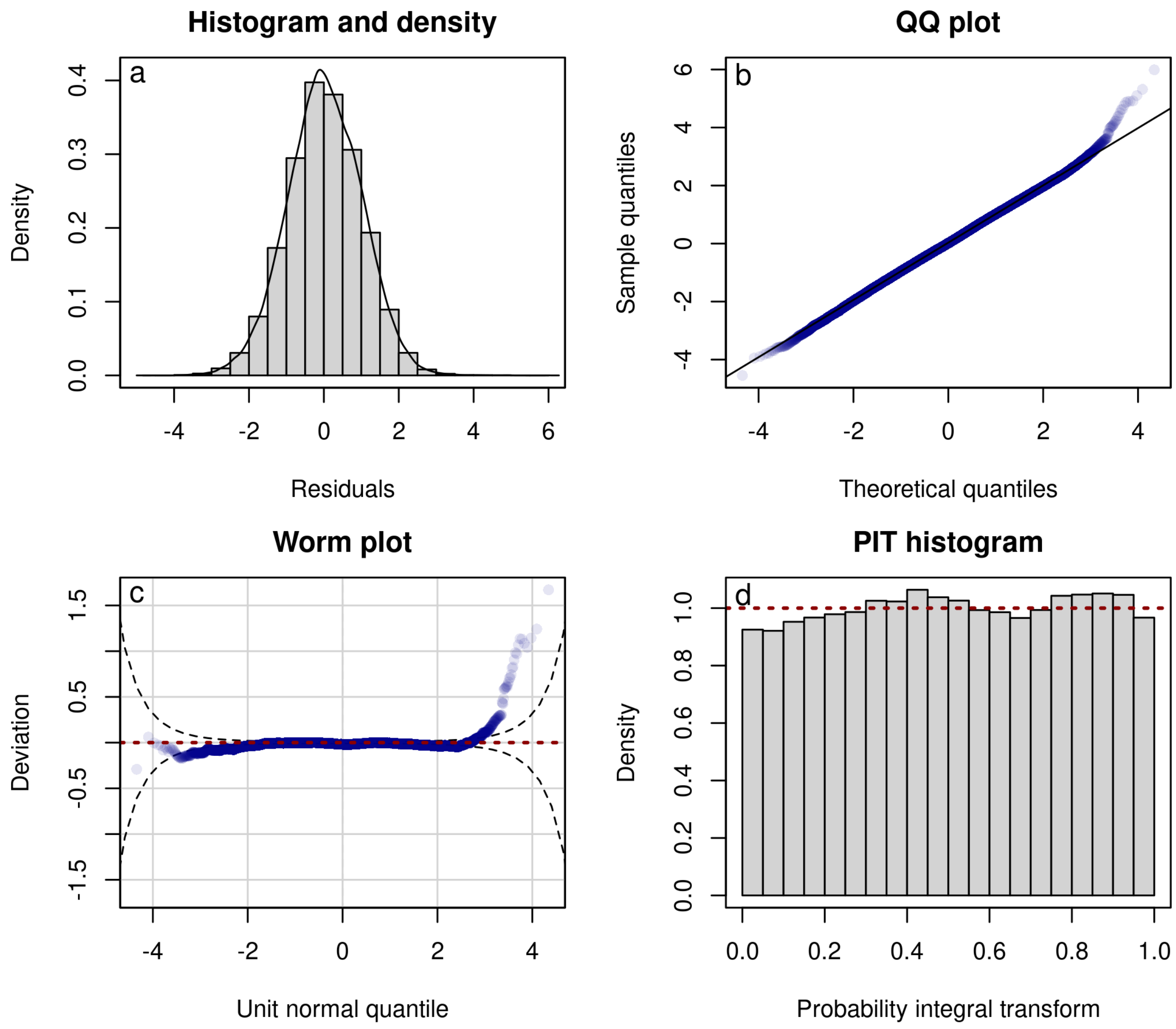

Supplementary Figure 5: **Residual diagnostic of the final model-based on the** SEP3 **distribution for sub-Saharan Africa. (a)** depicts the histogram of the out-of-sample randomized quantile residuals of the Hb level; **(b)** depicts the QQ-plot of the out-of-sample randomized quantile residuals; **(c)** shows the worm plot of the out-of-sample randomized quantile residuals; and **(d)** shows the PIT histogram evaluated using the out-of-sample data.

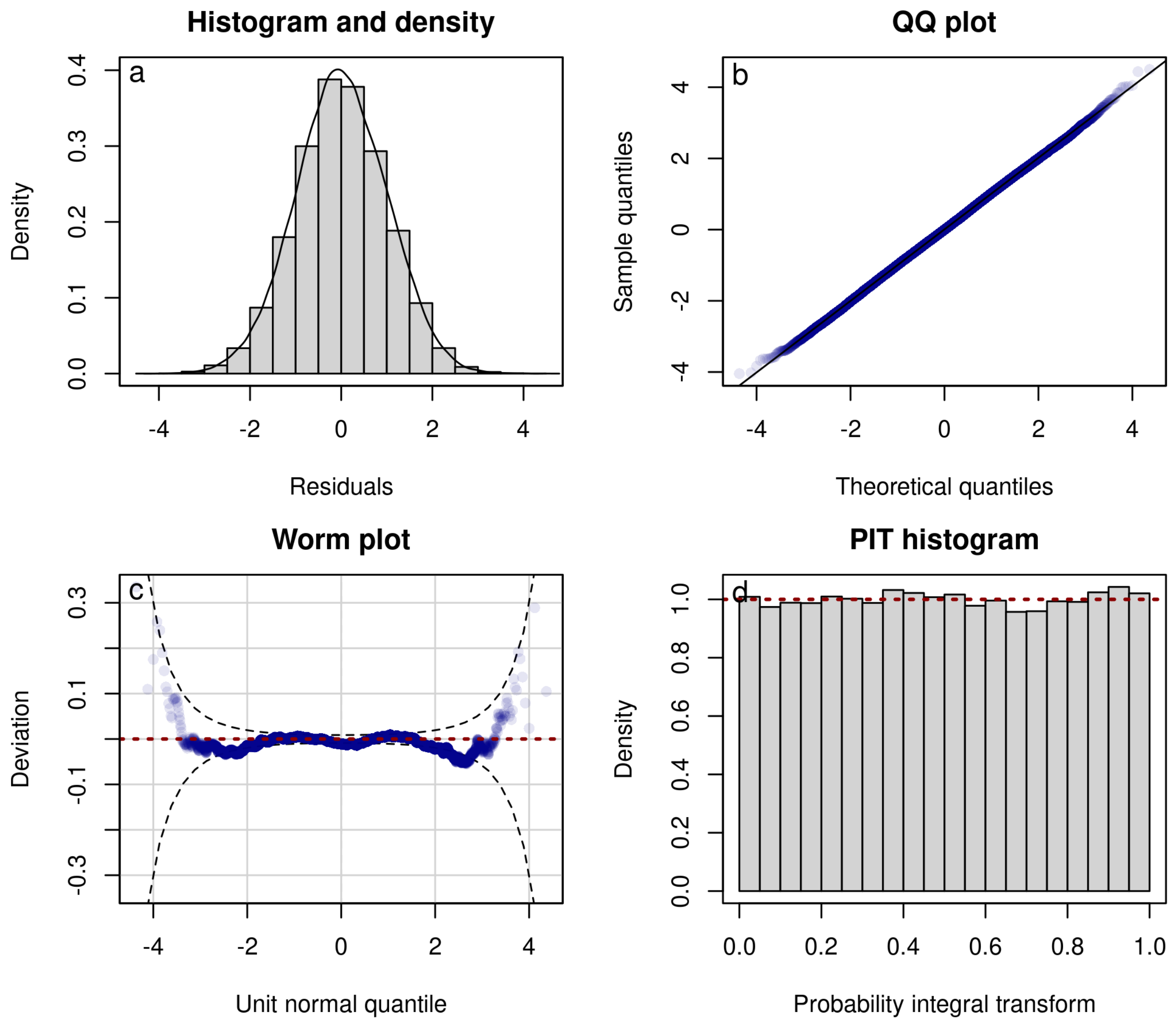

Supplementary Figure 6: Residual diagnostic of the final model-based on the ST3 distribution for South Asia. **(a)** depicts the histogram of the out-of-sample randomized quantile residuals of the Hb level; **(b)** depicts the QQ-plot of the out-of-sample randomized quantile residuals; **(c)** shows the worm plot of the out-of-sample randomized quantile residuals; and **(d)** shows the PIT histogram evaluated using the out-of-sample data.

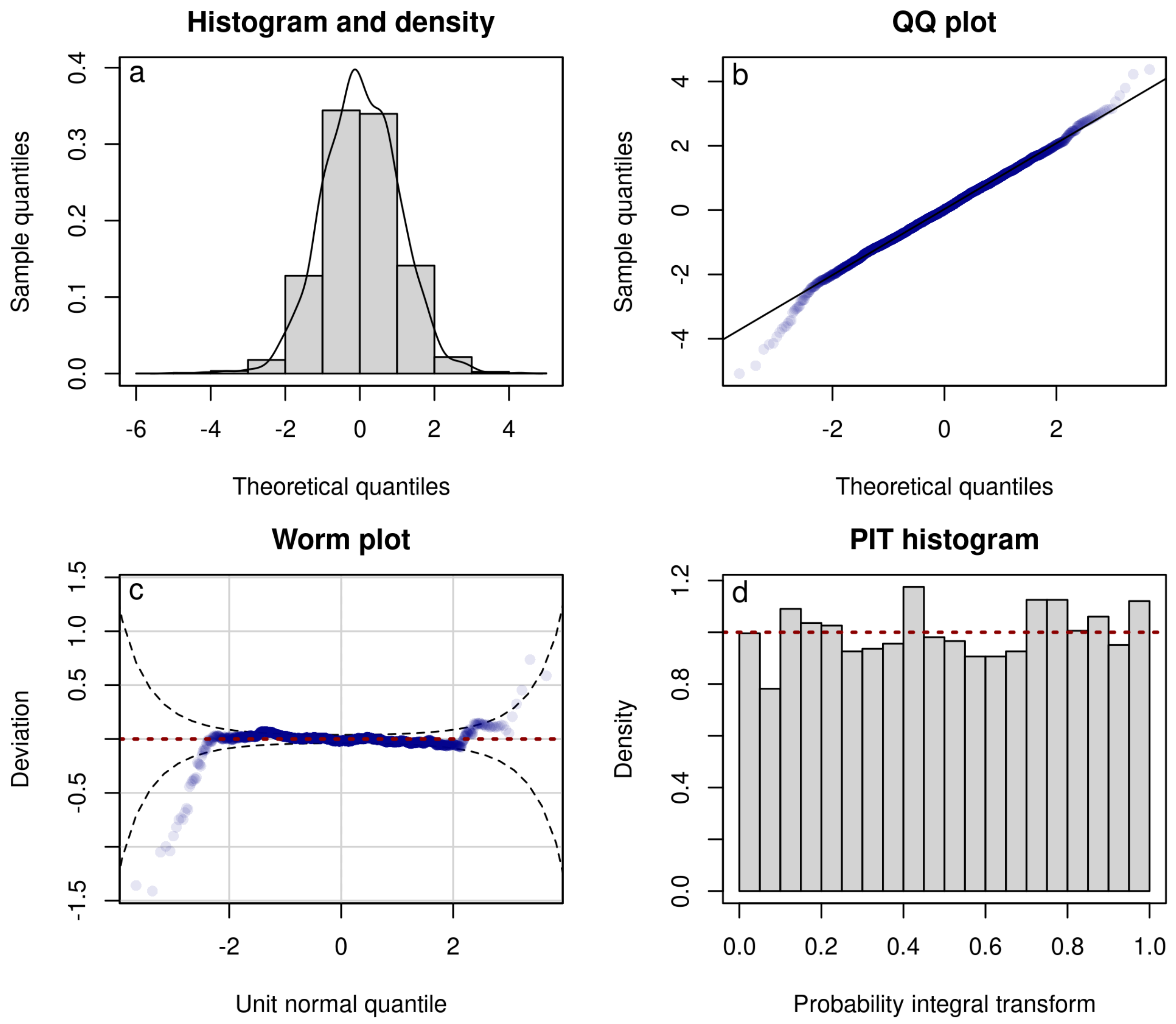

Supplementary Figure 7: Residual diagnostic of the final model-based on the `SEP3` distribution for Madagascar. **(a)** depicts the histogram of the out-of-sample randomized quantile residuals of the Hb level; **(b)** depicts the QQ-plot of the out-of-sample randomized quantile residuals; **(c)** shows the worm plot of the out-of-sample randomized quantile residuals; and **(d)** shows the PIT histogram evaluated using the out-of-sample data.

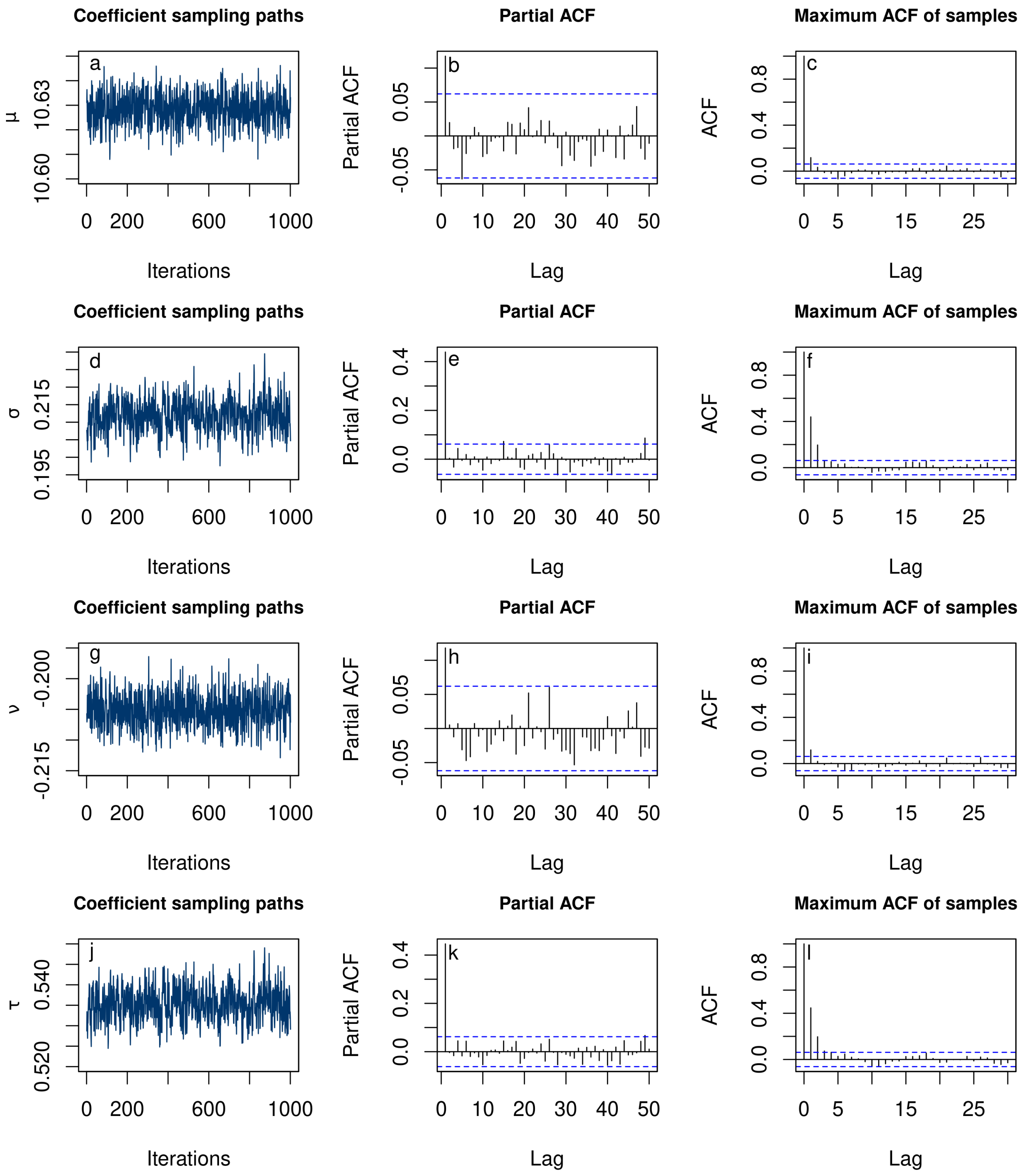

Supplementary Figure 8: **Coefficient sampling paths and autocorrelation function (ACF) plots from the final models for sub-Saharan Africa. a, d, g,** and **j** depict the sampling path of the intercept for the four parameters μ, σ, ν, and τ of the SEP3 distribution used in the final model, respectively. The 1,000 samples are generated from a single Markov chain with 12,000 iterations, a burn in of 2,000 iterations and a thinning parameter of 10; **b, e, h,** and **k** show the corresponding partial ACF; and **c, f, i,** and **l**, show the corresponding maximum ACF values over all model parameters.

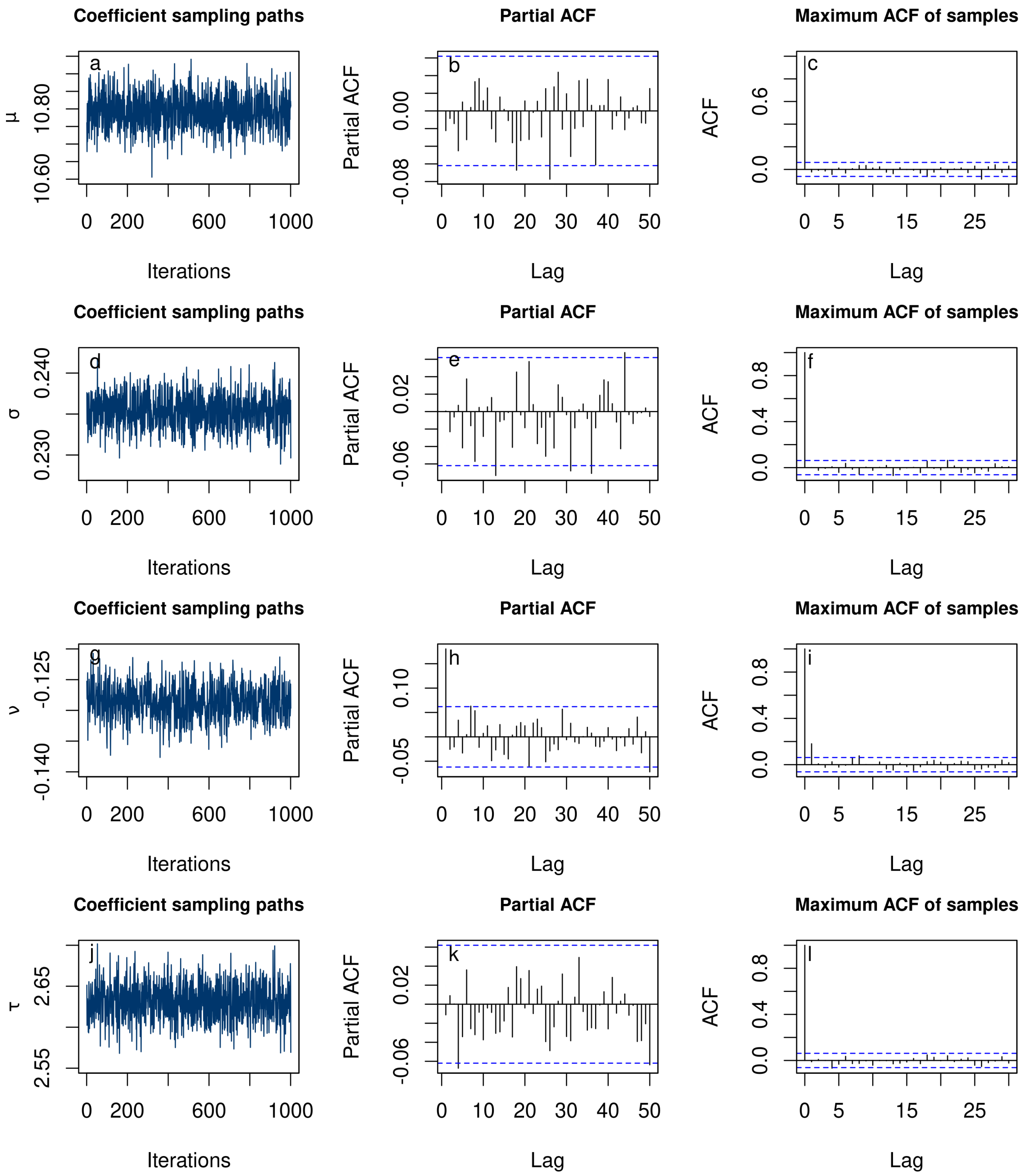

Supplementary Figure 9: **Coefficient sampling paths and autocorrelation function (ACF) plots from the final models for South Asia. a, d, g,** and **j** depict the sampling path of the intercept for the four parameters μ, σ, ν, and τ of the ST3 distribution used in the final model, respectively. The 1,000 samples are generated from a single Markov chain with 12,000 iterations, a burn in of 2,000 iterations and a thinning parameter of 10; **b, e, h,** and **k** show the corresponding partial ACF; and **c, f, i,** and **l**, show the corresponding maximum ACF values over all model parameters.

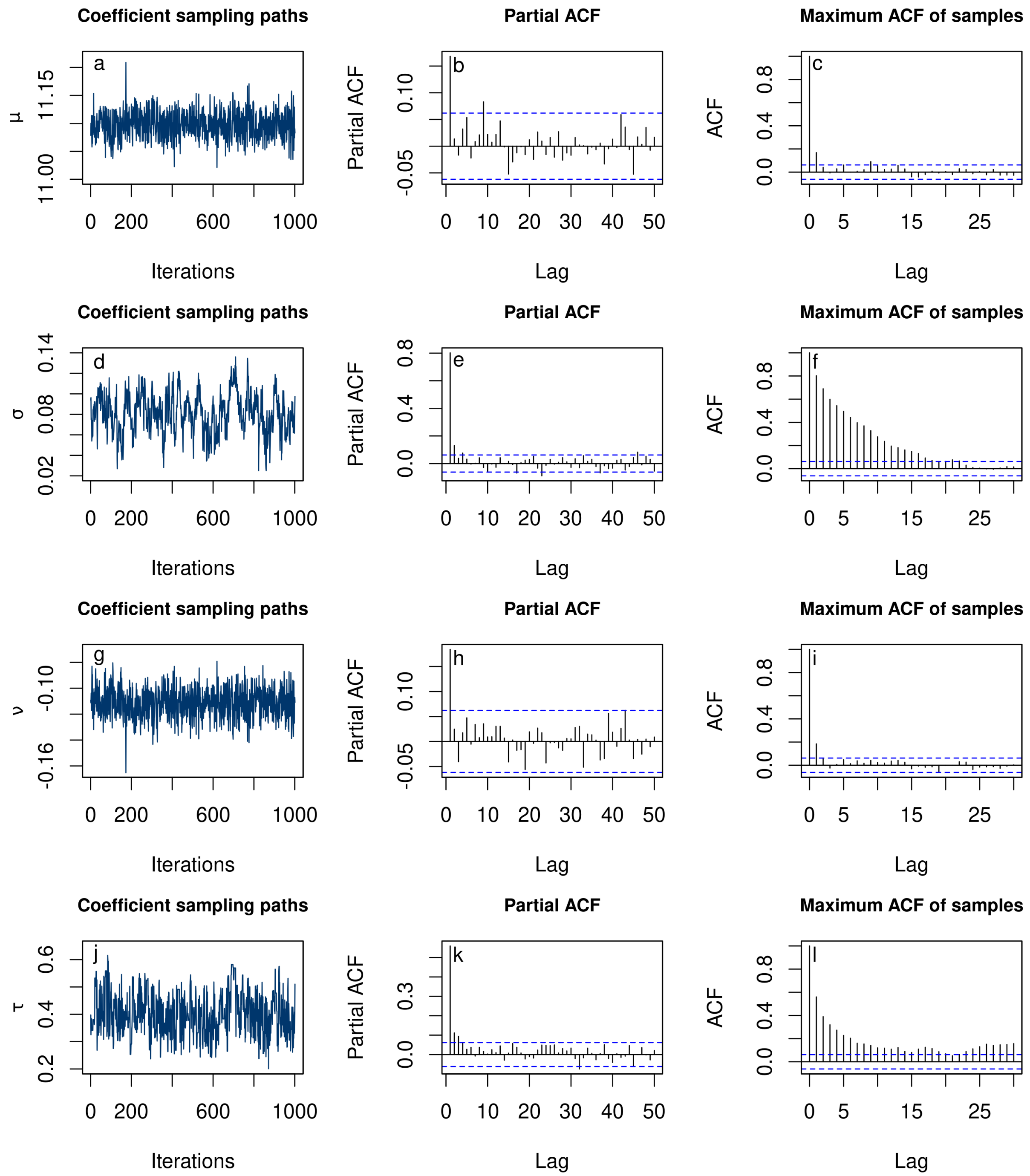

Supplementary Figure 10: **Coefficient sampling paths and autocorrelation function (ACF) plots from the final models for Madagascar. a, d, g,** and **j** depict the sampling path of the intercept for the four parameters μ, σ, ν, and τ of the SEP3 distribution used in the final model, respectively. The 1,000 samples are generated from a single Markov chain with 12,000 iterations, a burn in of 2,000 iterations and a thinning parameter of 10; **b, e, h,** and **k** show the corresponding partial ACF; and **c, f, i,** and **l**, show the corresponding maximum ACF values over all model parameters.

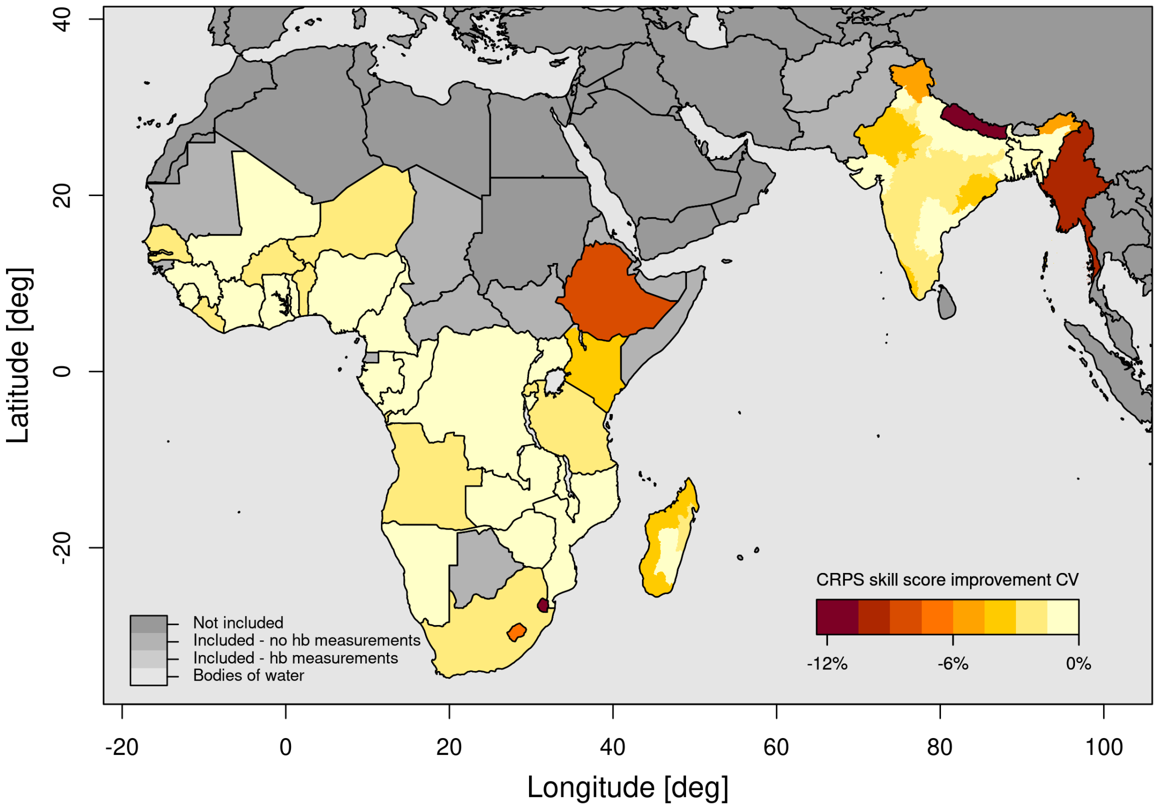

Supplementary Figure 11: Change in the CRPS skill score for all cross-validation folds against the corresponding final models. Note that the negative sign implies a relative increase and thus a worsening of the CRPS (calculated on the hold-out data of a specific fold) compared to the CRPS of the final model that has been calculated on the same hold-out data.

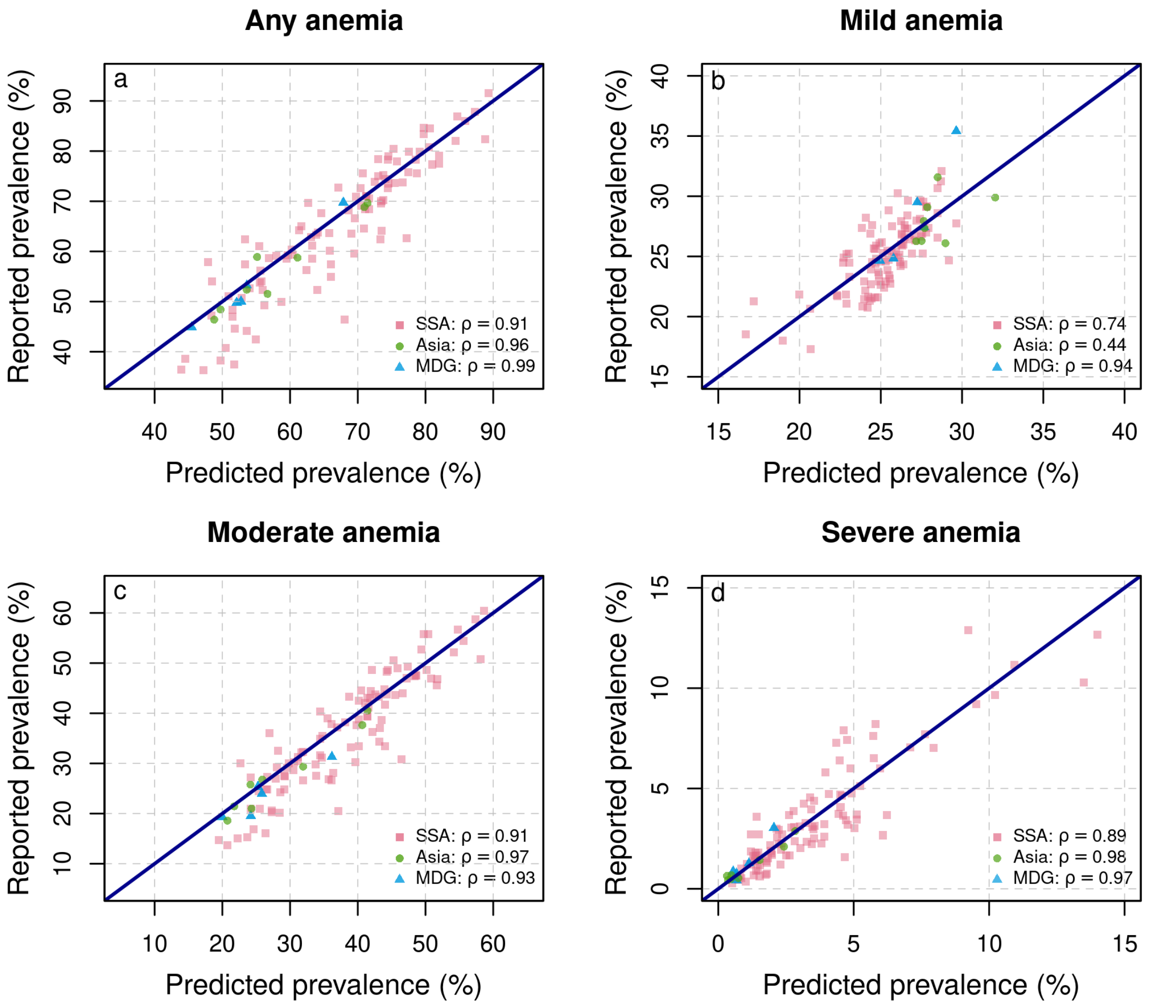

Supplementary Figure 12: **Scatter plot observed anemia prevalence and estimated anemia prevalence at the country level.** **(a)** Scatter plot of the survey-based prevalence of any anemia (i.e., $P(\text{Hb} < 110 \text{ g L}^{-1})$); **(b)** mild anemia (i.e., $P(100 \text{ g L}^{-1} \leq \text{Hb} < 110 \text{ g L}^{-1})$); **(c)** moderate anemia (i.e., $P(70 \text{ g L}^{-1} \leq \text{Hb} < 100 \text{ g L}^{-1})$); and **(d)** and severe anemia (i.e., $P(\text{Hb} < 70 \text{ g L}^{-1})$) among children aged 6 to 59 months reported by DHS and the model-based estimates aggregated to the country level. In addition, the correlation coefficient ρ is reported.

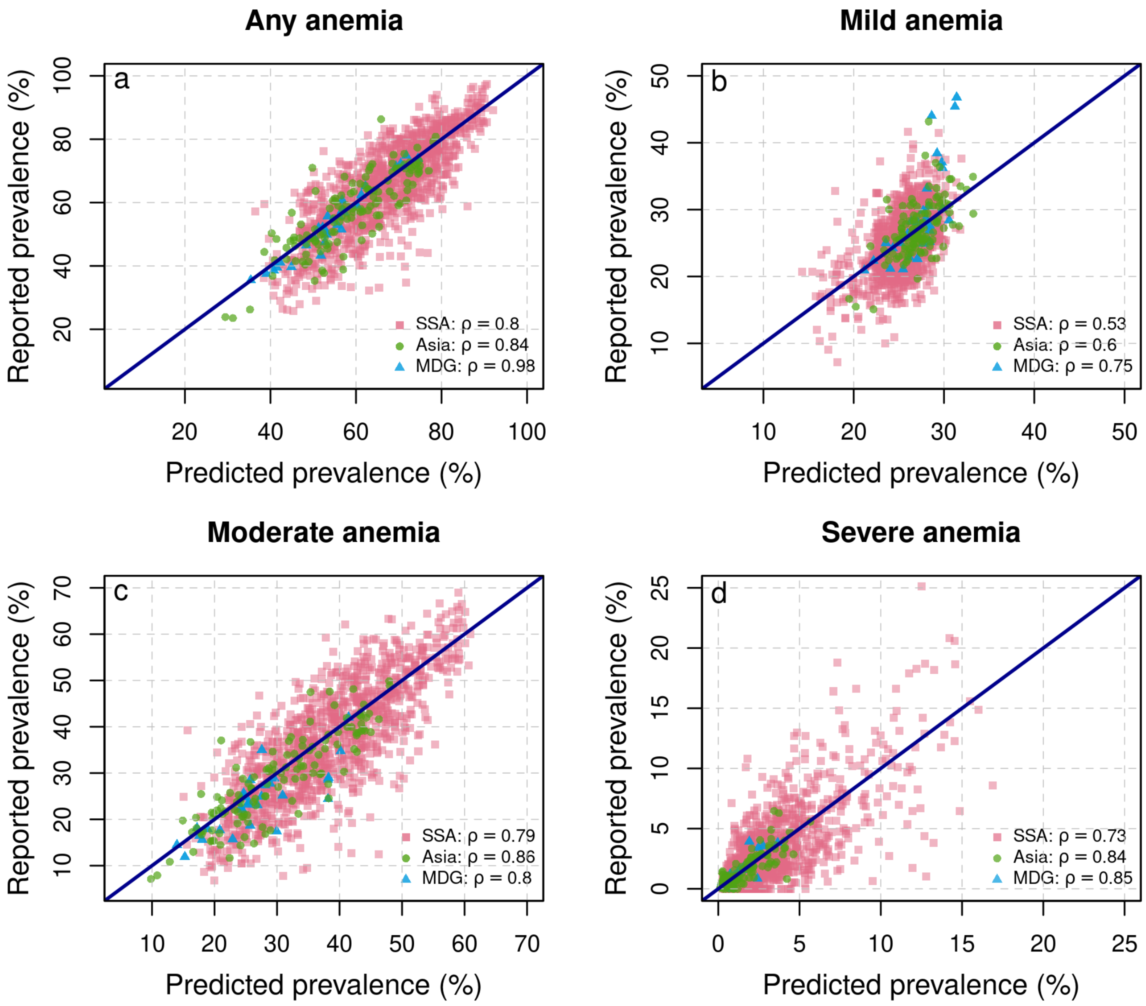

Supplementary Figure 13: **Scatter plot observed anemia prevalence and estimated anemia prevalence at the admin-1 level. (a)** Scatter plot of the survey-based prevalence of any anemia (i.e., $P(\text{Hb} < 110 \text{ g L}^{-1})$); **(b)** mild anemia (i.e., $P(100 \text{ g L}^{-1} \leq \text{Hb} < 110 \text{ g L}^{-1})$); **(c)** moderate anemia (i.e., $P(70 \text{ g L}^{-1} \leq \text{Hb} < 100 \text{ g L}^{-1})$); and **(d)** and severe anemia (i.e., $P(\text{Hb} < 70 \text{ g L}^{-1})$) among children aged 6 to 59 months reported by DHS and the model-based estimates aggregated to admin-1 regions within countries. In addition, the correlation coefficient ρ is reported.

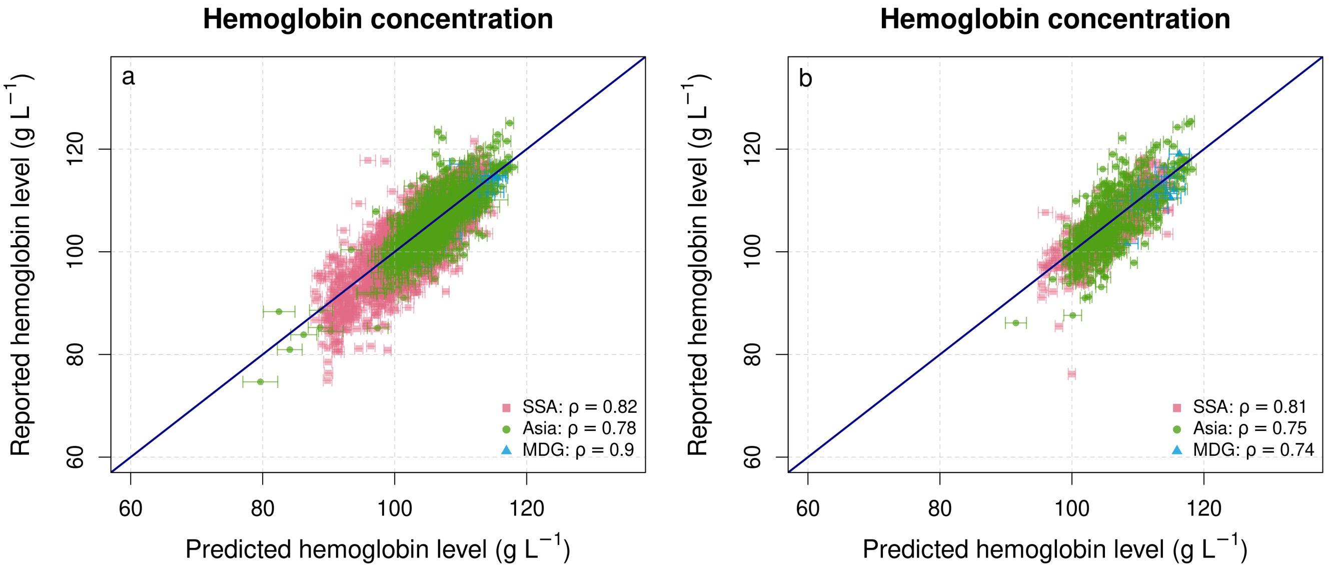

Supplementary Figure 14: **Scatter plot observed Hb concentrations and estimated Hb concentrations at the admin-2 level. (a)** Scatter plot of the survey-based Hb concentrations reported by DHS (calculated using the in sample data) and the corresponding model-based estimates aggregated to admin-2 regions within countries. **(b)** Scatter plot of the survey-based Hb concentrations reported by DHS (calculated using the out-of-sample data) and the corresponding model-based estimates admin-2 regions within countries. In addition, the panel shows 95% credible intervals for each location and the correlation coefficient ρ for each geographic region.

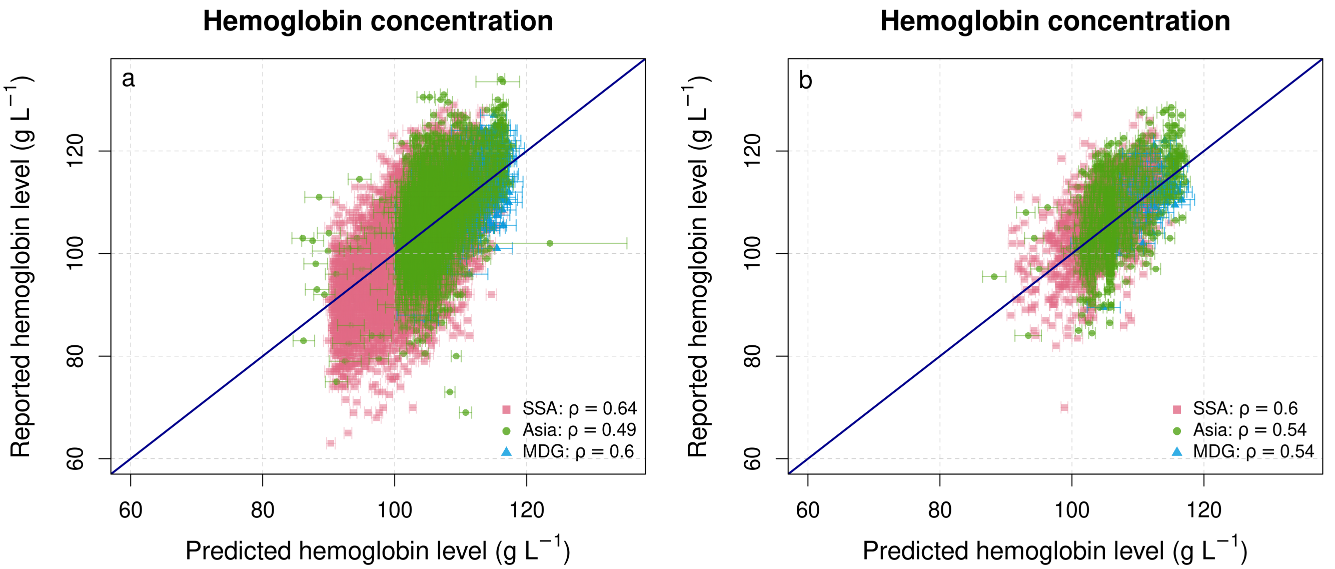

Supplementary Figure 15: **Scatter plot observed Hb concentrations and estimated Hb concentrations at the pixel level. (a)** Scatter plot of the survey-based Hb concentrations reported by DHS (calculated using the in sample data) and the corresponding model-based estimates aggregated to the pixel level. **(b)** Scatter plot of the survey-based Hb concentrations reported by DHS (calculated using the out-of-sample data) and the corresponding model-based estimates aggregated to the pixel level. In addition, the panel shows 95% credible intervals for each location and the correlation coefficient ρ for each geographic region.

Supplementary Table 4: Coverage of the 95% posterior prediction interval (PPI) calculated using the out-of-sample data by region.

Region	Percentage outside PPI (%)	Percentage inside PPI (%)
Sub-Saharan Africa	4.7	95.3
South Asia	5.08	94.92
Madagascar	5.18	94.82

Supplementary Note 4 Software and computational details

Software The results of this paper have been accomplished using custom software, tailored to be used at the high performance (HPC) infrastructure *LEO* of the University of Innsbruck. For that purpose the statistical software R[82] using the following attached R packages has been used: **bamlss**[59,74,77], **backports**[83], **broom**[84], **coda**[85], **codetools**[86], **colorspace**[87,88], **deldir**[89], **dismo**[90], **gamlss.dist**[73], **maps**[91], **mgcv**[69], **nlme**[92,93], **pillar**[94], **raster**[95], **rgeos**[96], **rgdal**[97], **rnaturalearth**[98], **rnaturalearthdata**[99], **rnaturalearthhires**[100], **scales**[101], **scoringRules**[102], **sf**[103], **smoothr**[104], **sp**[105,106]. The custom R-code[82] which was used to perform the statistical analysis is provided in the zip-file labeled *R-scripts_anemia-paper.zip*.

Computational details To illustrate the approach described in detail in Supplementary Method 1 and to make our computational results more transparent the R-script **example.** R illustrates for simulated data the individual steps of the modeling approach. Moreover, all the relevant R-scripts are provided in the zip-file *R-scripts_anemia-paper.zip*. Please note that the code is custom tailored to the HPC infrastructure *LEO* of the University Innsbruck and maybe adaptions to other systems would be required. In addition, note that we do not have permission by DHS to pass on the DHS survey data to other researchers.

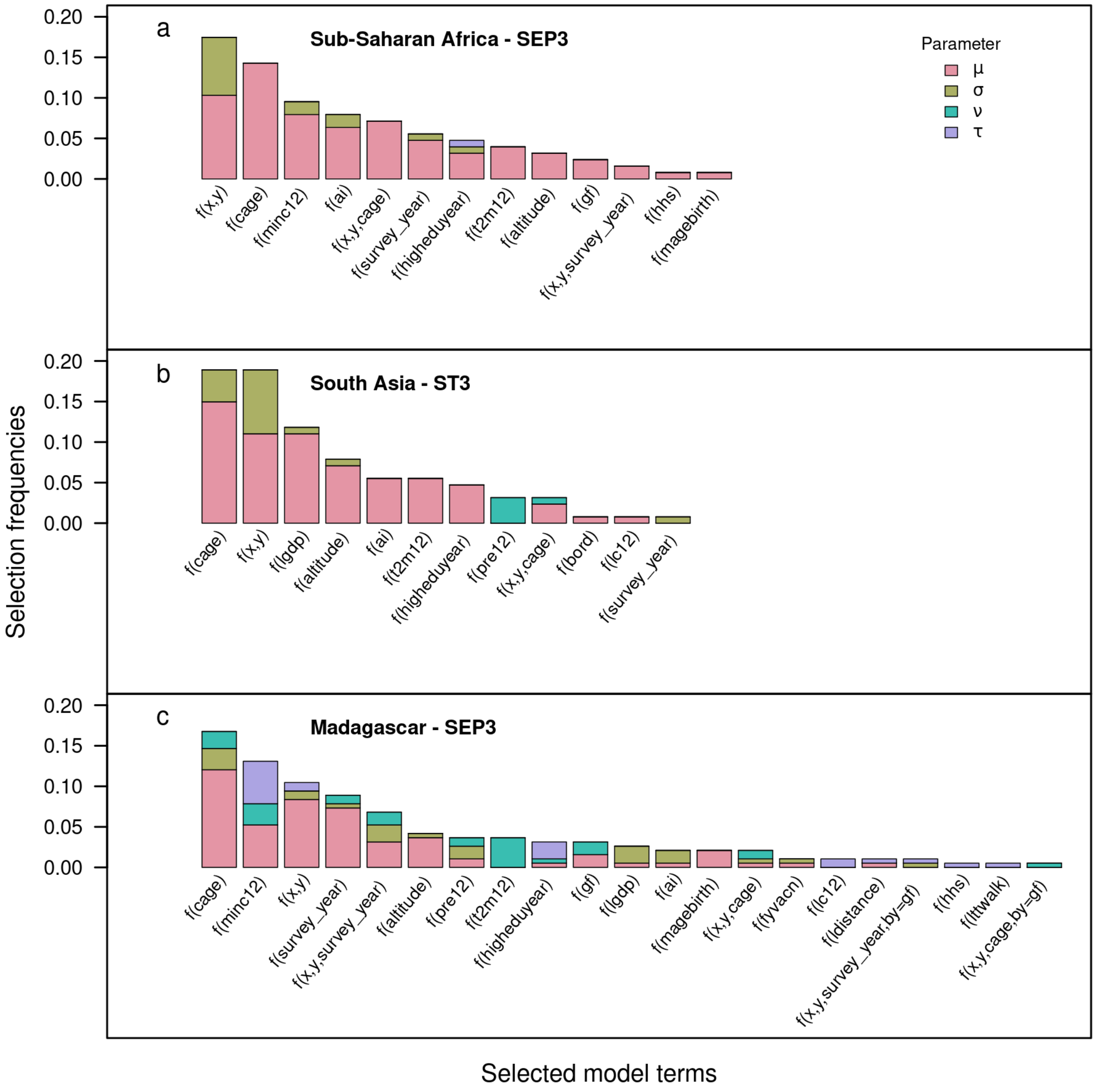

Supplementary Figure 16: **Barplot of the selection frequencies of selected model terms, selected in the selection model for sub-Saharan Africa, South Asia, and Madagascar. (a)** shows the selection frequencies for sub-Saharan Africa; **(b)** shows the selection frequencies for South Asia; and **(c)** shows the selection frequencies for Madagascar. The color shading indicates the distributional parameter (μ, σ, ν, and τ) a specific term was selected. Note that in the context of the GAMLSS framework (i.e., distributional regression), the idea is to characterize all distributional parameters of an arbitrary response distribution using covariates. Here μ corresponds to the location parameter, σ to the scale parameter, ν to the skewness parameter, and τ to the kurtosis parameter. Be aware that depending on the distribution e.g., μ does not necessarily correspond to the mean of the distribution.

Supplementary Table 5: Model-based prevalence and estimated prevalence (by WHO[107]) together with 95% uncertainty intervals of any anemia (i.e., P(Hb $<$ 110g L^{-1})) in children aged 6 – 59 months at the country level for the years 2010, 2019, and 2020.

Country code	Year	Prevalence (%)	Prevalence WHO (%)	Population (in thousands)	Country code	Year	Prevalence (%)	Prevalence WHO (%)	Population (in thousands)
AFG	2010	56.2 [51 - 61.5]	43.7 [34.3 - 54.8]	4592.4	LSO	2010	47.9 [44.4 - 51.4]	48.7 [39.9 - 57.6]	230.6
AFG	2019	58 [52.7 - 63.3]	44.9 [28.1 - 64.4]	5579.3	LSO	2019	53.8 [49.9 - 57.7]	51.4 [32.1 - 69.4]	245.3
AFG	2020	57.8 [52.4 - 63.1]		5688.1	LSO	2020	53.2 [49.2 - 57.2]		245.7
AGO	2010	58.1 [55.7 - 60.5]	61 [52.6 - 69]	3868.5	MDG	2010	53.9 [47.8 - 60]	52.3 [45.9 - 58.6]	3144.9
AGO	2019	63.5 [61 - 66]	62.4 [50 - 72.7]	5071.9	MDG	2019	47.3 [40.2 - 54.8]	49.5 [35.9 - 62.9]	3570.0
AGO	2020	62.7 [60.1 - 65.3]		5192.4	MDG	2020	47.3 [40.2 - 54.8]		3636.8
BDI	2010	55.2 [53.7 - 56.6]	52.4 [44.6 - 60.6]	1544.4	MLI	2010	84.3 [83.1 - 85.5]	81.8 [78.2 - 84.6]	2662.3
BDI	2019	55.2 [53.6 - 56.8]	58 [46.3 - 68.9]	1848.2	MLI	2019	78.1 [76.5 - 79.7]	79 [72.7 - 83]	3384.5
BDI	2020	54.4 [52.8 - 56.1]		1846.0	MLI	2020	77.4 [75.7 - 79]		3465.5
BEN	2010	77.8 [76.5 - 79]	73.2 [66.6 - 78.2]	1426.7	MMR	2010	52.1 [48.2 - 55.9]	50 [35.5 - 64.8]	4142.6
BEN	2019	74.2 [72.7 - 75.6]	71 [62.8 - 76.7]	1817.6	MMR	2019	49.4 [45.9 - 52.8]	49.6 [35.3 - 64.4]	4047.5
BEN	2020	73.3 [71.8 - 74.8]		1859.6	MMR	2020	48.7 [45.2 - 52.1]		4051.5
BFA	2010	87.3 [86.4 - 88.2]	86.6 [83.7 - 88.5]	2612.5	MOZ	2010	71.9 [70.1 - 73.6]	65.1 [57.5 - 71.5]	3602.1
BFA	2019	77.8 [76.4 - 79.1]	76.6 [68.4 - 84.6]	3122.8	MOZ	2019	73.1 [71.1 - 75.1]	68.2 [54.4 - 77.8]	4471.0
BFA	2020	77.1 [75.7 - 78.4]		3153.4	MOZ	2020	72.7 [70.6 - 74.7]		4571.9
BGD	2010	60 [57.8 - 62.2]	46.1 [35.4 - 55.9]	14580.9	MRT	2010	77.3 [75.4 - 79.1]	69.9 [54.5 - 81.1]	508.5
BGD	2019	54.9 [52.8 - 57.1]	43.1 [24.4 - 61.1]	13144.5	MRT	2019	74.6 [72.6 - 76.5]	65.5 [47.5 - 78]	596.9
BGD	2020	54.3 [52.1 - 56.5]		13171.9	MRT	2020	74.1 [72.1 - 76.1]		607.8
BTN	2010	61.7 [58.9 - 64.4]	53.4 [39.4 - 68.2]	60.3	MWI	2010	65.8 [64.2 - 67.4]	64.8 [59.1 - 69.6]	2419.2
BTN	2019	64.1 [61.5 - 66.6]	44.7 [28.4 - 63]	50.1	MWI	2019	65.2 [63.3 - 67]	55.1 [42.8 - 66.2]	2639.2
BTN	2020	64.3 [61.7 - 66.8]		47.9	MWI	2020	64.6 [62.6 - 66.5]		2668.8
BWA	2010	56 [52.9 - 59.1]	37.5 [20.1 - 58.1]	239.0	NAM	2010	57.2 [53.2 - 61.3]	45.6 [32.4 - 58.8]	249.1
BWA	2019	62.8 [59.5 - 66.1]	43.3 [21.7 - 67.2]	264.1	NAM	2019	63.9 [59.6 - 68.2]	46.1 [27.5 - 65.9]	295.5
BWA	2020	62.3 [58.8 - 65.6]		265.6	NAM	2020	63.5 [59.1 - 67.8]		296.9
CAF	2010	58.9 [56.5 - 61.3]	77.7 [66.2 - 85.2]	734.4	NER	2010	76.9 [75.2 - 78.6]	77.8 [72.8 - 81.3]	2973.5
CAF	2019	54.2 [51.6 - 56.8]	73.6 [59.2 - 83.4]	826.1	NER	2019	72.8 [70.8 - 74.7]	72 [59.7 - 79.8]	4081.5
CAF	2020	53.3 [50.6 - 55.9]		843.5	NER	2020	72 [70 - 74]		4233.8
CIV	2010	82.1 [80.8 - 83.4]	74.4 [68 - 79]	3159.6	NGA	2010	77 [75.5 - 78.5]	72.6 [66.3 - 77.6]	25324.5
CIV	2019	74.3 [72.6 - 76]	72.2 [59.3 - 80.8]	3623.6	NGA	2019	70.9 [69.1 - 72.6]	68.9 [61.2 - 75.1]	29950.8
CIV	2020	73.5 [71.7 - 75.2]		3680.3	NGA	2020	70.1 [68.2 - 71.9]		30427.2
CMR	2010	66.9 [65 - 68.9]	64 [56.1 - 70.5]	3024.1	NPL	2010	51.1 [48.5 - 53.7]	47.5 [38.9 - 56.2]	2778.5
CMR	2019	64 [61.9 - 66.2]	59.2 [49.1 - 69.5]	3719.1	NPL	2019	60.2 [58 - 62.4]	44.6 [26.4 - 63.8]	2641.3
CMR	2020	63.2 [60.9 - 65.3]		3802.1	NPL	2020	59.5 [57.2 - 61.7]		2643.8
COD	2010	69.1 [67.1 - 71]	69.3 [61.5 - 75.5]	10884.3	PAK	2010	64.6 [61.7 - 67.5]	59 [49.1 - 67.8]	24384.7
COD	2019	63.5 [61.3 - 65.7]	64.9 [50.3 - 76.6]	14776.1	PAK	2019	60.7 [57.7 - 63.7]	53 [37.6 - 68.4]	26304.4
COD	2020	62.6 [60.4 - 64.8]		15212.3	PAK	2020	61.4 [58.4 - 64.4]		26364.6
COG	2010	66 [64 - 68]	64.9 [57 - 71.1]	640.5	RWA	2010	48.9 [47.5 - 50.4]	40.6 [33.8 - 48.3]	1472.6
COG	2019	66.1 [63.9 - 68.3]	60.8 [45.8 - 73.4]	754.3	RWA	2019	49.5 [47.9 - 51.2]	37.9 [29.2 - 47.9]	1632.0
COG	2020	65.2 [63 - 67.5]		755.8	RWA	2020	48.7 [47 - 50.4]		1659.9
DJI	2010	74.8 [72.6 - 77]	52.8 [34.3 - 72.1]	103.4	SEN	2010	75.2 [73.8 - 76.6]	76.5 [72.7 - 79.6]	1868.4
DJI	2019	73.4 [70.9 - 75.7]	52 [31 - 72.4]	103.3	SEN	2019	70.1 [68.5 - 71.7]	67.9 [58.5 - 74.5]	2231.2
DJI	2020	72.9 [70.4 - 75.3]		103.8	SEN	2020	69.1 [67.3 - 70.7]		2259.5
ERI	2010	56.3 [53.2 - 59.4]	49.9 [32.3 - 68.8]	444.6	SLE	2010	81.2 [80 - 82.3]	77.9 [74.3 - 80.9]	938.0
ERI	2019	53.2 [49.8 - 56.5]	48.8 [29 - 69.7]	433.9	SLE	2019	73.1 [71.6 - 74.7]	73.4 [67.8 - 77.8]	1036.3
ERI	2020	52.6 [49.2 - 56]		433.3	SLE	2020	72.2 [70.7 - 73.8]		1046.6
ETH	2010	62.4 [60 - 64.6]	51.6 [43.3 - 59.7]	13375.8	SOM	2010	78.2 [75.8 - 80.5]	59.3 [48.2 - 69.3]	2062.3
ETH	2019	59.3 [56.7 - 61.8]	52.1 [37.3 - 66.2]	15043.8	SOM	2019	78.2 [75.7 - 80.7]	51.8 [39.7 - 65.8]	2682.6
ETH	2020	58.8 [56.2 - 61.4]		15444.8	SOM	2020	77.9 [75.3 - 80.3]		2761.6
GAB	2010	64.9 [62.8 - 67]	63.2 [52.4 - 71.6]	217.4	SSD	2010	49.1 [46.6 - 51.6]	59.5 [41.4 - 75.5]	1442.0
GAB	2019	69.1 [66.9 - 71.3]	57.9 [42.9 - 71.7]	272.9	SSD	2019	45.9 [43.2 - 48.6]	60.5 [39.5 - 77.4]	1504.4
GAB	2020	68.3 [66 - 70.5]		274.4	SSD	2020	45 [42.3 - 47.8]		1421.9
GHA	2010	77.9 [76.5 - 79.2]	70.5 [64.2 - 75.7]	3384.4	SWZ	2010	50.9 [48.4 - 53.6]	44.1 [32.3 - 57.5]	136.4
GHA	2019	71.4 [69.7 - 73]	59.5 [51.1 - 67]	3860.6	SWZ	2019	57.1 [54.1 - 60]	42.7 [22 - 63.8]	129.0
GHA	2020	70.5 [68.7 - 72.2]		3882.0	SWZ	2020	56.6 [53.5 - 59.6]		127.4
GIN	2010	79.6 [78.4 - 80.8]	76.2 [71 - 80.4]	1538.3	TCD	2010	59.3 [56.5 - 62.1]	74.9 [63.2 - 82.5]	2104.6
GIN	2019	72.6 [71.1 - 74.1]	73.8 [65.9 - 79.5]	1821.8	TCD	2019	55.6 [52.5 - 58.5]	66.3 [59.8 - 72.9]	2713.8
GIN	2020	71.7 [70.1 - 73.3]		1848.9	TCD	2020	54.6 [51.5 - 57.6]		2779.0
GMB	2010	76.5 [75.3 - 77.7]	71 [58.4 - 79.7]	312.3	TGO	2010	73.7 [72.3 - 75]	73.3 [65.3 - 79.4]	945.2
GMB	2019	69.2 [67.8 - 70.7]	52.3 [45.8 - 62.3]	364.2	TGO	2019	69.4 [67.8 - 70.9]	72.4 [63.2 - 78.4]	1102.5
GMB	2020	68.1 [66.6 - 69.6]		366.4	TGO	2020	68.5 [66.8 - 70]		1118.5
GNB	2010	75.4 [73.9 - 76.8]	73.2 [57.9 - 82.8]	234.6	TZA	2010	60 [58.4 - 61.7]	62.2 [56 - 67.6]	7144.0
GNB	2019	70.5 [68.9 - 72.1]	68 [50.1 - 79.9]	262.8	TZA	2019	61.4 [59.6 - 63.2]	56.1 [42 - 68.7]	8959.4
GNB	2020	69.5 [67.8 - 71.2]		263.6	TZA	2020	60.7 [58.8 - 62.6]		9169.5
GNQ	2010	74.8 [73 - 76.5]	68.9 [59.8 - 76.1]	163.8	UGA	2010	63.8 [62.4 - 65.3]	59.5 [52.1 - 66.1]	5505.3
GNQ	2019	68.5 [66.4 - 70.6]	64.3 [48.2 - 76.1]	200.5	UGA	2019	57.9 [56.3 - 59.5]	51.7 [42.9 - 60.5]	6526.2
GNQ	2020	67.7 [65.5 - 69.8]		203.1	UGA	2020	57.1 [55.4 - 58.7]		6674.1
IND	2010	66.7 [65 - 68.5]	59.7 [49.6 - 68.2]	114752.8	ZAF	2010	54.1 [49.9 - 58.4]	35.6 [22.2 - 52]	4856.1
IND	2019	63.7 [61.9 - 65.4]	53.4 [39.8 - 65.4]	106287.5	ZAF	2019	60.9 [56.3 - 65.4]	44.4 [24.2 - 67.3]	5183.1
IND	2020	60.9 [59.1 - 62.8]		105521.4	ZAF	2020	60.4 [55.7 - 65]		5191.0
KEN	2010	62.6 [60.7 - 64.5]	39.9 [30.4 - 49.3]	6101.8	ZMB	2010	59 [57 - 61.1]	54.3 [46.1 - 62.3]	2248.2
KEN	2019	63.2 [61.1 - 65.2]	42.8 [28 - 58.7]	6129.1	ZMB	2019	62.3 [60.2 - 64.4]	55.1 [43.6 - 65.8]	2656.1
KEN	2020	62.5 [60.4 - 64.6]		6169.4	ZMB	2020	61.6 [59.5 - 63.8]		2696.7
LBR	2010	75.1 [73.5 - 76.7]	71.3 [65 - 76]	604.2	ZWE	2010	52.7 [50.7 - 54.7]	49.8 [40.5 - 58.9]	1796.3
LBR	2019	71.3 [69.4 - 73.1]	72.3 [65.6 - 77.3]	658.3	ZWE	2019	56.7 [54.4 - 59]	37.8 [23.4 - 53.1]	2047.7
LBR	2020	70.2 [68.3 - 72.1]		661.7	ZWE	2020	56.1 [53.7 - 58.4]		2049.4

Population data is taken from the United Nations population prospects using the single age both sexes combined estimates. The population at risk is calculated by taking the weighted sum of the single age estimates (in years) and assuming that in the first year the population is uniformly distributed across months. This implies that apart from the first age bin, where the weight is 0.5 all other weights for the age bins are one. Country abbreviations correspond to the country-specific ISO3 country codes. *S*ource:[107–109], and own calculations.

Supplementary Table 6: Model-based prevalence and estimated prevalence (by WHO[107]) together with 95% uncertainty intervals of mild anemia (i.e., P($100 \text{ g L}^{-1} \leq \text{Hb} < 110 \text{ g L}^{-1}$) in children aged 6 – 59 months at the country level for the years 2010, 2019, and 2020.

Country code	Year	Prevalence (%)	Prevalence WHO (%)	Population (in thousands)	Country code	Year	Prevalence (%)	Prevalence WHO (%)	Population (in thousands)
AFG	2010	24.8 [22.7 - 26.9]	23.3 [17.3 - 27.7]	4592.4	LSO	2010	22.3 [21.1 - 23.4]	24 [20.7 - 26.7]	230.6
AFG	2019	25.1 [22.9 - 27.4]	24.4 [15.2 - 29.5]	5579.3	LSO	2019	24.9 [23.7 - 26.1]	24.3 [16.5 - 29.2]	245.3
AFG	2020	25 [22.8 - 27.4]		5688.1	LSO	2020	24.9 [23.6 - 26.2]		245.7
AGO	2010	26.5 [25.7 - 27.3]	27.3 [24.7 - 29.2]	3868.5	MDG	2010	28.3 [25.1 - 31.8]	27 [25.1 - 28.4]	3144.9
AGO	2019	28.4 [27.5 - 29.3]	28.5 [25.5 - 30.3]	5071.9	MDG	2019	24.7 [21.2 - 28.5]	24.5 [18.5 - 28.3]	3570.0
AGO	2020	28.4 [27.5 - 29.4]		5192.4	MDG	2020	24.7 [21.2 - 28.5]		3636.8
BDI	2010	25 [24.5 - 25.5]	25.3 [22.8 - 27.4]	1544.4	MLI	2010	20.6 [19.9 - 21.3]	20.1 [18.4 - 22.1]	2662.3
BDI	2019	26.5 [25.9 - 27.1]	25.9 [22 - 28.6]	1848.2	MLI	2019	25.8 [25.1 - 26.5]	24.1 [21.1 - 26.8]	3384.5
BDI	2020	26.4 [25.8 - 27]		1846.0	MLI	2020	26.2 [25.4 - 26.9]		3465.5
BEN	2010	24.3 [23.8 - 24.9]	25.3 [23 - 27.3]	1426.7	MMR	2010	29.6 [27.7 - 31.5]	25.2 [19.5 - 28.9]	4142.6
BEN	2019	26.7 [26.1 - 27.3]	27 [24.6 - 29]	1817.6	MMR	2019	27.3 [25.6 - 29.1]	26.4 [20.5 - 29.6]	4047.5
BEN	2020	27 [26.4 - 27.6]		1859.6	MMR	2020	27.2 [25.4 - 29.1]		4051.5
BFA	2010	19.1 [18.5 - 19.7]	17.7 [16 - 20.5]	2612.5	MOZ	2010	24.8 [24.1 - 25.5]	25.2 [22.9 - 27.1]	3602.1
BFA	2019	25.9 [25.2 - 26.5]	24.3 [18.5 - 28.2]	3122.8	MOZ	2019	26.7 [25.8 - 27.5]	24.8 [20.8 - 28.1]	4471.0
BFA	2020	26.2 [25.5 - 26.9]		3153.4	MOZ	2020	26.9 [26 - 27.7]		4571.9
BGD	2010	30.9 [30 - 31.8]	25.9 [20.7 - 29.1]	14580.9	MRT	2010	24.6 [23.8 - 25.4]	25 [19.3 - 29.1]	508.5
BGD	2019	29.2 [28.1 - 30.5]	25.1 [15 - 30]	13144.5	MRT	2019	25.8 [25.1 - 26.6]	26.7 [20.5 - 30.2]	596.9
BGD	2020	29.1 [27.9 - 30.6]		13171.9	MRT	2020	25.9 [25.1 - 26.7]		607.8
BTN	2010	25.1 [23.8 - 26.4]	25.4 [20.2 - 29]	60.3	MWI	2010	25.4 [24.8 - 26]	25.1 [23.4 - 26.5]	2419.2
BTN	2019	25.2 [24 - 26.3]	24.9 [16.9 - 29.6]	50.1	MWI	2019	27.6 [26.9 - 28.3]	26.5 [23.1 - 28.9]	2639.2
BTN	2020	25.1 [24.1 - 26.2]		47.9	MWI	2020	27.8 [27 - 28.5]		2668.8
BWA	2010	26.1 [25.1 - 27.1]	22.2 [12.7 - 28.9]	239.0	NAM	2010	26.1 [24.8 - 27.5]	24.2 [19.2 - 28]	249.1
BWA	2019	28.5 [27.3 - 29.7]	24.1 [12.9 - 30.1]	264.1	NAM	2019	28.4 [26.8 - 29.9]	24.9 [16.1 - 29.7]	295.5
BWA	2020	28.6 [27.3 - 29.8]		265.6	NAM	2020	28.4 [26.8 - 29.9]		296.9
CAF	2010	24.7 [23.9 - 25.4]	22.3 [17.5 - 26.8]	734.4	NER	2010	23.4 [22.7 - 24]	24.3 [21.8 - 26.5]	2973.5
CAF	2019	25.3 [24.4 - 26.2]	24.3 [18.6 - 28.8]	826.1	NER	2019	26.1 [25.4 - 26.8]	27 [22.4 - 30.1]	4081.5
CAF	2020	25.2 [24.3 - 26.1]		843.5	NER	2020	26.3 [25.6 - 27.1]		4233.8
CIV	2010	23.5 [22.8 - 24.2]	24.1 [21.5 - 26.3]	3159.6	NGA	2010	24.3 [23.7 - 24.9]	24.8 [22.6 - 26.8]	25324.5
CIV	2019	27.4 [26.7 - 28.1]	25 [20.4 - 28.7]	3623.6	NGA	2019	27.4 [26.8 - 28]	26.8 [24.5 - 28.6]	29950.8
CIV	2020	27.7 [27 - 28.5]		3680.3	NGA	2020	27.6 [27 - 28.2]		30427.2
CMR	2010	25.9 [25.3 - 26.5]	25.9 [23.5 - 27.8]	3024.1	NPL	2010	27 [25.7 - 28.2]	25.6 [22.6 - 27.9]	2778.5
CMR	2019	27.5 [26.7 - 28.2]	26.3 [23.1 - 28.8]	3719.1	NPL	2019	25.7 [24.6 - 26.9]	24.4 [16.6 - 29.4]	2641.3
CMR	2020	27.5 [26.8 - 28.3]		3802.1	NPL	2020	26 [24.9 - 27.3]		2643.8
COD	2010	25.7 [25 - 26.3]	24.6 [22.1 - 26.7]	10884.3	PAK	2010	25.1 [23.9 - 26.2]	24.6 [21.4 - 27.3]	24384.7
COD	2019	27.2 [26.5 - 27.9]	26.2 [21.7 - 29.3]	14776.1	PAK	2019	25.2 [24 - 26.6]	24.2 [17.3 - 28.7]	26304.4
COD	2020	27.2 [26.5 - 28]		15212.3	PAK	2020	25.1 [23.8 - 26.7]		26364.6
COG	2010	27.8 [27.2 - 28.5]	28.4 [26.4 - 29.7]	640.5	RWA	2010	24.2 [23.7 - 24.7]	23.6 [20.5 - 26.2]	1472.6
COG	2019	29.4 [28.6 - 30.2]	28.6 [24.5 - 30.6]	754.3	RWA	2019	25.5 [24.9 - 26.1]	23.3 [17.7 - 27.7]	1632.0
COG	2020	29.5 [28.7 - 30.3]		755.8	RWA	2020	25.4 [24.7 - 26]		1659.9
DJI	2010	21.9 [21.1 - 22.8]	23.8 [16.8 - 28.8]	103.4	SEN	2010	25.8 [25.2 - 26.4]	23.8 [22.2 - 25.4]	1868.4
DJI	2019	23.2 [22.3 - 24.2]	24.7 [16.2 - 29.8]	103.3	SEN	2019	27.8 [27.2 - 28.5]	28.5 [26.6 - 29.9]	2231.2
DJI	2020	23.5 [22.5 - 24.4]		103.8	SEN	2020	28.1 [27.4 - 28.7]		2259.5
ERI	2010	23.2 [22.2 - 24.3]	24.2 [17 - 29.1]	444.6	SLE	2010	24.4 [23.9 - 25]	24.9 [22.7 - 26.9]	938.0
ERI	2019	23.6 [22.3 - 24.8]	24.7 [15.7 - 29.9]	433.9	SLE	2019	28.3 [27.8 - 28.9]	27 [24.7 - 28.7]	1036.3
ERI	2020	23.5 [22.3 - 24.8]		433.3	SLE	2020	28.6 [28 - 29.2]		1046.6
ETH	2010	22.7 [21.9 - 23.4]	21.5 [18.7 - 24.3]	13375.8	SOM	2010	21.1 [20.1 - 22]	22.8 [18.8 - 26]	2062.3
ETH	2019	23.7 [22.8 - 24.6]	23 [17 - 27.4]	15043.8	SOM	2019	21.9 [20.8 - 22.9]	23.4 [19 - 27.1]	2682.6
ETH	2020	23.7 [22.8 - 24.6]		15444.8	SOM	2020	22.1 [21 - 23.2]		2761.6
GAB	2010	29.3 [28.6 - 30.1]	27 [23.7 - 29.2]	217.4	SSD	2010	23.6 [22.7 - 24.5]	24.5 [18.1 - 28.8]	1442.0
GAB	2019	30.1 [29.3 - 30.9]	28.2 [23.3 - 30.5]	272.9	SSD	2019	23.7 [22.6 - 24.7]	25 [17.3 - 29.7]	1504.4
GAB	2020	30.3 [29.4 - 31.1]		274.4	SSD	2020	23.5 [22.4 - 24.5]		1421.9
GHA	2010	24.5 [23.9 - 25.1]	25.2 [22.7 - 27.3]	3384.4	SWZ	2010	23.9 [23.1 - 24.8]	23.2 [18.3 - 27.4]	136.4
GHA	2019	28 [27.4 - 28.7]	28.2 [26.4 - 29.6]	3860.6	SWZ	2019	26.5 [25.4 - 27.5]	23.5 [13 - 29.9]	129.0
GHA	2020	28.3 [27.6 - 29]		3882.0	SWZ	2020	26.5 [25.5 - 27.5]		127.4
GIN	2010	24 [23.5 - 24.6]	24 [21.7 - 26.2]	1538.3	TCD	2010	25.1 [24.3 - 26]	23.9 [19.5 - 27.7]	2104.6
GIN	2019	27.7 [27.1 - 28.3]	26.8 [23.2 - 29.2]	1821.8	TCD	2019	25.9 [24.9 - 26.9]	26.7 [25 - 28.2]	2713.8
GIN	2020	28 [27.4 - 28.6]		1848.9	TCD	2020	25.9 [24.8 - 26.9]		2779.0
GMB	2010	25.3 [24.8 - 25.9]	24.5 [21.2 - 27.6]	312.3	TGO	2010	25.4 [24.9 - 26]	25.4 [22.2 - 28.2]	945.2
GMB	2019	28.6 [28 - 29.2]	28.4 [26.4 - 29.7]	364.2	TGO	2019	27.9 [27.3 - 28.5]	26.7 [23.7 - 29.1]	1102.5
GMB	2020	28.9 [28.2 - 29.5]		366.4	TGO	2020	28.1 [27.5 - 28.7]		1118.5
GNB	2010	27 [26.4 - 27.6]	24.5 [19 - 28.7]	234.6	TZA	2010	26.9 [26.3 - 27.6]	26.8 [25.3 - 28.1]	7144.0
GNB	2019	29 [28.3 - 29.6]	26.4 [20.4 - 30]	262.8	TZA	2019	28.6 [27.8 - 29.3]	26.7 [22.2 - 29.4]	8959.4
GNB	2020	29.2 [28.5 - 29.9]		263.6	TZA	2020	28.6 [27.9 - 29.4]		9169.5
GNQ	2010	26.7 [26 - 27.4]	25.7 [22.4 - 28.1]	163.8	UGA	2010	24.1 [23.6 - 24.6]	23.2 [21 - 25.3]	5505.3
GNQ	2019	28.8 [28 - 29.5]	27.5 [22.6 - 30.3]	200.5	UGA	2019	25.3 [24.7 - 25.9]	25.2 [22.2 - 27.6]	6526.2
GNQ	2020	28.9 [28.2 - 29.7]		203.1	UGA	2020	25.3 [24.7 - 25.9]		6674.1
IND	2010	26.6 [25.6 - 27.4]	26.1 [23.3 - 28.2]	114752.8	ZAF	2010	24.4 [23 - 25.8]	19.4 [12.6 - 25.5]	4856.1
IND	2019	27.6 [26.9 - 28.5]	25.7 [20.8 - 29]	106287.5	ZAF	2019	26.8 [25.3 - 28.4]	22.7 [14 - 28.8]	5183.1
IND	2020	27.4 [26.4 - 28.7]		105521.4	ZAF	2020	26.9 [25.4 - 28.5]		5191.0
KEN	2010	25.1 [24.5 - 25.7]	20.8 [15.5 - 25.1]	6101.8	ZMB	2010	26.2 [25.5 - 27]	25.6 [22.5 - 28.1]	2248.2
KEN	2019	26.1 [25.4 - 26.8]	22.2 [15.3 - 27.9]	6129.1	ZMB	2019	28.3 [27.5 - 29]	27.2 [23.9 - 29.3]	2656.1
KEN	2020	26.2 [25.5 - 27]		6169.4	ZMB	2020	28.3 [27.6 - 29.1]		2696.7
LBR	2010	26.5 [25.9 - 27]	27.2 [25.4 - 28.8]	604.2	ZWE	2010	25.4 [24.7 - 26.1]	25.9 [23 - 28.1]	1796.3
LBR	2019	28.4 [27.8 - 29]	26.9 [24.6 - 28.5]	658.3	ZWE	2019	28.1 [27.2 - 29]	23.1 [15.1 - 28.7]	2047.7
LBR	2020	28.6 [28 - 29.2]		661.7	ZWE	2020	28.1 [27.2 - 29]		2049.4

Population data is taken from the United Nations population prospects using the single age both sexes combined estimates. The population at risk is calculated by taking the weighted sum of the single age estimates (in years) and assuming that in the first year the population is uniformly distributed across months. This implies that apart from the first age bin, where the weight is 0.5 all other weights for the age bins are one. Country abbreviations correspond to the country-specific ISO3 country codes. *S*ource:[107–109], and own calculations.

Supplementary Table 7: Model-based prevalence and estimated prevalence (by WHO[107]) together with 95% uncertainty intervals of moderate anemia (i.e., $P(70 \text{ g L}^{-1} \leq \text{Hb} < 100\text{g L}^{-1})$ in children aged 6 – 59 months at the country level for the years 2010, 2019, and 2020.

Country code	Year	Prevalence (%)	Prevalence WHO (%)	Population (in thousands)	Country code	Year	Prevalence (%)	Prevalence WHO (%)	Population (in thousands)
AFG	2010	29.5 [25.5 - 33.7]	18.6 [11.1 - 28.1]	4592.4	LSO	2010	24.2 [21.9 - 26.5]	23.4 [17 - 30.2]	230.6
AFG	2019	30.9 [26.7 - 35.2]	18.9 [8.4 - 35.7]	5579.3	LSO	2019	27.4 [24.8 - 30.2]	25.6 [12.1 - 39.7]	245.3
AFG	2020	30.7 [26.5 - 35]		5688.1	LSO	2020	27 [24.3 - 29.8]		245.7
AGO	2010	30.1 [28.3 - 31.9]	31.9 [24.7 - 38.9]	3868.5	MDG	2010	25 [20.3 - 30]	24.4 [19.2 - 29.6]	3144.9
AGO	2019	33.6 [31.5 - 35.6]	32.2 [20.5 - 41.8]	5071.9	MDG	2019	21.9 [16.6 - 27.6]	23.4 [13.8 - 34.1]	3570.0
AGO	2020	32.8 [30.8 - 34.9]		5192.4	MDG	2020	21.9 [16.6 - 27.6]		3636.8
BDI	2010	28.5 [27.5 - 29.6]	25 [18.8 - 32]	1544.4	MLI	2010	54.2 [52.9 - 55.5]	52 [48.8 - 54.8]	2662.3
BDI	2019	27.6 [26.5 - 28.8]	29.4 [19.6 - 39]	1848.2	MLI	2019	47.8 [46.2 - 49.5]	48.6 [42.5 - 52.6]	3384.5
BDI	2020	27 [25.8 - 28.2]		1846.0	MLI	2020	47.1 [45.4 - 48.7]		3465.5
BEN	2010	47.7 [46.5 - 49]	43.2 [37.4 - 48]	1426.7	MMR	2010	22.1 [19.4 - 24.9]	23.4 [13 - 35.6]	4142.6
BEN	2019	43.9 [42.5 - 45.2]	40.5 [33.2 - 45.9]	1817.6	MMR	2019	21.5 [19 - 24]	22.1 [11.5 - 34.7]	4047.5
BEN	2020	43 [41.5 - 44.4]		1859.6	MMR	2020	21 [18.4 - 23.6]		4051.5
BFA	2010	57.5 [56.5 - 58.5]	56.8 [53.6 - 58.9]	2612.5	MOZ	2010	42.5 [41 - 44.2]	36.4 [30.1 - 41.9]	3602.1
BFA	2019	47.7 [46.4 - 49.1]	46.6 [39.2 - 54.9]	3122.8	MOZ	2019	43 [41.2 - 44.9]	39.2 [27.7 - 47.5]	4471.0
BFA	2020	47 [45.6 - 48.4]		3153.4	MOZ	2020	42.5 [40.6 - 44.5]		4571.9
BGD	2010	28.4 [26.7 - 30.2]	19.4 [11.6 - 27.3]	14580.9	MRT	2010	47.3 [45.5 - 49.2]	40.5 [27.8 - 51.1]	508.5
BGD	2019	25.2 [23.5 - 26.9]	17.4 [7.2 - 31.6]	13144.5	MRT	2019	44.5 [42.7 - 46.4]	35.9 [20.7 - 47.5]	596.9
BGD	2020	24.6 [22.8 - 26.4]		13171.9	MRT	2020	44.1 [42.2 - 46]		607.8
BTN	2010	34 [31.5 - 36.5]	26.1 [15.5 - 38.3]	60.3	MWI	2010	37.1 [35.8 - 38.5]	36.2 [31.6 - 40.2]	2419.2
BTN	2019	36.1 [33.7 - 38.4]	18.9 [8.4 - 33.8]	50.1	MWI	2019	35.6 [34 - 37.1]	27.2 [17.3 - 36.5]	2639.2
BTN	2020	36.3 [33.9 - 38.6]		47.9	MWI	2020	35 [33.4 - 36.6]		2668.8
BWA	2010	28.6 [26.4 - 30.9]	14.7 [5.5 - 30.5]	239.0	NAM	2010	29.6 [26.7 - 32.7]	20.6 [11.3 - 30.7]	249.1
BWA	2019	33 [30.3 - 35.7]	18.4 [6.7 - 37.4]	264.1	NAM	2019	34 [30.6 - 37.6]	20.3 [8.5 - 36.3]	295.5
BWA	2020	32.4 [29.7 - 35.2]		265.6	NAM	2020	33.6 [30.1 - 37.2]		296.9
CAF	2010	31.9 [30.1 - 33.6]	47.9 [37.9 - 55.6]	734.4	NER	2010	46.9 [45.4 - 48.5]	47.5 [43.2 - 51.1]	2973.5
CAF	2019	27.5 [25.7 - 29.4]	43.6 [30.5 - 53.3]	826.1	NER	2019	42.8 [41.1 - 44.5]	41.5 [30.4 - 49.3]	4081.5
CAF	2020	26.8 [24.9 - 28.7]		843.5	NER	2020	42.1 [40.3 - 43.8]		4233.8
CIV	2010	52.2 [50.7 - 53.5]	44.7 [39.4 - 48.9]	3159.6	NGA	2010	46.9 [45.4 - 48.3]	42.8 [37.3 - 47.4]	25324.5
CIV	2019	43.8 [42.1 - 45.4]	42.4 [31 - 50.5]	3623.6	NGA	2019	40.6 [39 - 42.1]	38.8 [31.9 - 44.4]	29950.8
CIV	2020	42.9 [41.2 - 44.5]		3680.3	NGA	2020	39.7 [38.1 - 41.3]		30427.2
CMR	2010	37.9 [36.3 - 39.5]	35.4 [28.9 - 40.8]	3024.1	NPL	2010	23.5 [21.5 - 25.4]	21.3 [14.9 - 28.2]	2778.5
CMR	2019	34.6 [32.9 - 36.4]	30.5 [22 - 39.5]	3719.1	NPL	2019	32.5 [30.6 - 34.5]	19.7 [7.5 - 34.5]	2641.3
CMR	2020	33.8 [32.1 - 35.6]		3802.1	NPL	2020	31.6 [29.7 - 33.6]		2643.8
COD	2010	39.7 [38.1 - 41.3]	40.4 [34.3 - 45.6]	10884.3	PAK	2010	36.2 [33.8 - 38.7]	30.5 [21.9 - 38.5]	24384.7
COD	2019	34.3 [32.6 - 36]	35.5 [22.7 - 46.5]	14776.1	PAK	2019	32.8 [30.4 - 35.3]	25.7 [13.8 - 39.2]	26304.4
COD	2020	33.5 [31.7 - 35.2]		15212.3	PAK	2020	33.5 [31 - 36]		26364.6
COG	2010	36 [34.4 - 37.6]	34.8 [28 - 40.4]	640.5	RWA	2010	23.8 [22.8 - 24.8]	16.4 [11.7 - 22.4]	1472.6
COG	2019	35.2 [33.4 - 37]	30.8 [17.1 - 42.3]	754.3	RWA	2019	23.3 [22.2 - 24.5]	14.2 [8.6 - 22]	1632.0
COG	2020	34.3 [32.5 - 36.2]		755.8	RWA	2020	22.7 [21.6 - 23.8]		1659.9
DJI	2010	45.4 [43.5 - 47.3]	26.6 [13.1 - 42.8]	103.4	SEN	2010	45.1 [43.8 - 46.5]	46.4 [42.9 - 49.3]	1868.4
DJI	2019	44.3 [42.1 - 46.4]	25.5 [10.8 - 42.4]	103.3	SEN	2019	39.8 [38.3 - 41.3]	37.3 [28.7 - 43.4]	2231.2
DJI	2020	43.8 [41.7 - 46]		103.8	SEN	2020	38.7 [37.2 - 40.3]		2259.5
ERI	2010	30.6 [28.3 - 33]	23.9 [11.2 - 39.1]	444.6	SLE	2010	50.8 [49.6 - 52.1]	47.4 [44 - 50.4]	938.0
ERI	2019	27.9 [25.4 - 30.4]	22.7 [8.9 - 39.7]	433.9	SLE	2019	42 [40.6 - 43.5]	42.7 [37.3 - 47]	1036.3
ERI	2020	27.5 [25 - 30]		433.3	SLE	2020	41.1 [39.7 - 42.6]		1046.6
ETH	2010	35.1 [33.4 - 36.9]	26.8 [20.5 - 33.4]	13375.8	SOM	2010	47.7 [45.6 - 49.8]	32.5 [23.9 - 40.6]	2062.3
ETH	2019	32.5 [30.6 - 34.4]	26.3 [15.6 - 37.8]	15043.8	SOM	2019	48 [45.7 - 50.3]	25.8 [16.8 - 37.4]	2682.6
ETH	2020	32.1 [30.1 - 34]		15444.8	SOM	2020	47.7 [45.4 - 50.1]		2761.6
GAB	2010	34.1 [32.5 - 35.9]	33.9 [25.3 - 41.2]	217.4	SSD	2010	24.3 [22.6 - 26.1]	31.9 [17.7 - 45.5]	1442.0
GAB	2019	37.4 [35.5 - 39.4]	28.4 [15.2 - 40.8]	272.9	SSD	2019	21.6 [19.8 - 23.3]	32.5 [16 - 47.1]	1504.4
GAB	2020	36.5 [34.6 - 38.5]		274.4	SSD	2020	20.9 [19.1 - 22.7]		1421.9
GHA	2010	48 [46.7 - 49.4]	41.2 [35.9 - 45.8]	3384.4	SWZ	2010	25.7 [23.9 - 27.6]	20 [12 - 29.9]	136.4
GHA	2019	40.9 [39.3 - 42.4]	29.6 [22.2 - 36.5]	3860.6	SWZ	2019	29.3 [27.1 - 31.5]	18.4 [7 - 35]	129.0
GHA	2020	40 [38.4 - 41.6]		3882.0	SWZ	2020	28.8 [26.6 - 31.1]		127.4
GIN	2010	49.5 [48.3 - 50.8]	45.9 [40.9 - 50.1]	1538.3	TCD	2010	31.9 [29.8 - 34.1]	44.8 [34.2 - 52.5]	2104.6
GIN	2019	42 [40.6 - 43.4]	43.1 [35.7 - 49]	1821.8	TCD	2019	28.3 [26.1 - 30.5]	35.8 [29.5 - 42.2]	2713.8
GIN	2020	41 [39.6 - 42.5]		1848.9	TCD	2020	27.5 [25.3 - 29.7]		2779.0
GMB	2010	46.4 [45.2 - 47.6]	41.2 [29.7 - 49.3]	312.3	TGO	2010	43.9 [42.6 - 45.1]	43.4 [36.3 - 49.2]	945.2
GMB	2019	38.6 [37.3 - 39.9]	22.3 [16 - 31.8]	364.2	TGO	2019	39.1 [37.7 - 40.4]	41.9 [33.3 - 47.8]	1102.5
GMB	2020	37.5 [36.1 - 38.8]		366.4	TGO	2020	38.2 [36.7 - 39.6]		1118.5
GNB	2010	44.9 [43.4 - 46.3]	43.5 [30.5 - 52.9]	234.6	TZA	2010	31.6 [30.3 - 32.9]	33.4 [28.3 - 38]	7144.0
GNB	2019	39.5 [38 - 40.9]	38.1 [22.7 - 49.5]	262.8	TZA	2019	31.7 [30.2 - 33.2]	27.9 [16.4 - 38.3]	8959.4
GNB	2020	38.5 [36.9 - 40]		263.6	TZA	2020	31 [29.5 - 32.5]		9169.5
GNQ	2010	44.2 [42.6 - 45.9]	39.3 [31.6 - 45.7]	163.8	UGA	2010	36.1 [35 - 37.3]	32.8 [27.2 - 38.1]	5505.3
GNQ	2019	37.8 [36 - 39.6]	34.4 [20.1 - 45.4]	200.5	UGA	2019	30.7 [29.5 - 31.9]	24.9 [18.1 - 32.1]	6526.2
GNQ	2020	36.9 [35.1 - 38.7]		203.1	UGA	2020	30 [28.8 - 31.3]		6674.1
IND	2010	37.5 [36 - 39.1]	31.3 [23.3 - 38.5]	114752.8	ZAF	2010	28 [25.1 - 31.1]	15.2 [7.1 - 26.7]	4856.1
IND	2019	34.2 [32.7 - 35.8]	26.4 [16.4 - 35.9]	106287.5	ZAF	2019	32.3 [28.8 - 36]	20.2 [7.5 - 38]	5183.1
IND	2020	31.9 [30.3 - 33.5]		105521.4	ZAF	2020	31.8 [28.2 - 35.5]		5191.0
KEN	2010	34.7 [33.2 - 36.3]	17.4 [11.8 - 23.7]	6101.8	ZMB	2010	31 [29.5 - 32.6]	26.2 [19.5 - 33.2]	2248.2
KEN	2019	34.7 [33.1 - 36.4]	19.2 [9.7 - 30.9]	6129.1	ZMB	2019	32.6 [31 - 34.3]	26.5 [16.8 - 35.6]	2656.1
KEN	2020	34.1 [32.4 - 35.8]		6169.4	ZMB	2020	32 [30.3 - 33.7]		2696.7
LBR	2010	44.5 [43 - 46]	41 [35.6 - 45.4]	604.2	ZWE	2010	26.1 [24.8 - 27.6]	23.1 [15.7 - 30.3]	1796.3
LBR	2019	40.3 [38.7 - 42]	41.8 [35.8 - 46.6]	658.3	ZWE	2019	27.8 [26.1 - 29.6]	14.3 [6.5 - 25.9]	2047.7
LBR	2020	39.2 [37.6 - 40.9]		661.7	ZWE	2020	27.2 [25.5 - 29]		2049.4

Population data is taken from the United Nations population prospects using the single age both sexes combined estimates. The population at risk is calculated by taking the weighted sum of the single age estimates (in years) and assuming that in the first year the population is uniformly distributed across months. This implies that apart from the first age bin, where the weight is 0.5 all other weights for the age bins are one. Country abbreviations correspond to the country-specific ISO3 country codes. *S*ource:[107–109], and own calculations.

Supplementary Table 8: Model-based prevalence and estimated prevalence (by WHO [107]) together with 95% uncertainty intervals of severe anemia (i.e., P(Hb < 70 g L^{-1}) in children aged 6 – 59 months at the country level for the years 2010, 2019, and 2020.

Country code	Year	Prevalence (%)	Prevalence WHO (%)	Population (in thousands)	Country code	Year	Prevalence (%)	Prevalence WHO (%)	Population (in thousands)
AFG	2010	1.9 [1.3 - 2.7]	1.9 [0.7 - 3.5]	4592.4	LSO	2010	1.4 [1.1 - 1.8]	1.3 [0.7 - 2.1]	230.6
AFG	2019	2 [1.3 - 2.9]	1.6 [0.3 - 4.6]	5579.3	LSO	2019	1.4 [1.1 - 1.8]	1.5 [0.3 - 4]	245.3
AFG	2020	2 [1.3 - 2.9]		5688.1	LSO	2020	1.3 [1 - 1.7]		245.7
AGO	2010	1.5 [1.3 - 1.8]	1.9 [1 - 3.3]	3868.5	MDG	2010	0.5 [0.2 - 1.1]	1 [0.6 - 1.5]	3144.9
AGO	2019	1.6 [1.3 - 1.8]	1.7 [0.6 - 3.8]	5071.9	MDG	2019	0.7 [0.2 - 1.7]	1.5 [0.5 - 3.5]	3570.0
AGO	2020	1.5 [1.2 - 1.7]		5192.4	MDG	2020	0.7 [0.2 - 1.7]		3636.8
BDI	2010	1.6 [1.5 - 1.8]	2.1 [1 - 3.5]	1544.4	MLI	2010	9.5 [8.8 - 10.2]	9.6 [7.8 - 11.2]	2662.3
BDI	2019	1.1 [1 - 1.3]	2.7 [1.1 - 5.1]	1848.2	MLI	2019	4.5 [4 - 4.9]	6.4 [4.1 - 9]	3384.5
BDI	2020	1 [0.9 - 1.2]		1846.0	MLI	2020	4.2 [3.7 - 4.6]		3465.5
BEN	2010	5.7 [5.3 - 6.2]	4.7 [3 - 6.8]	1426.7	MMR	2010	0.4 [0.3 - 0.6]	1.4 [0.4 - 3.2]	4142.6
BEN	2019	3.6 [3.3 - 3.9]	3.5 [1.8 - 5.5]	1817.6	MMR	2019	0.5 [0.3 - 0.7]	1.1 [0.3 - 2.7]	4047.5
BEN	2020	3.3 [3 - 3.7]		1859.6	MMR	2020	0.5 [0.3 - 0.7]		4051.5
BFA	2010	10.7 [10.1 - 11.4]	12.1 [9.6 - 13.7]	2612.5	MOZ	2010	4.5 [4 - 5]	3.6 [2.4 - 5]	3602.1
BFA	2019	4.2 [3.8 - 4.6]	5.7 [2.4 - 11.1]	3122.8	MOZ	2019	3.4 [3 - 3.9]	4.2 [1.8 - 7]	4471.0
BFA	2020	3.9 [3.5 - 4.3]		3153.4	MOZ	2020	3.2 [2.8 - 3.7]		4571.9
BGD	2010	0.6 [0.5 - 0.8]	0.7 [0.3 - 1.5]	14580.9	MRT	2010	5.3 [4.7 - 5.9]	4.3 [1.1 - 9.1]	508.5
BGD	2019	0.6 [0.4 - 0.7]	0.6 [0.1 - 2.3]	13144.5	MRT	2019	4.2 [3.7 - 4.7]	2.9 [0.6 - 7.6]	596.9
BGD	2020	0.5 [0.4 - 0.7]		13171.9	MRT	2020	4.1 [3.6 - 4.6]		607.8
BTN	2010	2.5 [1.9 - 3.3]	1.9 [0.5 - 4.3]	60.3	MWI	2010	3.2 [2.9 - 3.5]	3.5 [2.6 - 4.5]	2419.2
BTN	2019	2.8 [2.2 - 3.5]	0.9 [0.1 - 2.9]	50.1	MWI	2019	2 [1.7 - 2.2]	1.4 [0.6 - 2.6]	2639.2
BTN	2020	2.9 [2.3 - 3.5]		47.9	MWI	2020	1.8 [1.6 - 2.1]		2668.8
BWA	2010	1.3 [1 - 1.6]	0.6 [0.1 - 2.1]	239.0	NAM	2010	1.5 [1.1 - 2]	0.8 [0.3 - 1.8]	249.1
BWA	2019	1.4 [1.1 - 1.7]	0.8 [0.1 - 3.5]	264.1	NAM	2019	1.5 [1.1 - 2.1]	0.8 [0.1 - 2.6]	295.5
BWA	2020	1.3 [1 - 1.6]		265.6	NAM	2020	1.5 [1.1 - 2]		296.9
CAF	2010	2.4 [2.1 - 2.8]	7.4 [3.5 - 11.9]	734.4	NER	2010	6.6 [6 - 7.2]	5.9 [4 - 8.2]	2973.5
CAF	2019	1.3 [1.1 - 1.6]	5.7 [1.8 - 10.6]	826.1	NER	2019	3.9 [3.5 - 4.3]	3.5 [1 - 7.5]	4081.5
CAF	2020	1.2 [1 - 1.5]		843.5	NER	2020	3.6 [3.2 - 4.1]		4233.8
CIV	2010	6.5 [5.9 - 7]	5.5 [3.8 - 7.5]	3159.6	NGA	2010	5.8 [5.3 - 6.4]	5 [3.5 - 6.9]	25324.5
CIV	2019	3.1 [2.8 - 3.5]	4.9 [1.8 - 8.7]	3623.6	NGA	2019	3 [2.6 - 3.3]	3.4 [1.9 - 5.2]	29950.8
CIV	2020	2.9 [2.6 - 3.2]		3680.3	NGA	2020	2.7 [2.4 - 3.1]		30427.2
CMR	2010	3.2 [2.8 - 3.5]	2.8 [1.7 - 4.3]	3024.1	NPL	2010	0.7 [0.5 - 0.8]	0.6 [0.3 - 1]	2778.5
CMR	2019	1.9 [1.7 - 2.2]	2.3 [1 - 4.1]	3719.1	NPL	2019	2 [1.6 - 2.5]	0.5 [0.1 - 1.2]	2641.3
CMR	2020	1.8 [1.5 - 2.1]		3802.1	NPL	2020	1.8 [1.4 - 2.2]		2643.8
COD	2010	3.7 [3.3 - 4.2]	4.3 [2.8 - 6.2]	10884.3	PAK	2010	3.3 [2.7 - 4]	3.9 [1.7 - 6]	24384.7
COD	2019	2 [1.8 - 2.3]	3.2 [1.1 - 6.8]	14776.1	PAK	2019	2.6 [2 - 3.3]	3.1 [0.8 - 6.5]	26304.4
COD	2020	1.9 [1.6 - 2.2]		15212.3	PAK	2020	2.8 [2.1 - 3.5]		26364.6
COG	2010	2.2 [1.9 - 2.5]	1.6 [0.9 - 2.8]	640.5	RWA	2010	1 [0.8 - 1.1]	0.6 [0.3 - 1.1]	1472.6
COG	2019	1.6 [1.3 - 1.8]	1.4 [0.3 - 3.8]	754.3	RWA	2019	0.7 [0.6 - 0.8]	0.4 [0.2 - 1]	1632.0
COG	2020	1.4 [1.2 - 1.7]		755.8	RWA	2020	0.6 [0.6 - 0.7]		1659.9
DJI	2010	7.5 [6.7 - 8.4]	2.4 [0.5 - 6.6]	103.4	SEN	2010	4.3 [3.9 - 4.7]	6.3 [4.9 - 7.6]	1868.4
DJI	2019	5.9 [5.1 - 6.7]	1.9 [0.3 - 5.8]	103.3	SEN	2019	2.5 [2.2 - 2.8]	2.2 [1 - 3.8]	2231.2
DJI	2020	5.6 [4.9 - 6.4]		103.8	SEN	2020	2.3 [2 - 2.5]		2259.5
ERI	2010	2.4 [2 - 2.9]	1.8 [0.3 - 5.3]	444.6	SLE	2010	5.9 [5.5 - 6.4]	5.5 [3.8 - 7.5]	938.0
ERI	2019	1.7 [1.3 - 2.1]	1.5 [0.2 - 4.9]	433.9	SLE	2019	2.8 [2.5 - 3.1]	3.8 [2.3 - 5.7]	1036.3
ERI	2020	1.6 [1.3 - 2]		433.3	SLE	2020	2.6 [2.3 - 2.8]		1046.6
ETH	2010	4.6 [4 - 5.1]	3.3 [2.1 - 4.8]	13375.8	SOM	2010	9.4 [8.3 - 10.6]	4 [2.1 - 6.3]	2062.3
ETH	2019	3.1 [2.7 - 3.6]	2.8 [0.9 - 5.5]	15043.8	SOM	2019	8.3 [7.2 - 9.5]	2.5 [1.1 - 5]	2682.6
ETH	2020	3 [2.6 - 3.5]		15444.8	SOM	2020	8 [6.9 - 9.2]		2761.6
GAB	2010	1.4 [1.2 - 1.6]	2.3 [1.1 - 4.1]	217.4	SSD	2010	1.2 [1 - 1.4]	3.2 [0.7 - 7.4]	1442.0
GAB	2019	1.6 [1.4 - 1.9]	1.4 [0.3 - 3.8]	272.9	SSD	2019	0.7 [0.6 - 0.9]	3 [0.5 - 7.8]	1504.4
GAB	2020	1.5 [1.2 - 1.7]		274.4	SSD	2020	0.7 [0.5 - 0.8]		1421.9
GHA	2010	5.4 [5 - 5.8]	4.2 [2.5 - 6.3]	3384.4	SWZ	2010	1.3 [1.1 - 1.6]	0.9 [0.4 - 2]	136.4
GHA	2019	2.4 [2.2 - 2.7]	1.6 [0.9 - 2.6]	3860.6	SWZ	2019	1.3 [1.1 - 1.6]	0.9 [0.1 - 3.1]	129.0
GHA	2020	2.3 [2 - 2.5]		3882.0	SWZ	2020	1.2 [1 - 1.5]		127.4
GIN	2010	6.1 [5.6 - 6.5]	6.2 [4.3 - 8.3]	1538.3	TCD	2010	2.2 [1.9 - 2.6]	6.2 [2.9 - 10.1]	2104.6
GIN	2019	2.9 [2.6 - 3.2]	3.9 [1.8 - 7.2]	1821.8	TCD	2019	1.3 [1.1 - 1.6]	3.8 [2.5 - 5.4]	2713.8
GIN	2020	2.7 [2.4 - 3]		1848.9	TCD	2020	1.2 [1 - 1.5]		2779.0
GMB	2010	4.8 [4.4 - 5.1]	5.3 [2.7 - 8.1]	312.3	TGO	2010	4.4 [4 - 4.7]	4.5 [2.3 - 7.5]	945.2
GMB	2019	2 [1.8 - 2.3]	1.6 [0.8 - 2.7]	364.2	TGO	2019	2.4 [2.1 - 2.6]	3.8 [1.8 - 6.4]	1102.5
GMB	2020	1.8 [1.6 - 2]		366.4	TGO	2020	2.2 [1.9 - 2.4]		1118.5
GNB	2010	3.5 [3.2 - 3.9]	5.2 [1.6 - 10.1]	234.6	TZA	2010	1.5 [1.4 - 1.7]	2 [1.5 - 2.7]	7144.0
GNB	2019	2 [1.8 - 2.3]	3.5 [0.7 - 8.2]	262.8	TZA	2019	1.1 [1 - 1.3]	1.5 [0.6 - 3.2]	8959.4
GNB	2020	1.9 [1.6 - 2.1]		263.6	TZA	2020	1.1 [0.9 - 1.2]		9169.5
GNQ	2010	3.8 [3.4 - 4.2]	3.9 [2.1 - 6.1]	163.8	UGA	2010	3.6 [3.2 - 3.9]	3.4 [2.2 - 4.7]	5505.3
GNQ	2019	2 [1.7 - 2.3]	2.4 [0.6 - 5.9]	200.5	UGA	2019	1.9 [1.7 - 2.1]	1.6 [0.9 - 2.6]	6526.2
GNQ	2020	1.8 [1.6 - 2.1]		203.1	UGA	2020	1.7 [1.6 - 1.9]		6674.1
IND	2010	2.6 [2.2 - 3.1]	2.3 [1.2 - 3.8]	114752.8	ZAF	2010	1.7 [1.2 - 2.3]	1.1 [0.2 - 2.7]	4856.1
IND	2019	1.8 [1.5 - 2.1]	1.3 [0.5 - 2.7]	106287.5	ZAF	2019	1.8 [1.2 - 2.4]	1.5 [0.2 - 4.6]	5183.1
IND	2020	1.6 [1.2 - 1.9]		105521.4	ZAF	2020	1.7 [1.2 - 2.3]		5191.0
KEN	2010	2.8 [2.5 - 3.1]	1.7 [0.9 - 2.8]	6101.8	ZMB	2010	1.7 [1.5 - 2]	2.4 [1.3 - 3.9]	2248.2
KEN	2019	2.3 [2 - 2.6]	1.4 [0.4 - 3.5]	6129.1	ZMB	2019	1.4 [1.2 - 1.6]	1.4 [0.7 - 2.7]	2656.1
KEN	2020	2.2 [1.9 - 2.5]		6169.4	ZMB	2020	1.3 [1.1 - 1.5]		2696.7
LBR	2010	4.1 [3.7 - 4.5]	3 [1.8 - 4.6]	604.2	ZWE	2010	1.1 [1 - 1.3]	0.9 [0.5 - 1.4]	1796.3
LBR	2019	2.6 [2.3 - 2.9]	3.6 [2.3 - 5.2]	658.3	ZWE	2019	0.8 [0.7 - 1]	0.4 [0.1 - 1.3]	2047.7
LBR	2020	2.4 [2.1 - 2.7]		661.7	ZWE	2020	0.8 [0.6 - 0.9]		2049.4

Population data is taken from the United Nations population prospects using the single age both sexes combined estimates. The population at risk is calculated by taking the weighted sum of the single age estimates (in years) and assuming that in the first year the population is uniformly distributed across months. This implies that apart from the first age bin, where the weight is 0.5 all other weights for the age bins are one. Country abbreviations correspond to the country-specific ISO3 country codes. *S*ource: [107–109], and own calculations.

Supplementary Table 9: Comparison of studies on anemia, estimated anemia prevalence of this study, and estimated anemia prevalence of the WHO[107] among children together with 95% uncertainty intervals (if available) of any anemia (i.e., P(Hb < 110g L^{-1})) in children aged 6 – 59 months at the country level, 2009 – 2022.

Country code	Author	Year	Age (months)	Sample size	Comment	Prevalence of study (%)	Prevalence (%)	Prevalence WHO (%)
Articles								
AFG	Fahim et al.[110]	2013	6 – 59	905	National representative	44.8 [41.5 - 48]	57.1 [51.8 - 62.4]	43.5 [34.4 - 53.4]
AFG	Stanikzai et al.[111]	2021/2022	6 – 59	512	Kandahar city	55.6 [51.2 - 60]	57.8 [52.4 - 63.1] in 2020	44.9 [28.1 - 64.4] in 2019
BTN	Campbell et al.[112]	2015	6 – 59	1083	National Nutrition Survey	42.3 [35.8 - 48.8]	63.4 [60.7 - 66]	45.7 [32.7 - 60.8]
BTN	Chhetri et al.[113]	2016	6 – 59	353	Hospital-based	58.4	63.3 [60.6 - 65.9]	47.2 [35.8 - 60.4]
BWA	Leepile et al.[114]	2019	6 – 59	367	Indigenous San People	68	62.8 [59.5 - 66.1]	43.3 [21.7 - 67.2]
CAF	Vonaesch et al.[115]	2017/2018	24 – 60	409	Bangui	47	57.2 [54.7 - 59.6]	75.2 [63.1 - 83.2]
GNB	Thorne et al.[116]	2012	6 – 59	440	Bijagós Archipelago	80.2 [76.3 - 83.7]	73.6 [72.2 - 75.1]	72.2 [57.2 - 81.7]
GNB	Silva and Abecasis[117]	2023	6 – 59	1608	National representative	55.9	69.5 [67.8 - 71.2] in 2020	68 [50.1 - 79.9] in 2019
GNQ	Ncogo et al.[118]	2013	2 – 59	350	Bata district	87.7	74.1 [72.3 - 75.9]	67.5 [58.4 - 74.9]
GNQ	Ncogo et al.[118]	2013	13 – 59	441	Bata district	85.6	74.1 [72.3 - 75.9]	67.5 [58.4 - 74.9]
PAK	Habib et al.[119]	2011/2012	6 – 59	7138	National Nutrition Survey	62.3 [58.2 - 65.5]	63.9 [60.9 - 66.8]	58.2 [48.7 - 66.8]
PAK	Habib et al.[120]	2018	6 – 59	17814	National Nutrition Survey	53.7	60.8 [57.8 - 63.8]	53.8 [40.3 - 67.1]
SOM	Wirth et al.[121]	2019	6 – 59	1667	Somalia Micronutrient Survey	43.4 [40 - 46.9]	78.2 [75.7 - 80.7]	51.8 [39.7 - 65.8]
TCD	Zavala et al.[122]	2016	6 – 59	9109	National representative	68.6 [67.7 - 69.6]	58 [55.1 - 60.8]	70.9 [63.7 - 76.7]
TCD	Zavala et al.[122]	2021	6 – 59	6751	National representative	59.6 [58.5 - 60.8]	54.6 [51.5 - 57.6]	66.3 [59.8 - 72.9] in 2019
Surveys								
BDI	UNHCR[123]	2019	6 – 59	126	Burundi refugee camps (RC), COD	56.5 [52.3 - 60.6]	55.2 [53.6 - 56.8]	58 [46.3 - 68.9]
BTN	UNHCR[124]	2018	6 – 59	216	Bhutanese RC, NPL	35.7 [27.4 - 44.7]	63.1 [60.5 - 65.7]	45.2 [30.6 - 61.5]
BWA	UNHCR[125]	2013	6 – 59	165	Dukwi RC, BWA	50.9 [43 - 58.8]	56.3 [53.3 - 59.4]	39.1 [21.4 - 60.2]
CAF	UNHCR[126]	2019	6 – 59	249	Makpandu RC, SSD	60.3 [54 - 66.4]	54.2 [51.6 - 56.8]	73.6 [59.2 - 83.4]
CAF	UNHCR[127]	2019	6 – 59	1820	Central African Republic RC, COD	56.4 [54.2 - 58.8]	54.2 [51.6 - 56.8]	73.6 [59.2 - 83.4]
COD	UNHCR[126]	2019	6 – 59	249	Makpandu RC, SSD	60.3 [54 - 66.4]	63.5 [61.3 - 65.7]	64.9 [50.3 - 76.6]
DJI	WFP[128]	2011	pre-school	–	Djibouti	65.8	74.8 [72.6 - 77]	52.8 [34.3 - 72.1]
ERI	UNHCR[126]	2019	6 – 59	249	Makpandu RC, SSD	60.3 [54 - 66.4]	53.2 [49.8 - 56.5]	48.8 [29 - 69.7]
MRT	DHS[129]	2019-2021	6 – 59	10077	National representative	76.7	74.1 [72.1 - 76.1] in 2020	65.5 [47.5 - 78] in 2019
SOM	FSNAU[130]	2009	6 – 59	784	FSNAU	59.3 [54.8 - 63.6	78.2 [75.8 - 80.5]	59.3 [48.2 - 69.3]
SOM	UNHCR[131]	2018	6 – 59	415	Somali RC Bokolmanyo, ETH	44.8 [40 - 49.8]	78.8 [76.4 - 81.1]	52.7 [41.7 - 65.6]
SOM	UNHCR[131]	2018	6 – 59	360	Somali RC Melkadida, ETH	45.3 [40.1 - 50.6]	78.8 [76.4 - 81.1]	52.7 [41.7 - 65.6]
SOM	UNHCR[131]	2018	6 – 59	307	Somali RC Kobe, ETH	60.3 [54.5 - 65.8]	78.8 [76.4 - 81.1]	52.7 [41.7 - 65.6]
SOM	UNHCR[131]	2018	6 – 59	405	Somali RC Hilaweyn, ETH	44.7 [39.9 – 49.6]	78.8 [76.4 - 81.1]	52.7 [41.7 - 65.6]
SOM	UNHCR[131]	2018	6 – 59	383	Somali RC Buramino, ETH	47.5 [42.4 – 52.7]	78.8 [76.4 - 81.1]	52.7 [41.7 - 65.6]
SSD	UNHCR[132]	2019	6 – 59	420	South Sudan RC, COD	66 [61.3 - 70.3]	45.9 [43.2 - 48.6]	60.5 [39.5 - 77.4]
SSD	UNHCR[133]	2019	6 – 59	249	Gorom RC, SSD	70.7	45.9 [43.2 - 48.6]	60.5 [39.5 - 77.4]
SSD	UNHCR[134]	2019	6 – 59	258	Pamir RC, SSD	47.3 [41.1 - 53.6]	45.9 [43.2 - 48.6]	60.5 [39.5 - 77.4]
SSD	UNHCR[135]	2019	6 – 59	611	Doro RC, SSD	55.8 [51.9 - 59.7]	45.9 [43.2 - 48.6]	60.5 [39.5 - 77.4]
SSD	UNHCR[135]	2019	6 – 59	546	Yusuf Batil RC, SSD	55.7 [51.5 - 59.8]	45.9 [43.2 - 48.6]	60.5 [39.5 - 77.4]
SSD	UNHCR[135]	2019	6 – 59	678	Gendrassa RC, SSD	57.5 [53.8 - 61.2]	45.9 [43.2 - 48.6]	60.5 [39.5 - 77.4]
SSD	UNHCR[135]	2019	6 – 59	551	Kaya RC, SSD	49.9 [45.8 - 54.1]	45.9 [43.2 - 48.6]	60.5 [39.5 - 77.4]
TCD	UNHCR[136]	2016	6 – 59	343	Doholo RC, TCD	76.4 [71.6 - 80.6]	58 [55.1 - 60.8]	70.9 [63.7 - 76.7]
TCD	UNHCR[136]	2016	6 – 59	464	Dosseye RC, TCD	51.9 [45.6 - 58.3]	58 [55.1 - 60.8]	70.9 [63.7 - 76.7]
TCD	UNHCR[136]	2016	6 – 59	344	Gondjé RC, TCD	73.5 [67.5 - 79.6]	58 [55.1 - 60.8]	70.9 [63.7 - 76.7]
TCD	UNHCR[136]	2016	6 – 59	395	Amboko RC, TCD	66.6 [60.5 - 72.7]	58 [55.1 - 60.8]	70.9 [63.7 - 76.7]
TCD	UNHCR[136]	2016	6 – 59	410	Belom RC, TCD	59.3 [54.3 - 64.2]	58 [55.1 - 60.8]	70.9 [63.7 - 76.7]
TCD	UNHCR[136]	2016	6 – 59	549	Moyo RC, TCD	48.6 [42.8 - 54.4]	58 [55.1 - 60.8]	70.9 [63.7 - 76.7]
TCD	UNHCR[136]	2016	6 – 59	483	Daressalam RC, TCD	54.5 [49.2 - 59.7]	58 [55.1 - 60.8]	70.9 [63.7 - 76.7]
TCD	UNHCR[137]	2021	6 – 59	10072	Ensemble RC, TCD	48.1 [46.5 - 49.8]	58 [55.1 - 60.8]	70.9 [63.7 - 76.7]

Note that in the Makpandu refugee camp[126] most refugees come from four African countries (i.e., CAF, COD, SDN, and ERI). Country abbreviations correspond to the country-specific ISO3 country codes. *A*dditional sources:[107,108], and own calculations.

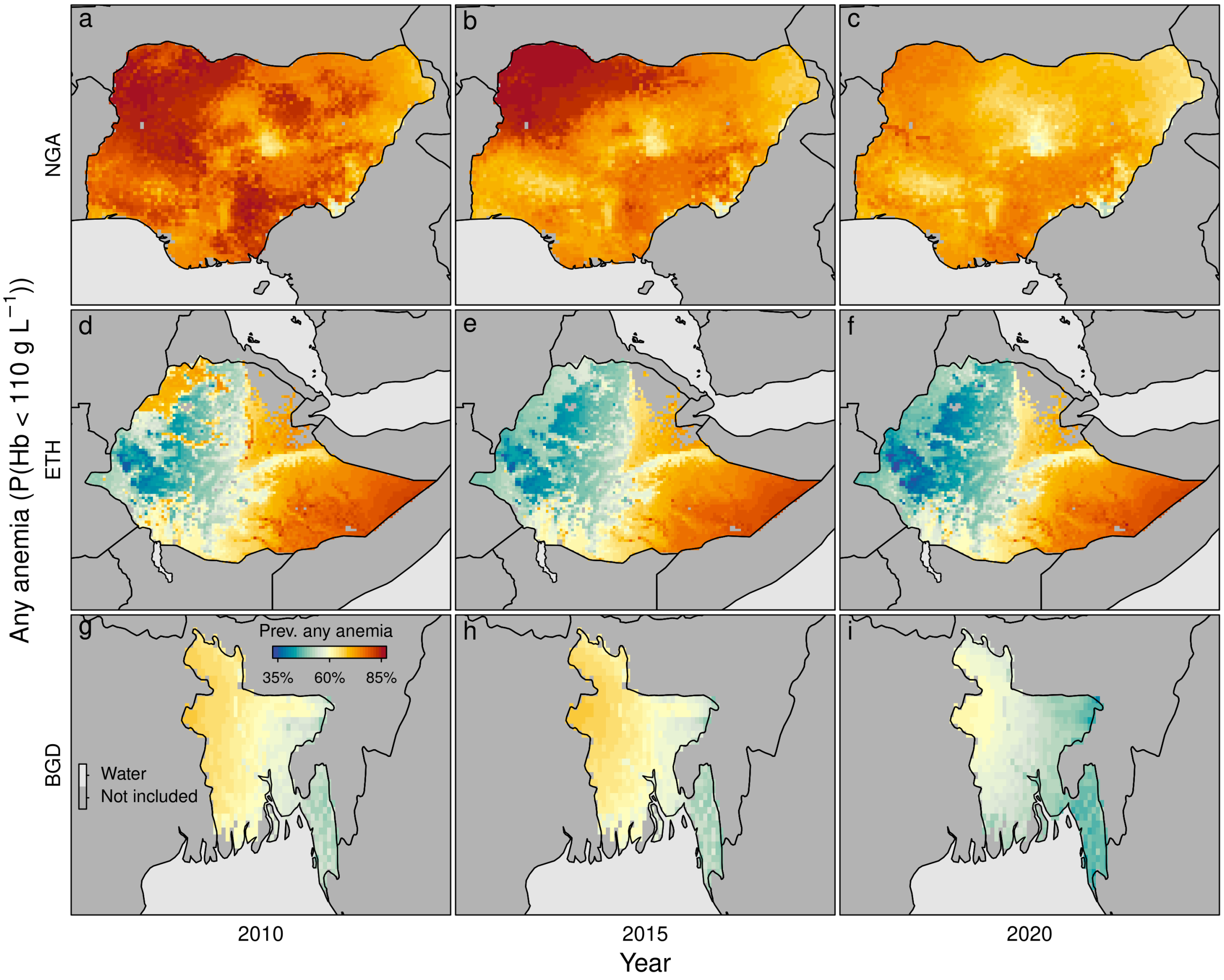

Supplementary Figure 17: **Predicted marginal spatio-temporal prevalence of any form of anemia (i.e., $\mathbf{P(Hb < 110\ g\ L^{-1})}$) among children aged 6 to 59 months. a, b,** and **c** show the estimated overall anemia prevalence for Nigeria for the years 2010, 2015, and 2020, respectively; **d, e,** and **f** show the estimated overall anemia prevalence for Ethiopia for the years 2010, 2015, and 2020, respectively; and **g, h,** and **i** show the estimated overall anemia prevalence for Bangladesh for the years 2010, 2015, and 2020, respectively. Note that the non-geo-referenced covariates are fixed at the median (metric covariates) or the mode (categorical covariates), respectively. Note that for illustration purposes the estimates of the neighboring countries are omitted. Further, note that the boundaries reflect administrative boundaries at the country level. Pixels categorized as *Barren*, or *Permanent Snow and Ice*, and pixels above $3,750$ m ($1,900$ m) of altitude in sub-Saharan Africa and South Asia (Madagascar) are flagged as *Not included.* ISO3 country codes are used as identifiers, where NGA for Nigeria, ETH stands for Ethiopia, and BGD for Bangladesh.

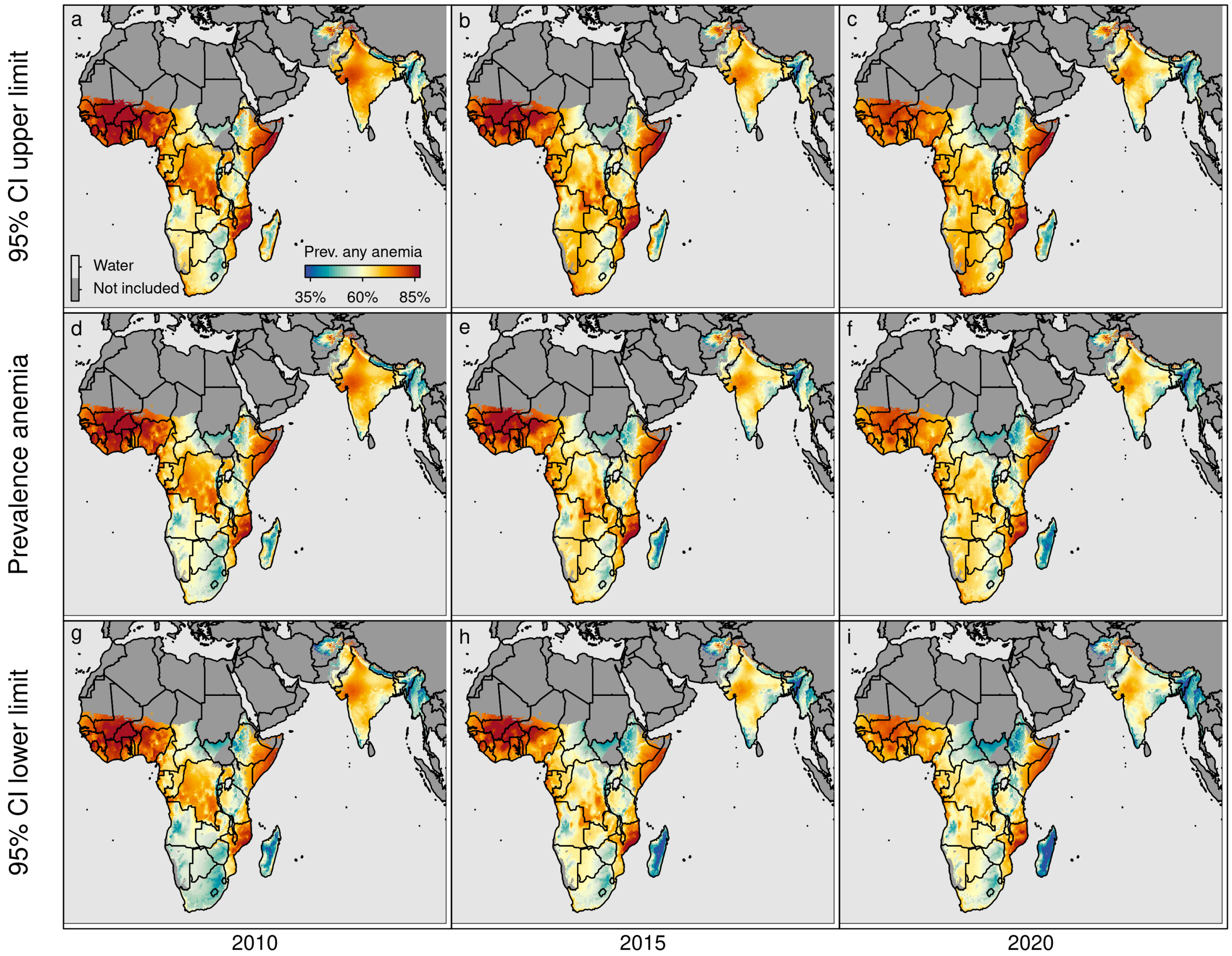

Supplementary Figure 18: **Predicted marginal spatio-temporal prevalence of any form of anemia (i.e., $\mathbf{P(Hb < 110\ g\ L^{-1})}$) of boys (6-59 months) together with 95% credible intervals for the years 2010, 2015, and 2020.** The maps show the estimated anemia prevalence among boys aged 6 to 59 months (**d, e,** and **f**) together with 95% credible intervals (**a, b, c, g, h,** and **i**) for the years 2010, 2015, and 2020. Boundaries reflect administrative boundaries at the country level. Note that the non-geo-referenced covariates are fixed at the median (metric covariates) or the mode (categorical covariates), respectively. Note that the boundaries reflect administrative boundaries at the country level. Furthermore, note that even though the color palettes are identical across rows, the corresponding values differ across rows. In addition, note that pixels categorized as *Barren*, or *Permanent Snow and Ice*, and pixels above $3,750$ m ($1,900$ m) of altitude in sub-Saharan Africa and South Asia (Madagascar) are flagged as *Not included*.

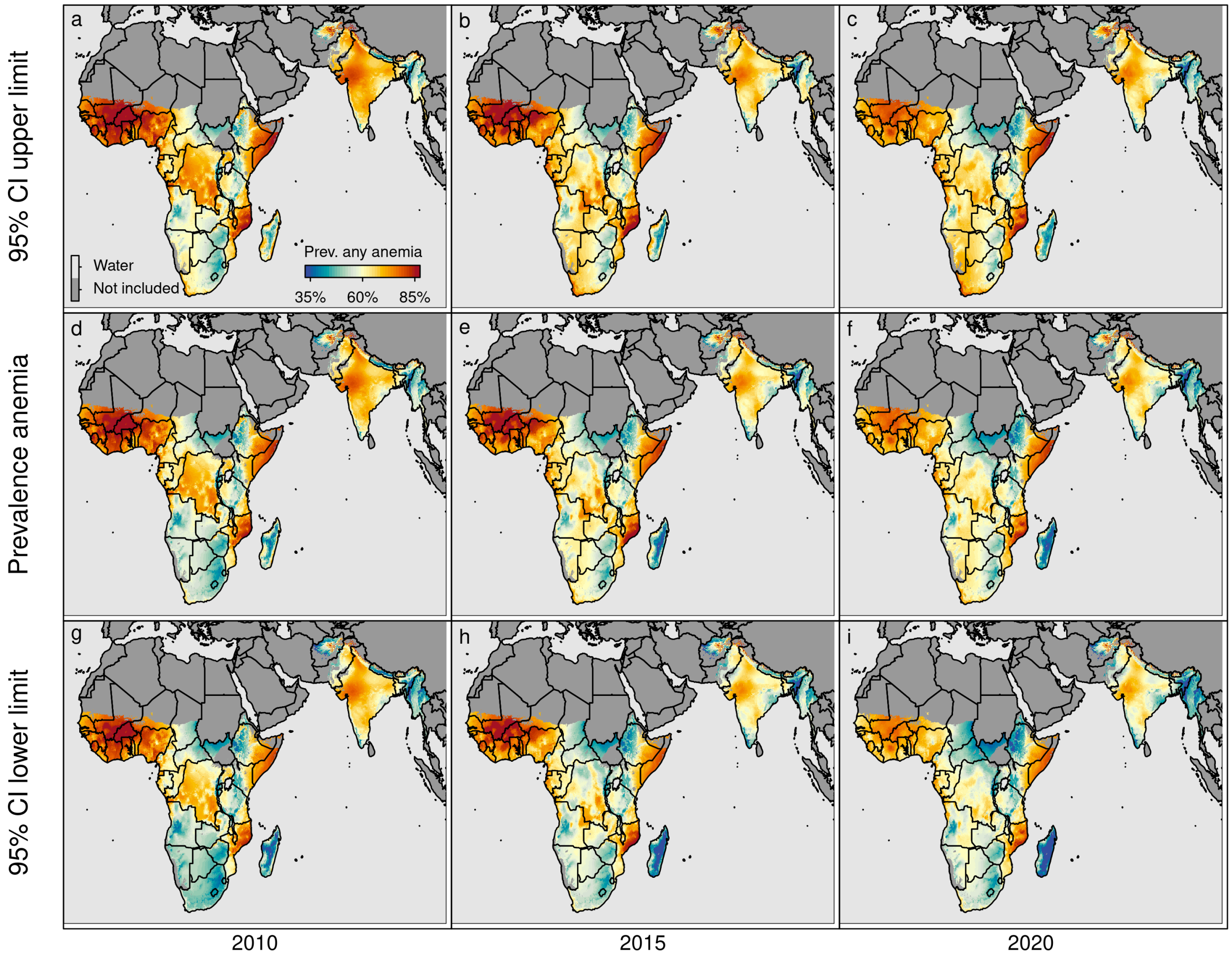

Supplementary Figure 19: **Predicted marginal spatio-temporal prevalence of any form of anemia (i.e., P(Hb $<$ 110 g L^{-1})) of girls (6-59 months) together with 95% credible intervals for the years 2010, 2015, and 2020.** The maps show the estimated anemia prevalence among girls aged 6 to 59 months (**d, e,** and **f**) together with 95% credible intervals (**a, b, c, g, h,** and **i**) for the years 2010, 2015, and 2020. Boundaries reflect administrative boundaries at the country level. Note that the non-geo-referenced covariates are fixed at the median (metric covariates) or the mode (categorical covariates), respectively. Note that the boundaries reflect administrative boundaries at the country level. Furthermore, note that even though the color palettes are identical across rows, the corresponding values differ across rows. In addition, note that pixels categorized as *Barren*, or *Permanent Snow and Ice*, and pixels above $3,750$ m ($1,900$ m) of altitude in sub-Saharan Africa and South Asia (Madagascar) are flagged as *Not included*.

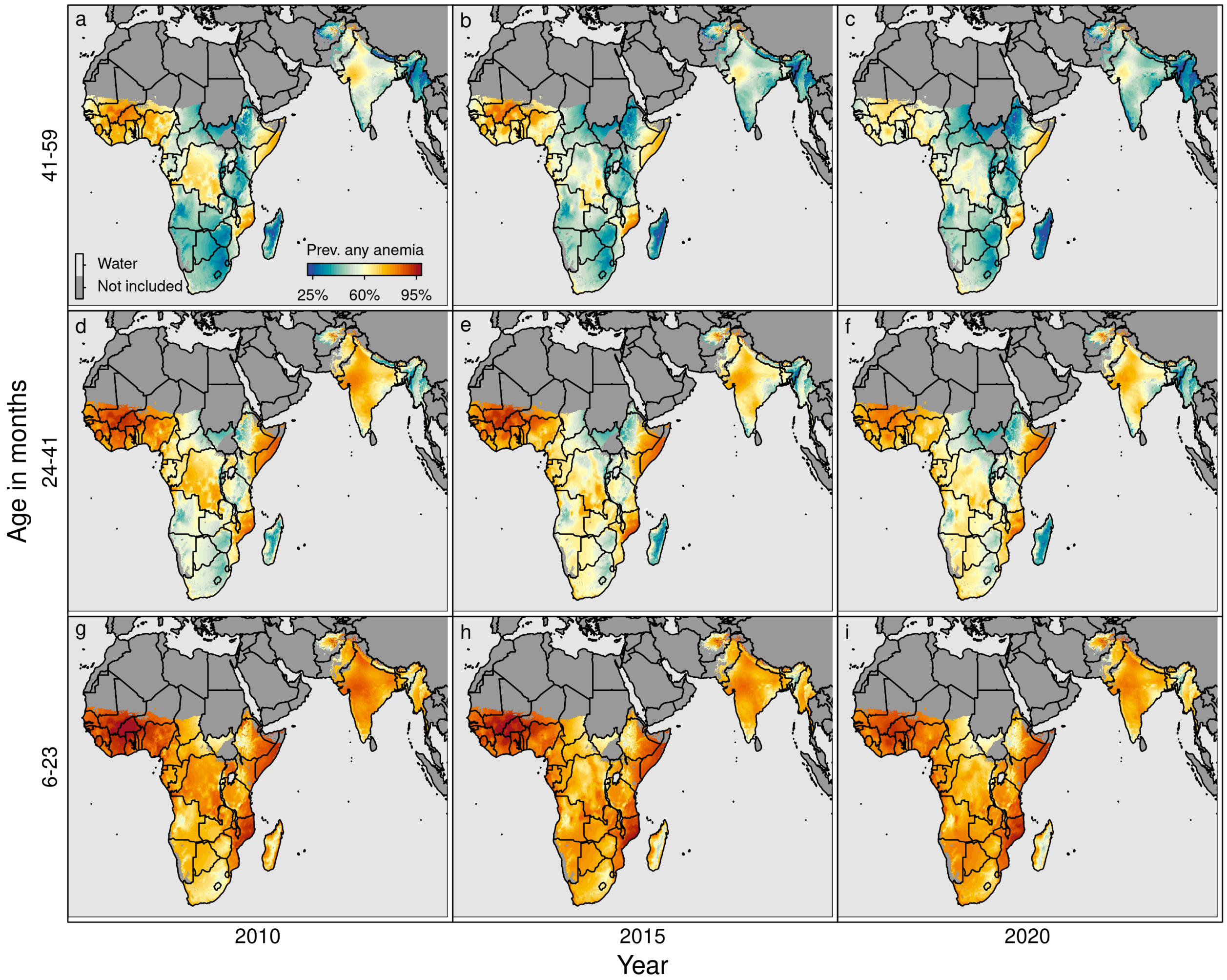

Supplementary Figure 20: **Predicted marginal spatio-temporal prevalence of any form of anemia (i.e., P(Hb $<$ 110 g L^{-1})) of boys.** The maps show the estimated anemia prevalence among boys aged 6 to 59 months for the years 2010, 2015, and 2020 for the following three age bins: (**a, b, c**) $(42, 60]$ months; (**d, e, f**) $(24, 42]$ months; and (**g, h, i**) $(6, 24]$ months), respectively. The x-axis shows the dynamics over time, the y-axis shows the age-specific dynamics of the overall prevalence of anemia. Note that the non-geo-referenced covariates are fixed at the median (metric covariates) or the mode (categorical covariates), respectively. Boundaries are at the country level. Pixels categorized as *Barren*, or *Permanent Snow and Ice*, and pixels above $3,750$ m $(1,900$ m$)$ of altitude in sub-Saharan Africa and South Asia (Madagascar) are flagged as *Not included*.

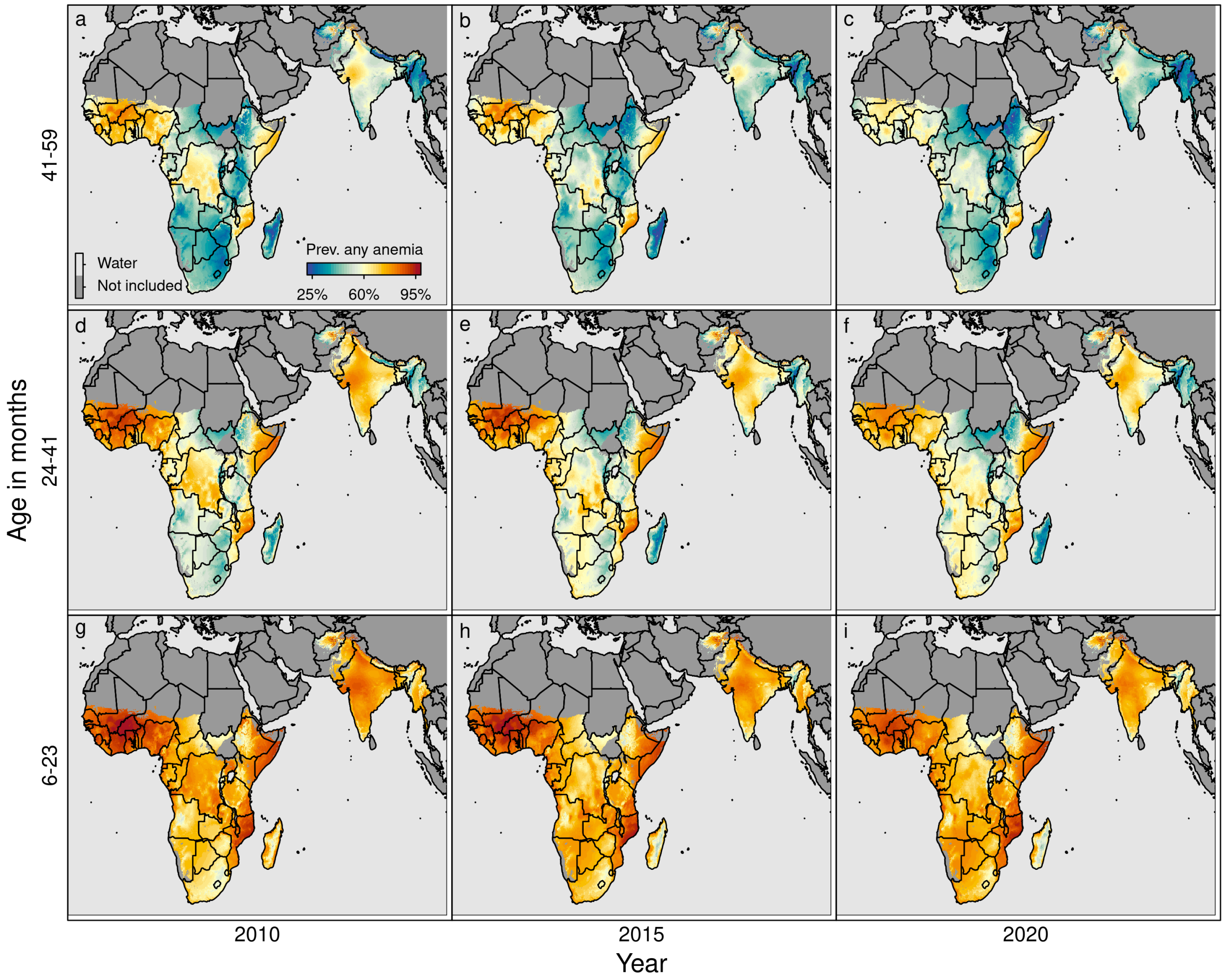

Supplementary Figure 21: **Predicted marginal spatio-temporal prevalence of any form of anemia (i.e., $\mathbf{P}(\mathbf{Hb} < 110\ \mathbf{g}\ \mathbf{L}^{-1})$) of girls.** The maps show the estimated anemia prevalence among girls aged 6 to 59 months for the years 2010, 2015, and 2020 for the following three age bins: (**a, b, c**) $(42, 60]$ months; (**d, e, f**) $(24, 42]$ months; and (**g, h, i**) $(6, 24]$ months), respectively. The x-axis shows the dynamics over time, the y-axis shows the age-specific dynamics of the overall prevalence of anemia. Note that the non-geo-referenced covariates are fixed at the median (metric covariates) or the mode (categorical covariates), respectively. Boundaries are at the country level. Pixels categorized as *Barren*, or *Permanent Snow and Ice*, and pixels above 3,750 m (1,900 m) of altitude in sub-Saharan Africa and South Asia (Madagascar) are flagged as *Not included*.

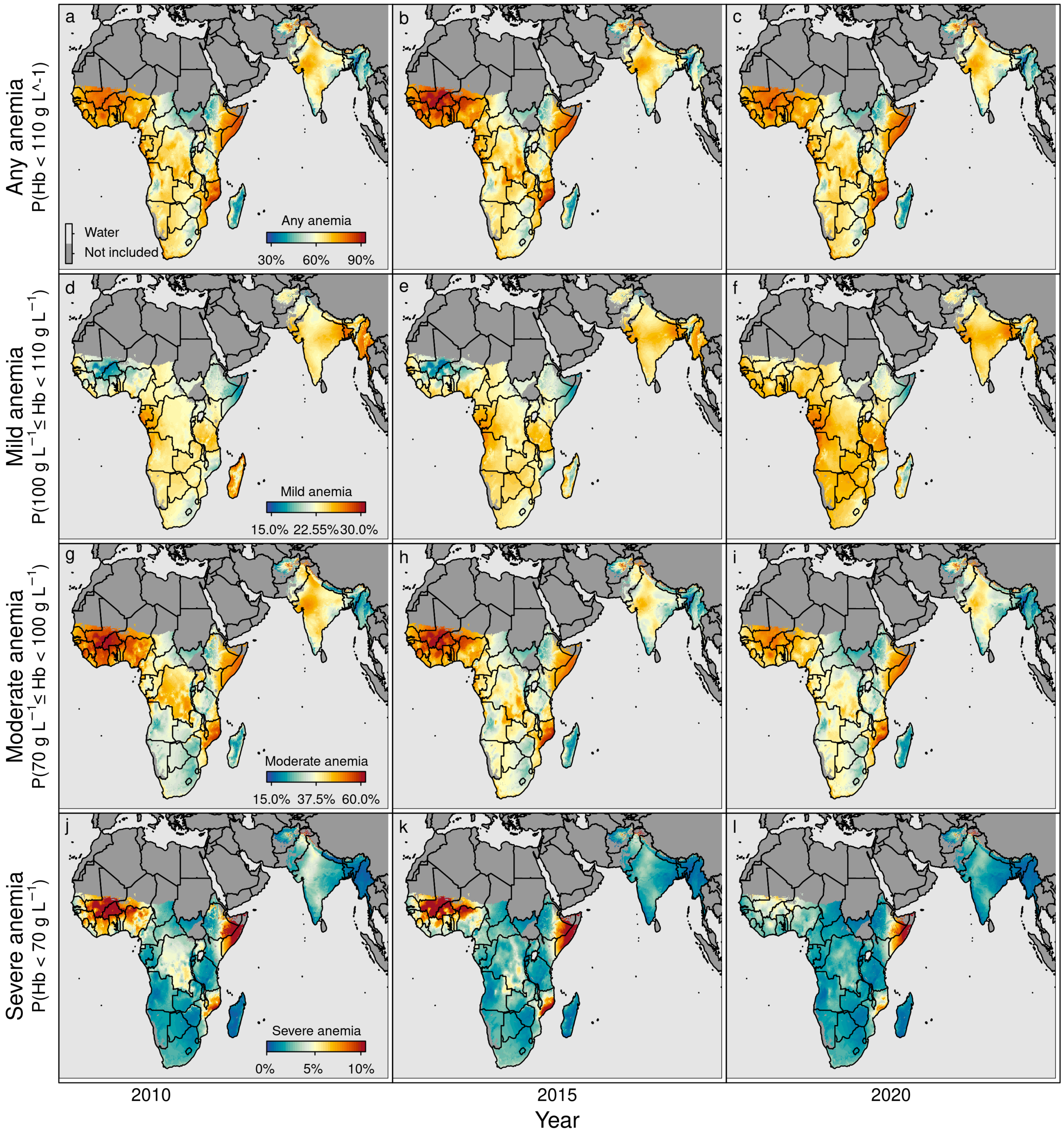

Supplementary Figure 22: **Predicted marginal spatio-temporal prevalence of anemia of boys. (a, b, c)** any anemia (i.e., $\text{P}(\text{Hb} < 110 \text{ g L}^{-1})$); **(d, e, f)** mild anemia (i.e., $\text{P}(100 \text{ g L}^{-1} \leq \text{Hb} < 110 \text{ g L}^{-1})$); **(g, h, i)** moderate anemia (i.e., $\text{P}(70 \text{ g L}^{-1} \leq \text{Hb} < 100 \text{ g L}^{-1}$); and **(j, k, l)** severe anemia (i.e., $\text{P}(\text{Hb} < 70 \text{ g L}^{-1})$). The maps show the estimated anemia prevalence by severity among boys aged 6 to 59 months for the years 2010, 2015 and 2020, respectively. Note that the non-geo-referenced covariates are fixed at the median (metric covariates) or the mode (categorical covariates), respectively. Note that the boundaries reflect administrative boundaries at the country level. Furthermore, note that even though the color palettes are identical across rows, the corresponding values differ across rows. In addition, note that pixels categorized as *Barren*, or *Permanent Snow and Ice*, and pixels above 3,750 m (1,900 m) of altitude in sub-Saharan Africa and South Asia (Madagascar) are flagged as *Not included*.

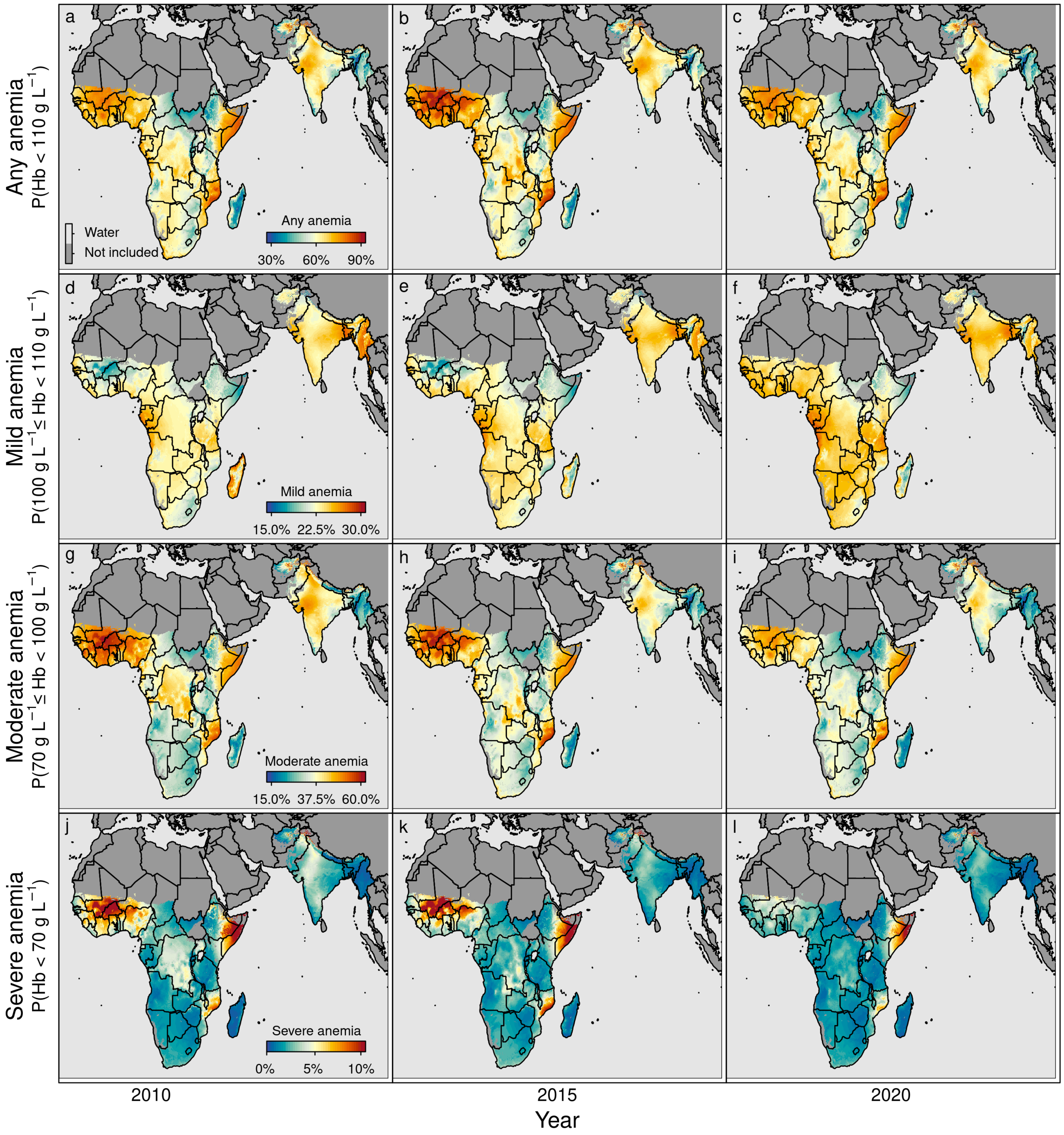

Supplementary Figure 23: **Predicted marginal spatio-temporal prevalence of anemia of girls. (a, b, c)** any anemia (i.e., P(Hb $<$ 110 g L^{-1})); **(d, e, f)** mild anemia (i.e., P(100 g L^{-1} $\leq$ Hb $<$ 110 g L^{-1})); **(g, h, i)** moderate anemia (i.e., P(70 g L^{-1} $\leq$ Hb $<$ 100 g L^{-1}); and **(j, k, l)** severe anemia (i.e., P(Hb $<$ 70 g L^{-1})). The maps show the estimated anemia prevalence by severity among girls aged 6 to 59 months for the years 2010, 2015 and 2020, respectively. Note that the non-geo-referenced covariates are fixed at the median (metric covariates) or the mode (categorical covariates), respectively. Note that the boundaries reflect administrative boundaries at the country level. Furthermore, note that even though the color palettes are identical across rows, the corresponding values differ across rows. In addition, note that pixels categorized as *Barren*, or *Permanent Snow and Ice*, and pixels above 3,750 m (1,900 m) of altitude in sub-Saharan Africa and South Asia (Madagascar) are flagged as *Not included*.

References

[1] Amante, C. & Eakins, B. ETOPO1 1 arc-minute global relief model: procedures, data sources and analysis (2009). Retrieved January 27, 2021, from NOAA Technical Memorandum NESDIS NGDC-24 doi: 10.7289/V5C8276M.

[2] NOAA National Geophysical Data Center. ETOPO1 1 arc-minute global relief model [Dataset] (2009). Retrieved January 27, 2021, from https://www.ncei.noaa.gov/access/metadata/landing-page/bin/iso?id=gov.noaa.ngdc.mgg.dem:316.

[3] Davies, S., Pettersson, T. & Öberg, M. Organized violence 1989–2021 and drone warfare. *Journal of Peace Research* **59**, 593–610 (2022). doi: 10.1177/00223433221108428.

[4] Sundberg, R. & Melander, E. Introducing the UCDP georeferenced event dataset. *Journal of Peace Research* **50**, 523–532 (2013). doi: 10.1177/0022343313484347.

[5] World Bank. World Development Indicators 2022 (2022). Retrieved November 04, 2022 https://data.worldbank.org/.

[6] Friedl, M. A. *et al.* MODIS collection 5 global land cover: algorithm refinements and characterization of new datasets. *Remote Sensing of Environment* **114**, 168–182 (2010). doi: 10.1016/j.rse.2009.08.016.

[7] Friedl, M. & Sulla-Menashe, D. MCD12C1 MODIS/Terra+Aqua land cover type yearly L3 global 0.05Deg CMG V006 [Dataset] (2015). Retrieved July 07, 2022, from doi: 10.5067/MODIS/MCD12C1.006.

[8] Weiss, D. J. *et al.* Mapping the global prevalence, incidence, and mortality of *Plasmodium falciparum*, 2000-17: a spatial and temporal modelling study. *Lancet* **394**, 322–331 (2019). doi: 10.1016/S0140-6736(19)31097-9.

[9] Battle, K. *et al.* Mapping the global endemicity and clinical burden of *Plasmodium vivax*, 2000-17: a spatial and temporal modelling study. *Lancet* **394**, 332–343 (2019). doi: 10.1016/S0140-6736(19)31096-7.

[10] Gething, P. W. *et al.* Modelling the global constraints of temperature on transmission of *Plasmodium falciparum* and *P. vivax*. *Parasites & Vectors* **4**, 92 (2011). doi: 10.1186/1756-3305-4-92.

[11] Gething, P. W. *et al.* A new world malaria map: *Plasmodium falciparum* endemicity in 2010. *Malaria Journal* **10**, 378 (2011). doi: 10.1186/1475-2875-10-378.

[12] Gething, P. W. *et al.* A long neglected world malaria map: *Plasmodium vivax* endemicity in 2010. *PLOS Neglected Tropical Diseases* **6**, e1814 (2012). doi: 10.1371/journal.pntd.0001814.

[13] Li, X., Zhou, Y., Zhao, M. & Zhao, X. A harmonized global nighttime light dataset 1992-2018. *Scientific Data* **7**, 168 (2020). doi: 10.1038/s41597-020-0510-y.

[14] Tucker, C. J. *et al.* An extended AVHRR 8-km NDVI dataset compatible with MODIS and SPOT vegetation NDVI data. *International Journal of Remote Sensing* **26**, 4485–4498 (2005). doi: 10.1080/01431160500168686.

[15] Pinzon, J. E. & Tucker, C. J. A non-stationary 1981–2012 AVHRR $NDVI_{3g}$ time series. *Remote Sensing* **6**, 6929–6960 (2014). doi: 10.3390/rs6086929.

[16] Didan, K. & Barreto, A. Nasa measures vegetation index and phenology (VIP) phenology EVI2 yearly global 0.05Deg CMG [Dataset] (2016). doi: 10.5067/MEaSUREs/VIP/VIPPHEN_EVI2.004.

[17] Hersbach, H. *et al.* The ERA5 global reanalysis. *Quarterly Journal of the Royal Meteorological Society* **146**, 1999–2049 (2020). doi: 10.1002/qj.3803.

[18] Poggio, L. *et al.* SoilGrids 2.0: producing soil information for the globe with quantified spatial uncertainty [Dataset]. *SOIL* **7**, 217–240 (2020). doi: 10.5194/soil-7-217-2021.

[19] Weiss, D. J. *et al.* Global maps of travel time to healthcare facilities. *Nature Medicine* **26**, 1835–1838 (2020). doi: 10.1038/s41591-020-1059-1.

[20] Weiss, D. J. *et al.* A global map of travel time to cities to assess inequalities in accessibility in 2015. *Nature* **553**, 333–336 (2018). doi: 10.1038/nature25181.

[21] NASA JPL. NASA shuttle radar topography mission water body data shapefiles & raster files [Dataset] (2013). Retrieved May 25, 2021, from doi: 10.5067/MEaSUREs/SRTM/SRTMSWBD.003.

[22] ICF. *Demographic and Health Surveys (various) [Datasets]* (ICF [Distributor], Rockville, Maryland, USA, 2004-2017). Funded by USAID.

[23] WHO. Global malaria programme (2021). Retrieved May 24, 2024, from `https://www.who.int/teams/global-malaria-programme/elimination/countries-and-territories-certified-malaria-free-by-who`.

[24] WHO. Worldwide prevalence of anaemia 1993-2005: WHO global database on anaemia (2008). Edited by Bruno de Benoist, Erin McLean, Ines Egli and Mary Cogswell. Retrieved November 24, 2023, from `https://apps.who.int/iris/handle/10665/43894`.

[25] Pullum, T., Kortso Collison, D., Namaste, S. & Garret, D. Hemoglobin data in DHS surveys: Intrinsic variation and measurement error. DHS Methodological Reports No. 18, ICF International, Rockville, Maryland, USA (2017). Retrieved from `http://dhsprogram.com/pubs/pdf/MR18/MR18.pdf`.

[26] Kotepui, M., Kotepui, K. U., Milanez, G. D. & Masangkay, F. R. Global prevalence and mortality of severe *Plasmodium malariae* infection: a systematic review and meta-analysis. *Malaria Journal* **19**, 274 (2020). doi: 10.1186/s12936-020-03344-z.

[27] Looareesuwan, S. *et al.* Erythrocyte survival in severe falciparum malaria. *Acta Tropica* **48**, 263–270 (1991). doi: 10.1016/0001-706X(91)90014-B.

[28] Muriuki, J. M. *et al.* Malaria is a cause of iron deficiency in African children. *Nature Medicine* **27**, 653–658 (2021). doi: 10.1038/s41591-021-01238-4.

[29] White, N. J. Anaemia and malaria. *Malaria Journal* **17**, 371 (2018). doi: 10.1186/s12936-018-2509-9.

[30] WHO. Malaria (2022). Retrieved September 1, 2022, from `https://www.who.int/news-room/fact-sheets/detail/malaria`.

[31] Balarajan, Y., Ramakrishnan, U., Özaltin, E., Shankar, A. H. & Subramanian, S. V. Anaemia in low-income and middle-income countries. *Lancet* **378**, 2123–2135 (2011). doi: 10.1016/S0140-6736(10)62304-5.

[32] Soares Magalhães, R. J. & Clements, A. C. A. Spatial heterogeneity of haemoglobin concentration in preschool-age children in sub-Saharan Africa. *Bulletin of the World Health Organization* **89**, 459–468 (2011). doi: 10.2471/BLT.10.083568.

[33] Kassebaum, N. J. *et al.* A systematic analysis of global anemia burden from 1990 to 2010. *Blood* **123**, 615–624 (2014). doi: 10.1182/blood-2013-06-508325.

[34] Brooker, S. *et al.* The co-distribution of *Plasmodium falciparum* and hookworm among African schoolchildren. *Malaria Journal* **5**, 99 (2006). doi: 10.1186/1475-2875-5-99.

[35] Karagiannis-Voules, D.-A. *et al.* Spatial and temporal distribution of soil-transmitted helminth infection in sub-Saharan Africa: a systematic review and geostatistical meta-analysis. *Lancet Infectious Diseases* **15**, 74–84 (2015). doi: 10.1016/S1473-3099(14)71004-7.

[36] Weaver, H. J., Hawdon, J. M. & Hoberg, E. P. Soil-transmitted helminthiases: implications of climate change and human behavior. *Trends in Parasitology* **26**, 574–581 (2010). doi: 10.1016/j.pt.2010.06.009.

[37] Kokaliaris, C. *et al.* Effect of preventive chemotherapy with praziquantel on schistosomiasis among school-aged children in sub-Saharan Africa: a spatiotemporal modelling study. *Lancet Infectious Diseases* **22**, 136–149 (2022). doi: 10.1016/S1473-3099(21)00090-6.

[38] Lai, Y.-S. *et al.* Spatial distribution of schistosomiasis and treatment needs in sub-Saharan Africa: a systematic review and geostatistical analysis. *Lancet Infectious Diseases* **15**, 927–940 (2015). doi: 10.1016/S1473-3099(15)00066-3.

[39] Soares Magalhães, R. J. & Clements, A. C. A. Mapping the risk of anaemia in preschool-age children: the contribution of malnutrition, malaria, and helminth infections in west Africa. *PLOS Medicine* **8**, e1000438 (2011). doi: 10.1371/journal.pmed.1000438.

[40] Moschovis, P. P. *et al.* Individual, maternal and household risk factors for anaemia among young children in sub-Saharan Africa: a cross-sectional study. *BMJ Open* **8**, e019654 (2018). doi: 10.1136/bmjopen-2017-019654.

[41] Verbesselt, J. *et al.* Remotely sensed resilience of tropical forests. *Nature Climate Change* **6**, 1028–1031 (2016). doi: 10.1038/nclimate3108.

[42] Stauffer, R., Mary, G. J., Messner, J. W., Umlauf, N. & Zeileis, A. Spatio-temporal precipitation climatology over complex terrain using a censored additive regression model. *International Journal of Climatology* **37**, 3264–3275 (2017). doi: 10.1002/joc.4913.

[43] Adebayo, S. B. & Fahrmeir, L. Analysing child mortality in Nigeria with geoadditive discrete-time survival models. *Statistics in Medicine* **24**, 709–728 (2005). doi: 10.1002/sim.1842.

[44] Golding, N. *et al.* Mapping under-5 and neonatal mortality in Africa, 2000-15: a baseline analysis for the Sustainable Development Goals. *Lancet* **390**, 2171–2182 (2017). doi: 10.1016/S0140-6736(17)31758-0.

[45] Harttgen, K., Lang, S., Santer, J. & Seiler, J. Modelling under-five mortality through multilevel structured additive regression with varying coefficients for Asia and sub-Saharan Africa. *Journal of Development Studies* **56**, 401–430 (2020). doi: 10.1080/00220388.2018.1563681.

[46] Kandala, N.-B., Fahrmeir, L., Klasen, S. & Priebe, J. Geo-additive models of childhood undernutrition in three sub-Saharan African countries. *Population, Space and Place* **15**, 461–473 (2009). doi: 10.1002/psp.524.

[47] Osgood-Zimmerman, A. *et al.* Mapping child growth failure in Africa between 2000 and 2015. *Nature* **555**, 41–47 (2018). doi: 10.1038/nature25760.

[48] Seiler, J., Harttgen, K., Kneib, T. & Lang, S. Modelling children's anthropometric status using Bayesian distributional regression merging socio-economic and remote sensed data from South Asia and sub-Saharan Africa. *Economics & Human Biology* **40**, 100950 (2021). doi: 10.1016/j.ehb.2020.100950.

[49] Roberts, D. J., Matthews, G., Snow, R. W., Zewotir, T. & Sartorius, B. Investigating the spatial variation and risk factors of childhood anaemia in four sub-Saharan African countries. *BMC Public Health* **20**, 126 (2020). doi: 10.1186/s12889-020-8189-8.

[50] Kneib, T. & Fahrmeir, L. Structured additive regression for categorical space–time data: a mixed model approach. *Biometrics* **62**, 109–118 (2006). doi: 10.1111/j.1541-0420.2005.00392.x.

[51] Fahrmeir, L. & Tutz, G. *Multivariate Statistical Modelling Based on Generalized Linear Models*. Springer Series in Statistics (Springer New York, NY, New York, 2001), 2 edn. doi: 10.1007/978-1-4757-3454-6.

[52] Gayawan, E., Arogundade, E. D. & Adebayo, S. B. Possible determinants and spatial patterns of anaemia among young children in Nigeria: a Bayesian semi-parametric modelling. *International Health* **6**, 35–45 (2014). doi: 10.1093/inthealth/iht034.

[53] Ngwira, A. & Kazembe, L. N. Analysis of severity of childhood anemia in Malawi: a Bayesian ordered categories model. *Open Access Medical Statistics* **6**, 9–20 (2016). doi: 10.2147/OAMS.S95159.

[54] Koenker, R. *Quantile Regression*. Econometric Society Monographs (Cambridge University Press, 2005). doi: 10.1017/CBO9780511754098.

[55] Rigby, R. A. & Stasinopoulos, D. M. Generalized additive models for location, scale and shape. *Journal of the Royal Statistical Society: Series C (Applied Statistics)* **54**, 507–554 (2005). doi: 10.1111/j.1467-9876.2005.00510.x.

[56] Fasiolo, M., Wood, S. N., Zaffran, M., Nedellec, R. & Goude, Y. Fast calibrated additive quantile regression. *Journal of the American Statistical Association* **116**, 1402–1412 (2021). doi: 10.1080/01621459.2020.1725521.

[57] Fasiolo, M., Wood, S. N., Zaffran, M., Nedellec, R. & Goude, Y. **qgam**: Bayesian nonparametric quantile regression modeling in R. *Journal of Statistical Software* **100**, 1–31 (2021). doi: 10.18637/jss.v100.i09.

[58] Lang, S., Umlauf, N., Wechselberger, P., Harttgen, K. & Kneib, T. Multilevel structured additive regression. *Statistics and Computing* **24**, 223–238 (2014). doi: 10.1007/s11222-012-9366-0.

[59] Umlauf, N., Klein, N. & Zeileis, A. BAMLSS: Bayesian additive models for location, scale, and shape (and beyond). *Journal of Computational and Graphical Statistics* **27**, 612–627 (2018). doi: 10.1080/10618600.2017.1407325.

[60] Umlauf, N. & Kneib, T. A primer on Bayesian distributional regression. *Statistical Modelling* **18**, 219–247 (2018). doi: 10.1177/1471082X18759140.

[61] Sakamoto, Y., Ishiguro, M. & Kitagawa, G. *Akaike Information Criterion Statistics*. Mathematics and its Applications (Springer Dordrecht, 1986), 1 edn.

[62] Gneiting, T. & Raftery, A. E. Strictly proper scoring rules, prediction, and estimation. *Journal of the American Statistical Association* **102**, 359–378 (2007). doi: 10.1198/016214506000001437.

[63] Mayr, A., Fenske, N., Hofner, B., Kneib, T. & Schmid, M. Generalized additive models for location, scale and shape for high dimensional data: a flexible approach based on boosting. *Journal of the Royal Statistical Society C* **61**, 403–427 (2012). doi: 10.1111/j.1467-9876.2011.01033.x.

[64] Thomas, J. *et al.* Gradient boosting for distributional regression: faster tuning and improved variable selection via noncyclical updates. *Statistics and Computing* **28**, 673–687 (2018). doi: 10.1007/s11222-017-9754-6.

[65] Umlauf, N. *et al.* Scalable estimation for structured additive distributional regression. *Journal of Computational and Graphical Statistics* **0**, 1–23 (2024). doi: 10.1080/10618600.2024.2388604.

[66] Murphy, A. H. Skill scores based on the mean square error and their relationships to the correlation coefficient. *Monthly Weather Review* **116**, 2417–2424 (1988). doi: 10.1175/1520-0493(1988)116¡2417:SSBOTM¿2.0.CO;2.

[67] Klein, N., Kneib, T., Klasen, S. & Lang, S. Bayesian structured additive distributional regression for multivariate responses. *Journal of the Royal Statistical Society: Series C (Applied Statistics)* **64**, 569–591 (2015). doi: 10.1111/rssc.12090.

[68] Fahrmeir, L., Kneib, T., Lang, S. & Brian, M. *Regression: Models, Methods and Applications* (Springer Berlin, Heidelberg, 2021), 2 edn. doi: 10.1007/978-3-662-63882-8.

[69] Wood, S. N. Thin plate regression splines. *Journal of the Royal Statistical Society: Series B (Statistical Methodology)* **65**, 95–114 (2003). doi: 10.1111/1467-9868.00374.

[70] Wood, S. N., Li, Z., Shaddick, G. & Augustin, N. H. Generalized additive models for gigadata: modelling the UK black smoke network daily data. *Journal of the American Statistical Association* **112**, 1199–1210 (2017). doi: 10.1080/01621459.2016.1195744.

[71] Hastie, T. J. & Tibshirani, R. J. Varying-coefficient models. *Journal of the Royal Statistical Society. Series B (Methodological)* **55**, 757–796 (1993). http://www.jstor.org/stable/2345993.

[72] Roberts, D. R. *et al.* Cross-validation strategies for data with temporal, spatial, hierarchical, or phylogenetic structure. *Ecography* **40**, 913–929 (2017). doi: 10.1111/ecog.02881.

[73] Stasinopoulos, M. & Rigby, R. **gamlss.dist**: Distributions for generalized additive models for location scale and shape (2021). R package version 5.3-2; https://CRAN.R-project.org/package=gamlss.dist.

[74] Umlauf, N. *et al.* **bamlss**: Bayesian additive models for location scale and shape (and beyond) (2024). R package version 1.1-9 http://CRAN.R-project.org/package=bamlss.

[75] Gamerman, D. Sampling from the posterior distribution in generalized linear mixed models. *Statistics and Computing* **7**, 57–68 (1997). doi: 10.1023/A:1018509429360.

[76] Köhler, M., Umlauf, N. & Greven, S. Nonlinear association structures in flexible Bayesian additive joint models. *Statistics in Medicine* **37**, 4771–4788 (2018). doi: 10.1002/sim.7967.

[77] Umlauf, N., Klein, N., Simon, T. & Zeileis, A. **bamlss**: A lego toolbox for flexible Bayesian regression (and beyond). *Journal of Statistical Software* **100**, 1–53 (2021). doi: 10.18637/jss.v100.i04.

[78] Dunn, P. K. & Smyth, G. K. Randomized quantile residuals. *Journal of Computational and Graphical Statistics* **5**, 236–244 (1996). doi: 10.1080/10618600.1996.10474708.

[79] Dawid, A. P. Present position and potential developments: some personal views statistical theory the prequential approach. *Journal of the Royal Statistical Society: Series A (General)* **147**, 278–290 (1984). doi: 10.2307/2981683.

[80] van Buuren, S. & Fredriks, M. Worm plot: a simple diagnostic device for modelling growth reference curves. *Statistics in Medicine* **20**, 1259–1277 (2001). doi: 10.1002/sim.746.

[81] DHS. Description of the Demographic and Health Surveys Program: the DHS Program (2019). Retrieved from September 26, 2019, https://dhsprogram.com/Data/Guide-to-DHS-Statistics/Description_of_The_Demographic_and_Health_Surveys_Program.htm.

[82] R Core Team. R: A language and environment for statistical computing (2023). https://www.R-project.org/.

[83] Lang, M. & R Core Team. **backports**: Reimplementations of functions introduced since R-3.0.0 (2020). R package version 1.2.1; https://CRAN.R-project.org/package=backports.

[84] Robinson, D., Hayes, A. & Couch, S. **broom**: Convert statistical objects into tidy tibbles (2021). R package version 0.7.8; https://CRAN.R-project.org/package=broom.

[85] Plummer, M., Best, N., Cowles, K. & Vines, K. **CODA**: Convergence diagnosis and output analysis for MCMC. *R News* **6**, 7–11 (2006). https://journal.r-project.org/archive/, https://www.r-project.org/doc/Rnews/Rnews_2006-1.pdf.

[86] Tierney, L. **codetools**: Code analysis tools for R (2020). R package version 0.2-18; https://CRAN.R-project.org/package=codetools.

[87] Zeileis, A., Hornik, K. & Murrell, P. Escaping RGBland: selecting colors for statistical graphics. *Computational Statistics & Data Analysis* **53**, 3259–3270 (2009). doi: 10.1016/j.csda.2008.11.033.

[88] Zeileis, A. *et al.* **colorspace**: A toolbox for manipulating and assessing colors and palettes. *Journal of Statistical Software* **96**, 1–49 (2020). doi: 10.18637/jss.v096.i01.

[89] Turner, R. **deldir**: Delaunay triangulation and dirichlet (voronoi) tessellation (2021). R package version 1.0-6; https://CRAN.R-project.org/package=deldir.

[90] Hijmans, R. J., Phillips, S., Leathwick, J. & Elith, J. **dismo**: Species distribution modeling (2020). R package version 1.3-3; https://CRAN.R-project.org/package=dismo.

[91] Original S code by Richard A. Becker and Allan R. Wilks. R version by Ray Brownrigg. Enhancements by Thomas P. Minka and Alex Deckmyn. **maps**: Draw geographical maps (2018). R package version 3.3.0; https://CRAN.R-project.org/package=maps.

[92] Pinheiro, J. C. & Bates, D. M. *Mixed-Effects Models in S and S-PLUS* (Springer, New York, 2000). doi: 10.1007/b98882.

[93] Pinheiro, J. C., Bates, D. M. & R Core Team. **nlme**: Linear and nonlinear mixed effects models (2022). R package version 3.1-159; https://CRAN.R-project.org/package=nlme.

[94] Müller, K. & Wickham, H. **pillar**: Coloured formatting for columns (2021). R package version 1.6.1; https://CRAN.R-project.org/package=pillar.

[95] Hijmans, R. J. **raster**: Geographic data analysis and modeling (2021). R package version 3.4-13; https://CRAN.R-project.org/package=raster.

[96] Bivand, R. & Rundel, C. **rgeos**: Interface to geometry engine - open source ('GEOS') (2022). R package version 0.6-1; https://CRAN.R-project.org/package=rgeos.

[97] Bivand, R., Keitt, T. & Rowlingson, B. **rgdal**: Bindings for the 'geospatial' data abstraction library (2022). R package version 1.6-3; https://CRAN.R-project.org/package=rgdal.

[98] South, A. **rnaturalearth**: World map data from natural earth (2017). R package version 0.1.0; https://CRAN.R-project.org/package=rnaturalearth.

[99] South, A. **rnaturalearthdata**: World vector map data from natural earth used in 'rnaturalearth' (2017). R package version 0.1.0; https://CRAN.R-project.org/package=rnaturalearthdata.

[100] South, A. **rnaturalearthhires**: High resolution world vector map data from natural earth used in **rnaturalearth** (2023). URL: https://docs.ropensci.org/rnaturalearthhires, https://github.com/ropensci/rnaturalearthhires.

[101] Wickham, H. & Seidel, D. **scales**: Scale functions for visualization (2020). R package version 1.1.1; https://CRAN.R-project.org/package=scales.

[102] Jordan, A., Krüger, F. & Lerch, S. Evaluating probabilistic forecasts with **scoringRules**. *Journal of Statistical Software* **90**, 1–37 (2019). doi: 10.18637/jss.v090.i12.

[103] Pebesma, E. J. Simple features for R: Standardized support for spatial vector data. *The R Journal* **10**, 439–446 (2018). doi: 10.32614/RJ-2018-009.

[104] Strimas-Mackey, M. **smoothr**: Smooth and tidy spatial features (2021). R package version 0.2.2; https://CRAN.R-project.org/package=smoothr.

[105] Pebesma, E. J. & Bivand, R. S. Classes and methods for spatial data in R. *R News* **5**, 9–13 (2005). https://CRAN.R-project.org/doc/Rnews/.

[106] Bivand, R. S., Pebesma, E. J. & Gómez-Rubio, V. *Applied Spatial Data Analysis with R*. Use R! (Springer New York, NY, 2013), 2 edn. https://asdar-book.org/, doi: 10.1007/978-1-4614-7618-4.

[107] WHO Global Health Observatory. Prevalence of anaemia in children aged 6–59 months (%) [Dataset] (2022). Retrieved May 13, 2024, from https://www.who.int/data/gho/data/indicators/indicator-details/GHO/prevalence-of-anaemia-in-children-under-5-years-(-).

[108] Stevens, G. A. *et al.* National, regional, and global estimates of anaemia by severity in women and children for 2000–19: a pooled analysis of population-representative data. *Lancet Global Health* **10**, e627–e639 (2022). doi: 10.1016/S2214-109X(22)00084-5.

[109] United Nations Population Division. World population prospects 2022 [Dataset] (2022). Retrieved May 13, 2024, from https://population.un.org/wpp/Download/Standard/Population/.

[110] Fahim, O. *et al.* Double burden of malnutrition in Afghanistan: Secondary analysis of a national survey. *PLOS ONE* **18**, 1–19 (2023). doi: 10.1371/journal.pone.0284952.

[111] Stanikzai, M. H., Zakir, S., Ishaq, N. & Rahimi, B. A. Prevalence of anemia and its associated factors among children under 5 years of age attending a comprehensive healthcare facility in Kandahar city, Afghanistan. *Indian Journal of Public Health* **66** (2022). doi: 10.4103/ijph.ijph_2202_210.

[112] Campbell, R. K. *et al.* Epidemiology of anaemia in children, adolescent girls, and women in Bhutan. *Maternal & Child Nutrition* **14**, e12740 (2018). doi: 10.1111/mcn.12740.

[113] Chhetri, K., Mynak, M. L. & Pedon, K. Anemia and risk factors among children 6 months to 59 months old: a hospital-based prospective study. *Bhutan Health Journal* **3**, 1–4 (2017). doi: 10.47811/bhj.45.

[114] Leepile, T. T. *et al.* Anemia prevalence and anthropometric status of indigenous women and young children in rural Botswana: The San People. *Nutrients* **13** (2021). doi: 10.3390/nu13041105.

[115] Vonaesch, P. *et al.* Factors associated with stunted growth in children under five years in Antananarivo, Madagascar and Bangui, Central African Republic. *Maternal and Child Health Journal* **25**, 1626–1637 (2021). doi: 10.1007/s10995-021-03201-8.

[116] Thorne, C. J. *et al.* Anaemia and malnutrition in children aged 0–59 months on the Bijagós Archipelago, Guinea-Bissau, West Africa: a cross-sectional, population-based study. *Paediatrics and International Child Health* **33**, 151–160 (2013). doi: 10.1179/2046905513Y.0000000060.

[117] Silva, C. & Abecasis, A. Malnutrition in Guinea-Bissau: a country wide representative cross-sectional study. *European Journal of Public Health* **34**, ckae144.239 (2024). doi: 10.1093/eurpub/ckae144.239.

[118] Ncogo, P. *et al.* Prevalence of anemia and associated factors in children living in urban and rural settings from Bata District, Equatorial Guinea, 2013. *PLOS ONE* **12**, 1–14 (2017). doi: 10.1371/journal.pone.0176613.

[119] Habib, M. A. *et al.* Prevalence and predictors of iron deficiency anemia in children under five years of age in Pakistan, a secondary analysis of national nutrition survey data 2011-2012. *PLOS ONE* **11**, 1–13 (2016). doi: 10.1371/journal.pone.0155051.

[120] Habib, A. *et al.* Prevalence and risk factors for iron deficiency anemia among children under five and women of reproductive age in Pakistan: Findings from the national nutrition survey 2018. *Nutrients* **15** (2023). doi: 10.3390/nu15153361.

[121] Wirth, J. P. *et al.* Risk factors of anaemia and iron deficiency in Somali children and women: Findings from the 2019 Somalia micronutrient survey. *Maternal & Child Nutrition* **18**, e13254 (2022). URL https://onlinelibrary.wiley.com/doi/abs/10.1111/mcn.13254.

[122] Zavala, E., Adler, S., Wabyona, E., Ahimbisibwe, M. & Doocy, S. Trends and determinants of anemia in children 6-59 months and women of reproductive age in Chad from 2016 to 2021. *BMC Nutrition* **9**, 117 (2023). doi: 10.1186/s40795-023-00777-y.

[123] UN Refugee Agency. Congo, Dem. Rep.: Standardised Expanded Nutrition Survey (refugees from Burundi, Central African Republic and South Sudan) (2019). Retrieved November 4, 2024, from https://microdata.unhcr.org/index.php/catalog/782/download/2718.

[124] UN Refugee Agency. Nepal: Standardised Expanded Nutrition Survey (Bhutanese Refugee Camps) (2019). Retrieved November 7, 2024, from https://microdata.unhcr.org/index.php/catalog/521/download/2013.

[125] UN Refugee Agency. Botswana: Standardised Expanded Nutrition Survey (Dukwi) (2013). Retrieved November 4, 2024, from https://microdata.unhcr.org/index.php/catalog/663/download/2429.

[126] UN Refugee Agency. South Sudan: Standardised Expanded Nutrition Survey (Makpandu refugee camps) (2019). Retrieved November 4, 2024, from https://microdata.unhcr.org/index.php/catalog/596/download/2185.

[127] UN Refugee Agency. Congo, Dem. Rep.: Standardised Expanded Nutrition Survey (refugees from Burundi, Central African Republic and South Sudan) (2019). Retrieved November 4, 2024, from https://microdata.unhcr.org/index.php/catalog/782/download/2717.

[128] World Food Programme. Urban in-depth EFSA Djibouti (2011). Retrieved October 31, 2024, from https://citeseerx.ist.psu.edu/document?repid=rep1&type=pdf&doi=8fbffed2efa0a5883a022756f7da1bfd64a49cb1.

[129] Office Nationale de la Statistique (ONS), Ministère de la Santé (MS) & ICF. Enquête Démographique et de Santé en Mauritanie 2019-2021: rapport de synthèse (2011). Retrieved October 31, 2024, from https://dhsprogram.com/pubs/pdf/FR373/FR373.pdf.

[130] FSNAU, FAO & UCL. National Micronutrient and Anthropometric Nutrition Survey Somalia (2009). Retrieved November 6, 2024, from https://fsnau.org/downloads/Somalia-National-Micronutrient-Study.pdf.

[131] UN Refugee Agency. Ethiopia: Standardised Expanded Nutrition Survey (Melkadida) (2018). Retrieved November 6, 2024, from https://microdata.unhcr.org/index.php/catalog/114/download/331.

[132] UN Refugee Agency. Congo, Dem. Rep.: Standardised Expanded Nutrition Survey (refugees from Burundi, Central African Republic and South Sudan) (2019). Retrieved November 4, 2024, from https://microdata.unhcr.org/index.php/catalog/782/download/2719.

[133] UN Refugee Agency. South Sudan: Standardised Expanded Nutrition Survey (Gorom refugee camp) (2019). Retrieved November 4, 2024, from https://microdata.unhcr.org/index.php/catalog/584/download/2194.

[134] UN Refugee Agency. South Sudan: Standardised Expanded Nutrition Survey (Jamjang refugee camp) (2019). Retrieved November 4, 2024, from https://microdata.unhcr.org/index.php/catalog/590/download/2168.

[135] UN Refugee Agency. South Sudan: Standardised Expanded Nutrition Survey (Maban refugee camps) (2019). Retrieved November 4, 2024, from https://microdata.unhcr.org/index.php/catalog/595/download/2179.

[136] UN Refugee Agency. Chad: Standardised Expanded Nutrition Survey (South, South-East and West) (2016). Retrieved November 4, 2024, from https://microdata.unhcr.org/index.php/catalog/665/download/2427.

[137] UN Refugee Agency. Chad: Standardised Expanded Nutrition Survey (East, South and Lake Region) (2021). Retrieved November 4, 2024, from https://microdata.unhcr.org/index.php/catalog/775/download/2697.
