# Supplementary material for: High-resolution spatial prediction of anemia risk among children aged 6 to 59 months in low- and middle-income countries
Source: Commun Med (Lond). 2025 Mar 4;5:57. doi: 10.1038/s43856-025-00765-2 (PMC11880423; doi:10.1038/s43856-025-00765-2)
Supplement: Supplementary file 3 — Description of Additional Supplementary Files [file 43856_2025_765_MOESM3_ESM.pdf]

Description of Additional Supplementary Files

- File Name: Supplementary Software
  The custom R-code tailored to the HPC infrastructure LEO of the University Innsbruck which was used to perform the statistical analysis. This file also includes a README with additional information.

- File Name: Supplementary Data 1
  The underlying data to reproduce Figure 1 using the R-script figures.R included in the Supplementary software.

- File Name: Supplementary Data 2
  The underlying data to reproduce Figure 2 using the R-script figures.R included in the Supplementary software.

- File Name: Supplementary Data 3
  The underlying data to reproduce Figure 3 using the R-script figures.R included in the Supplementary software.

- File Name: Supplementary Data 4
  The underlying data to reproduce Figure 4 using the R-script figures.R included in the Supplementary software.

- File Name: Supplementary Data 5
  The underlying data to reproduce Figure 5 using the R-script figures.R included in the Supplementary software.

- File Name: Supplementary Data 6
  The underlying data to reproduce Figure 6 using the R-script figures.R included in the Supplementary software.

Universität Innsbruck, Universitätsstraße 15, 6020 Innsbruck, Austria
Phone: +43 512 507-70428
Email: Johannes.Seiler@uibk.ac.at
